# Genome-Wide Analysis of Cell Type-Specific Gene Transcription during Spore Formation in *Clostridium difficile*

**DOI:** 10.1371/journal.pgen.1003756

**Published:** 2013-10-03

**Authors:** Laure Saujet, Fátima C. Pereira, Monica Serrano, Olga Soutourina, Marc Monot, Pavel V. Shelyakin, Mikhail S. Gelfand, Bruno Dupuy, Adriano O. Henriques, Isabelle Martin-Verstraete

**Affiliations:** 1 Laboratoire Pathogenèse des Bactéries Anaérobies, Institut Pasteur, Paris, France; 2 Université Paris Diderot, Sorbonne Paris Cité, Cellule Pasteur, Paris, France; 3 Microbial Development Laboratory, Instituto de Tecnologia Química e Biológica, Universidade Nova de Lisboa, Oeiras, Portugal; 4 Institute for Information Transmission Problems, RAS, Bolshoi Karetny per, 19, Moscow, Russia; 5 M.V. Lomonosov Moscow State University, Faculty of Biengineering and Bioinformatics, Vorobievy Gory 1-73, Moscow, Russia; University of Geneva Medical School, Switzerland

## Abstract

*Clostridium difficile*, a Gram positive, anaerobic, spore-forming bacterium is an emergent pathogen and the most common cause of nosocomial diarrhea. Although transmission of *C. difficile* is mediated by contamination of the gut by spores, the regulatory cascade controlling spore formation remains poorly characterized. During *Bacillus subtilis* sporulation, a cascade of four sigma factors, σ^F^ and σ^G^ in the forespore and σ^E^ and σ^K^ in the mother cell governs compartment-specific gene expression. In this work, we combined genome wide transcriptional analyses and promoter mapping to define the *C. difficile* σ^F^, σ^E^, σ^G^ and σ^K^ regulons. We identified about 225 genes under the control of these sigma factors: 25 in the σ^F^ regulon, 97 σ^E^-dependent genes, 50 σ^G^-governed genes and 56 genes under σ^K^ control. A significant fraction of genes in each regulon is of unknown function but new candidates for spore coat proteins could be proposed as being synthesized under σ^E^ or σ^K^ control and detected in a previously published spore proteome. SpoIIID of *C. difficile* also plays a pivotal role in the mother cell line of expression repressing the transcription of many members of the σ^E^ regulon and activating *sigK* expression. Global analysis of developmental gene expression under the control of these sigma factors revealed deviations from the *B. subtilis* model regarding the communication between mother cell and forespore in *C. difficile*. We showed that the expression of the σ^E^ regulon in the mother cell was not strictly under the control of σ^F^ despite the fact that the forespore product SpoIIR was required for the processing of pro-σ^E^. In addition, the σ^K^ regulon was not controlled by σ^G^ in *C. difficile* in agreement with the lack of pro-σ^K^ processing. This work is one key step to obtain new insights about the diversity and evolution of the sporulation process among Firmicutes.

## Author Summary

*Clostridium difficile*, a major cause of antibiotic-associated diarrhea, produces resistant spores that facilitate the persistence of this bacterium in the environment including hospitals. Its transmission is mediated by contamination of gut by spores. Understanding how this complex developmental process is regulated is fundamental to decipher *C. difficile* transmission and pathogenesis. The regulatory cascade controlling sporulation that involves four sigma factors, σ^F^ and σ^G^ in the forespore and σ^E^ and σ^K^ in the mother cell remains poorly characterized in *C. difficile*. By combining transcriptome analysis and promoter mapping, we identified genes expressed under the specific control of each sigma factor. Among sporulation-controlled proteins detected in spore, we can propose candidates for new spore coat proteins important for spore resistance. We also showed differences in the intercompartment communication between forespore and mother cell in *C. difficile* compared to the *Bacillus subtilis* model. In *C. difficile*, we observed that the activation of the σ^E^ regulon was partially independent of σ^F^ and that the σ^K^ regulon was not controlled by σ^G^. Our finding suggests that the *C. difficile* sporulation process might be more ancestral compared to that of *B. subtilis*.

## Introduction

*Clostridium difficile*, a Gram positive, anaerobic, spore-forming bacterium is a major cause of nosocomial infections associated with antibiotic therapy and is a major burden to health care services. This enteropathogen can lead to antibiotic-associated diarrhea and pseudo-membranous colitis, a potentially lethal disease. Two large toxins, the enterotoxin TcdA and the cytotoxin TcdB are the main virulence factors required for the development of symptoms of *C. difficile* infection (CDI). *C. difficile* produces highly resistant spores that facilitate the persistence of this bacterium in the environment in particular in aerobic conditions and contaminate hospital environments contributing to the establishment of a reservoir. Transmission of *C. difficile* is further mediated by contamination of the gut by spores as demonstrated recently using a murine model for CDI [1], [2]. The disruption of the colonic microflora by antimicrobial therapy precipitates CDI and colonization of the intestinal tract. An early event towards this colonization is the germination process that converts dormant spores into vegetative cells that multiply leading to the production of toxins [3]. Glycine and bile salts like sodium cholate or sodium taurocholate are co-germinants required to induce *C. difficile* spores germination [4]. However, the molecular mechanisms involved in sporulation and germination are still poorly studied in *C. difficile* and our current knowledge on these processes is based mainly on data derived from the studies on *Bacillus subtilis*.

At the onset of sporulation in *B. subtilis*, sporulating cells undergo an asymmetric division which partitions the sporangial cell into a larger mother cell and a smaller forespore (the future spore). The forespore is next wholly engulfed by the mother cell and later the dormant spore is released from the mother cell by lysis [5]. The developmental program of sporulation is mainly governed by the sequential activation of four sigma factors: σ^F^, σ^E^, σ^G^ and σ^K^. Their activity is confined to the forespore for σ^F^ and σ^G^ and to the mother cell for σ^E^ and σ^K^. Compartmentalization of gene expression is coupled to morphogenesis with σ^F^ and σ^E^ becoming active after asymmetric division and σ^G^ and σ^K^ after completion of engulfment of the forespore by the mother cell [5], [6], [7].

In *B. subtilis*, coordinated changes in gene expression underlie morphological differentiation in both the predivisional sporangium and later in the two compartments with the existence of communication between the mother cell and the forespore. The response regulator, Spo0A, and a phosphorelay involving five kinases, intermediary phosphorylated proteins and phosphatases control sporulation initiation. The alternative sigma factor, σ^H^, which transcribes *spo0A* and *sigF* also controls early sporulation steps. When Spo0A-P level reaches a critical threshold, Spo0A-P activates sporulation genes including *spoIIE* as well as both the *spoIIAA*-*spoIIAB*-*sigF* and the *spoIIGA-sigE* operons encoding σ^F^ and σ^E^, respectively [8]. After its synthesis, σ^F^ is held inactive by the anti-sigma factor SpoIIAB until the phosphatase SpoIIE dephosphorylates the anti-anti sigma factor SpoIIAA leading to the release of an active σ^F^ from SpoIIAB after completion of asymmetric cell division [5]. σ^F^ then transcribes about 50 genes in the forespore [9], [10] including *spoIIR* encoding a secretory protein required for the processing of pro-σ^E^ into active σ^E^ in the mother cell [11]. σ^E^ regulates in turn the expression of mother cell specific genes [9], [12], [13] and activates *sigK* expression with the combined activity of the SpoIIID regulator [14]. In the forespore, σ^F^ also controls *sigG* transcription. However, σ^G^ becomes active coincidently with the completion of forespore engulfment by the mother cell. Following engulfment completion, the σ^E^-controlled SpoIIIA proteins, together with the forespore-specific SpoIIQ protein, are required to maintain the potential for macromolecular synthesis in the forespore [6]. The RNA polymerase σ^G^ holoenzyme transcribed 95 genes in the forespore including proteins like SpoIVB or CtpB involved in the processing and activation of σ^K^ the last factor sigma of sporulation [5], [6].

The four sporulation specific sigma factors are conserved in Clostridia and are present in *C. difficile* [15], [16], [17], [18], [19], [20]. This is also the case for key genes involved in the spore morphogenesis [16], [17], [20]. In *Clostridium acetobutylicum* and *Clostridium perfringens*, the role of sporulation sigma factors in spore morphogenesis has been analyzed [21], [22], [23], [24]. No resistant spores are formed by the *sigF* and *sigG* mutants of both Clostridia and by the *sigE* mutant of *C. acetobutylicum* whereas the *sigE* and *sigK* mutants of *C. perfringens* are severely defective in their ability to sporulate. Interestingly, *sigE* and *sigF* mutants of *C. acetobutylicum* fail to form the asymmetric division septum [22], [24] while a *sigK* mutant is blocked earlier in sporulation than a *sigE* mutant in *C. perfringens* [21]. A detailed study of the morphological changes during the *C. difficile* spore differentiation process has recently been performed [19]. The *C. difficile sigF* and *sigE* mutants are arrested at the asymmetric stage while the *sigG* mutant is blocked after the completion of engulfment but unlike in *B. subtilis*, shows deposition of electrodense coat material around the forespore. These mutants are unable to sporulate [19]. A *sigK* mutant of *C. difficile* forms four orders of magnitude fewer heat resistant spores than the isogenic wild-type strain. While showing no signs of coat deposition around the developing spore, this mutant shows accumulation of at least some cortex material unlike the case for *B. subtilis* [19]. The characterization of the *sig* sporulation mutants in several Clostridia suggests deviations from the *B. subtilis* paradigm regarding the function of the sporulation sigma factors [19], [20], [21], [22], [24], [25].

The regulatory cascade controlling spore formation is also still poorly characterized in Clostridia compared to *B. subtilis*. The global analysis of the *C. acetobutylicum* transcriptional program during sporulation has given the dynamic orchestration of expression of the sporulation sigma factors and of key sporulation genes in this Clostridia [26] but the definition of the four-sigma factors regulons remains to be determined. In *C. difficile*, recent data have been obtained on sporulation initiation. Both Spo0A and σ^H^ are present and required for efficient *C. difficile* sporulation [27], [28]. By contrast, the phosphorelay is absent and the sporulation initiation pathway remains in *C. difficile* and other Clostridia as a two-component system with Spo0A and associated kinases [18], [28], [29]. Both σ^H^ and Spo0A directly control the expression of the *spoIIAA*-*spoIIAB*-*sigF* operon while Spo0A binds *in vitro* to the promoter region of *spoIIAA*, *spoIIE* and *spoIIGA* (in operon with *sigE*) [27], [30]. By contrast, little is known about the molecular mechanisms controlling later steps in the sporulation regulatory cascade in *C. difficile*. In this work, we combined transcriptome, genome-wide transcriptional start site (TSS) mapping and *in silico* identification of promoters to define the σ^F^, σ^E^, σ^G^ and σ^K^ regulons. This helped us to propose candidates for new spore coat proteins. Finally, we also identified interesting differences in the regulatory cascade of *C. difficile* compared to the *B. subtilis* model. We showed that activation of the σ^E^ regulon was partially independent of σ^F^ and that the σ^K^ regulon was not controlled by σ^G^.

## Results and Discussion

### I. Analysis of the sporulation regulatory network by global approaches

#### Overview of the transcriptome data

The *C. difficile* mutants inactivated for σ^F^, σ^E^, σ^G^ or σ^K^ have been recently constructed [19]. The *sigF* and *sigE* mutants are blocked at stage II of sporulation. The *sigG* mutant is blocked after the completion of engulfment and the *sigK* mutant form spores lacking coat material [19]. Thus, the sequential appearance of events seems to be similar to that observed in *B. subtilis*: σ^F^ and σ^E^ control early stages of development and are likely replaced by σ^G^ and σ^K^ later during sporulation. These studies have also shown the confinement of the activities of σ^F^ and σ^G^ to the forespore, and those of σ^E^ and σ^K^ to the mother cell [19]. To investigate more in detail the sporulation regulatory cascade in *C. difficile* and to define the regulons controlled by the four cell type-specific RNA polymerase sigma factors, we compared the expression profiles of the wild-type 630Δerm strain and of the *sigF*, *sigE*, *sigG* or *sigK* mutant. We performed preliminary tests to determine conditions allowing detection of differential expression between the 630Δerm strain and each mutant inactivated for a sigma factor as described in Materials and Methods. The cells were harvested 14 h after inoculation for the wild-type strain 630Δerm and the *sigF* or the *sigE* mutant, after 19 h of growth for strain 630Δerm and the *sigG* mutant or after 24 h of growth for strain 630Δerm and the *sigK* mutant. We found 111 genes and 141 genes differentially expressed with a p value <0.05 between the 630Δerm strain and the *sigF* mutant or the *sigE* mutant, respectively (Table S1 and S2). While 92 and 19 genes were down and upregulated in the *sigF* mutant compared to the 630Δerm strain, all the 141 genes were downregulated in the *sigE* mutant. In addition, 51 and 66 genes were differentially expressed with a p value <0.05 between the 630Δerm strain and the *sigG* or the *sigK* mutant in transcriptome, respectively (Table S3 and S4). All of the presumptive *sigG*-controlled genes were downregulated in the mutant compared to the 630Δerm strain. Finally, 58 and 8 genes were down and upregulated in the *sigK* mutant relative to the 630Δerm strain. To confirm the results obtained in the microarrays experiments, we performed qRT-PCR on a subset of 10 to 15 genes representative of various cell functions and regulated by each of the cell type-specific sigma factors. The qRT-PCR results confirmed the microarrays data for all of the tested genes (Table S5).

#### Mapping of transcriptional start sites of genes controlled by sporulation sigma factors

**Figure 1:**
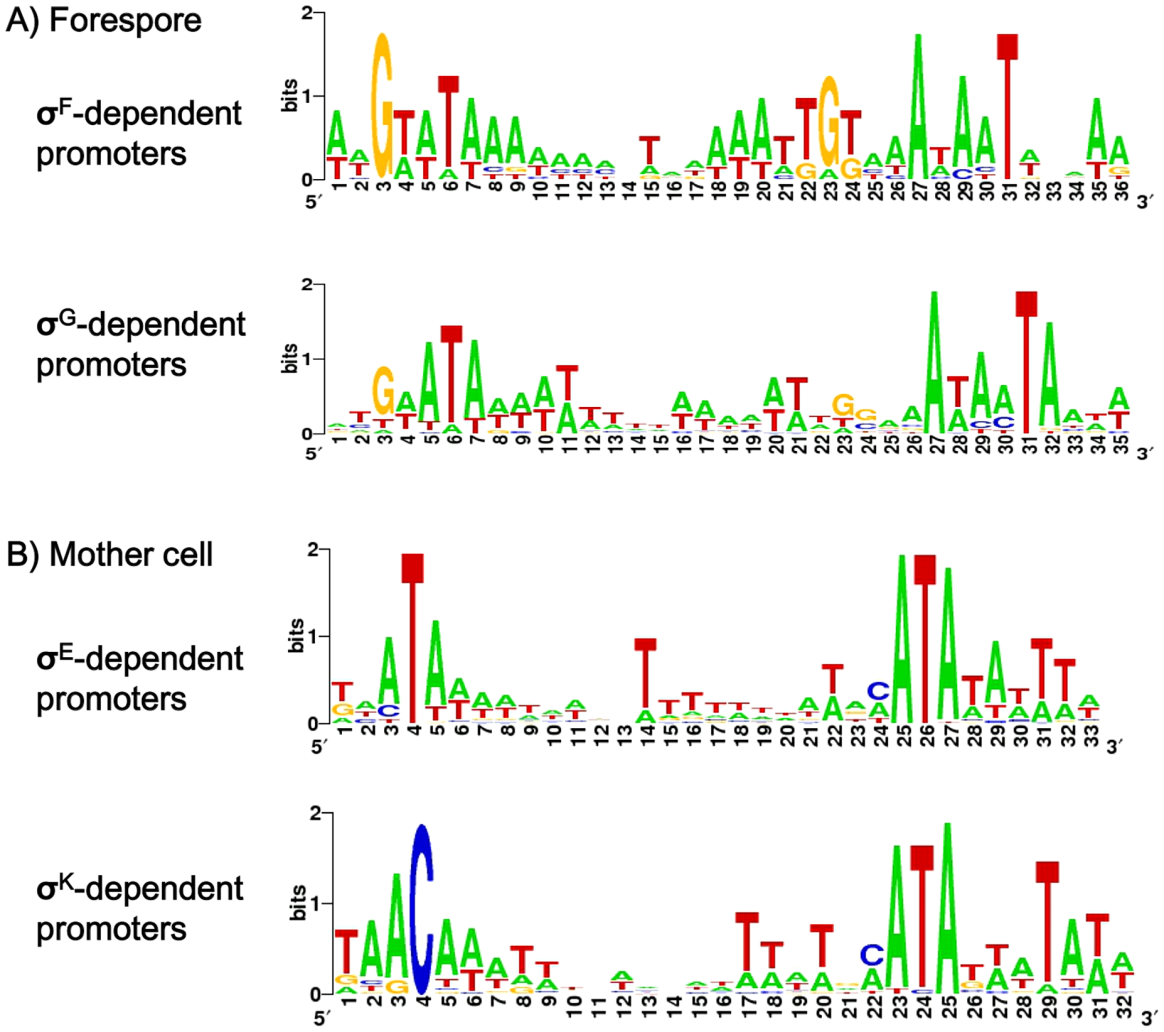
Consensus sequences of σ^F^, σ^E^, σ^G^ or σ^K^-dependent promoters in *C. difficile*. Consensus promoter sequences for σ^F^- and σ^G^-controlled promoters (A) or σ^E^- and σ^K^-controlled promoters (B) in *C. difficile*. The sequence logo was created on the WebLogo website (http://weblogo.berkeley.edu) using promoters mapped in this study and listed in Tables S6 and Table S7: compilation of 10 common motifs upstream of σ^F^-regulated genes, of 30 common elements upstream of σ^G^-regulated genes, of 47 conserved sequences upstream of σ^E^-regulated genes or 24 conserved elements σ^K^-regulated genes. The height of the letters is proportional to their frequency.

We recently performed a genome wide determination of transcriptional start sites (TSS) for the 630Δerm strain using RNA-seq [31]. We searched in our data for TSS of all the genes found to be controlled by σ^F^, σ^E^, σ^G^ or σ^K^ in the transcriptome analysis. By this global approach, we mapped about 111 TSS upstream of genes positively controlled by at least one of the four sporulation-specific sigma factors (Table S6 and S7). We analyzed these data by an iterative *in silico* strategy as described in Materials and Methods. We further manually analyzed all promoters not found by this *in silico* procedure (<30%). These strategies allowed us to identify promoters for each sigma factor (Table S6, S7 and S8). We identified about 40 promoters likely to be utilized by RNA polymerase associated to one of the forespore sigma factors (σ^F^ or σ^G^) (Table S6). The consensus elements recognized by σ^F^ in *C. difficile* were determined by using 10 mapped promoters of genes specifically controlled by σ^F^ in transcriptome (downregulated in a *sigF* mutant but not in the *sigE*, *sigG* or *sigK* mutant) (Table S6). The conserved motifs for σ^G^-dependent promoters were defined from a dataset of 30 promoters located upstream of genes specifically controlled by σ^G^ in transcriptome (downregulated in a *sigG* mutant but not in a *sigK* mutant) (). The *in silico* analysis identified 9 of 10 σ^F^ promoters and 23 of 30 σ^G^ promoters. The consensus sequences for *C. difficile* σ^F^- and σ^G^-controlled promoters (Fig. 1A) are very similar to those of *B. subtilis* [10], [16] and to each other in their −10 and −35 elements as noted for *B. subtilis* [10]. However, they differ in that a G is conserved on the upstream side of the −10 promoter element in σ^F^-dependent promoters (position 23 in Fig. 1A) whereas for σ^G^-dependent promoters a A is conserved on the downstream side of the −10 element (position 32 in Fig. 1A).

Upstream of genes specifically controlled by σ^E^ (downregulated in a *sigE* mutant but neither in a *sigK* mutant nor in a *sigG* mutant), we identified 47 promoters, 25 of which were seen in the *in silico* analysis (Table S7 and S8). Moreover, we mapped 24 TSS corresponding to promoters recognized by σ^K^ upstream of σ^K^-controlled genes in transcriptome, 19 of which were found *in silico*. The *C. difficile* consensus elements specific for σ^E^- and σ^K^-controlled promoters are very similar to those determined in *B. subtilis* [12], [14], [16]. The motif recognized by σ^E^ and σ^K^ shared very similar −10 sequences while the −35 elements differed with an ATA motif for σ^E^ and AC for σ^K^ (Fig. 1B). In *B. subtilis*, the specificity of interaction of these sigma factors with the −35 region sequences is associated with the presence of a glutamine at position 217 of σ^E^ and an arginine in σ^K^. Indeed, the replacement of glutamine 217 in σ^E^ by an arginine allows σ^E^ to recognize σ^K^-controlled genes [32]. Interestingly, σ^E^ and σ^K^ of *C. difficile* contain a glutamine and an arginine at position 218, respectively (Fig. S1). This observation supports our inference that a conserved T or C is an important feature of the −35 element herein identified for *C. difficile* σ^E^ and σ^K^ promoters.

To date, less than 10 promoters of sporulation-regulated genes have been characterized in Clostridia, mainly in *C. acetobutylicum* (*spoIIGA*, *sigG*, *sigK*) and *C. perfringens* (*spoIIGA*, *cpe*, *sigK*) [33], [34]. This work greatly increased the number of characterized promoters for clostridial sporulation genes. The probability to observe orthologs with high-scoring candidate promoters in many clostridial genomes (Table S8) is highest for genes regulated both in *C. difficile* and *B. subtilis* [9], [10], [12], [14], as these genes likely form the regulon cores.

### II. An overview of the four compartment-specific σ^F^, σ^E^, σ^G^ and σ^K^ regulons

We combined transcriptome data and promoter identifications to define the σ^F^, σ^E^, σ^G^ and σ^K^ regulons and to determine the pool of genes probably under the direct control of each sigma factor among genes positively controlled by these four sigma factors (Table 1 and 2).

**Table 1:** The forespore line of expression with the identification of the σ^F^ and σ^G^ regulon.

Gene	Name	Function	Expression ratio in transcriptome^a^	Promoter^b^	Detection in spore c	operon
**Members of the σ^F^ regulon**			***sigF*/630Δ*erm***			
**sporulation**						
CD0125	*spoIIQ*	Stage II sporulation protein Q	0.11	σ^F^	**−**	
CD2470	*gpr*	Spore endopeptidase	0.22	σ^F^	**+**	*CD2470-CD2468*
CD2469	*spoIIP*	Stage II sporulation protein P	0.14		**−**	
CD2468		Conserved hypothetical protein	0.28		**−**	
CD3564	*spoIIR*	Pro-SigE endopeptidase signalling protein	0.43	σ^F^	**−**	*CD3564-CD3563*
CD3563	*sleB*	Spore-cortex-lytic protein	0.16		**−**	
CD0783	*spoIVB*'	SpoIVB protein, S55 peptidase family	0.10		**−**	
**envelopes**						
CD2141		Serine-type D-Ala-D-Ala carboxypeptidase	0.30		**−**	
CD1229		Putative peptidoglycan glycosyltransferase	0.42	σ^F^	**−**	
CD2686		Putative membrane protein	0.33	σ^F^	**−**	
CD2856		Putative membrane protein	0.46	σ^F^	**−**	
CD2107		Xanthine/uracil/thiamine permease family	0.37		**−**	
CD2102		Putative Na(+)/H(+) antiporter	0.49	σ^A^	**−**	
**miscellaneous**						
CD0580	*gapN*	Glyceraldehyde-3-phosphate dehydrogenase	0.36	σ^F^	**+**	
CD3595		Aminopeptidase	0.55	σ^F^	**+**	
CD2661		Putative peptidase, M16 family	0.47		**+**	*CD2661-CD2660*
CD2660		Putative peptidase, M16 family	0.48		**+**	
CD0047	*ispD*	2-C-methyl-D-erythritol 4-P cytidylyltransferase	0.48	σ^A^	**−**	*CD0047-CD0048*
CD0048	*ispF*	2-C-methyl-D-erythritol 2,4-cyclodiphosphate synthase	0.50		**−**	
CD3220		Putative methyltransferase	0.39	σ^A^	**−**	
CD0761		Putative ATP-dependent RNA helicase	0.46	σ^F^ and σ^A^	**+**	
CD1323	*tepA*	Protein export-enhancing factor	0.38		**+**	
**proteins of unknown function**						
CD0347		Conserved hypothetical protein	0.35	σ^F^	**−**	*CD0347-CD0348*
CD0348		Fragment of conserved hypothetical protein	0.35		**−**	
CD2687		Conserved hypothetical protein	0.36		**−**	

Gene	Name	Function		Expression ratio in transcriptome^a^		Promoter^b^	Detection in spore c	operon
Members of the σ^G^ regulon				*sigF*/630Δ*erm*	*sigG*/630 Δ*erm*			
**sporulation**								
CD0773	*spoVAC*		DPA uptake protein, SpoAC	0.24	0.26	σ^G^	**−**	*CD0773-CD775*
CD0774	*spoVAD*		DPA uptake protein, SpoAD	0.27	0.3		**+**	
CD0775	*spoVAE*		DPA uptake protein, SpoAE	0.32	0.28		**−**	
CD2688	*sspA*		Small, acid-soluble spore protein alpha	0.00	0.02	σ^G^	**+**	
CD3249	*sspB*		Small, acid-soluble spore protein alpha	0.01	0.07	σ^G^	**+**	
CD1290			Putative small acid-soluble spore protein	0.29	0.52	σ^G^	**−**	
CD3220.1			Small acid-soluble spore protein	0.28	0.66	σ^G^	**+**	
CD3499	*spoVT*		Transcriptional regulator, SpoVT	0.07	0.22	σ^F^/σ^G^?	**+**	
CD1213	*spoIVB*		Stage IVB sporulation protein B, peptidase		0.6	σ^G^	**−**	
**envelopes**								
CD1430	*pdaA*		Putative ∂-lactam-biosynthetic deacteylase	0.06	0.21	σ^G^	**−**	
CD1291	*dacF*		D-alanyl-D-alanine carboxypeptidase	0.11	0.31	σ^G^	**+**	
CD0784			Putative N-acetylmuramoyl-L-alanine amidase	0.23	0.41		**−**	
CD2762	*uppS*		Undecaprenyl pyrophosphate synthetase	0.20	0.62	σ^G^	**−**	
CD0793			Putative membrane protein, DUF81 family	0.26	0.33		**−**	*CD0793-CD0792*
CD0792			Putative membrane protein, DUF81 family	0.39	0.43		**−**	
CD2636			Putative membrane protein	0.20	0.34	σ^G^?	**−**	*CD2636-CD2634*
CD2635			Putative membrane protein	0.33	0.5		**+**	
CD2634			Conserved hypothetical protein	0.27	0.65		**+**	
CD2315			Putative exported protein	0.31	0.38		**−**	
CD3551.1			Putative membrane protein	0.11	0.18	σ^G^	−	
CD2051			Putative membrane protein		0.52		**−**	
CD1789			Putative membrane protein, DUF421 family		0.61	σ^G^	**+**	*CD1789-CD1788*
CD1788			Conserved hypothetical protein		0.43		**−**	
CD2465			Putative amino acid/polyamine transporter	0.36	0.52	σ^G^	**+**	
**stress**								
CD1567	*cotG*		putative manganese catalase	0.25	0.27	σ^G^	**+**	
CD2845	*rbr*		Rubrerythrin	0.14	0.27	σ^G^	**+**	
CD1631	*sodA*		Superoxide dismutase (Mn)	0.06	0.32		**+**	
**miscellaneous**								
CD1486			Putative ribosome recycling factor	0.10	0.17	σ^G^?	**+**	
CD0684			ATP-dependent peptidase, M41 family	0.29	0.46	σ^G^	**+**	
CD1543			putative FMN-dependent NADH-azoreductase	0.16	0.43	σ^G^	**+**	
CD2431			Putative nitrite/sulphite reductase	0.23	0.54		**+**	
CD1707			Putative C4-dicarboxylate anaerobic carrier	0.29	0.53	σ^G^	**+**	
CD3489			Putative oligoendopeptidase F		0.62	σ^G^	**−**	
CD1676	*pcp*		Pyrrolidone-carboxylate peptidase		0.66		**+**	*CD1677-CD1676*
CD1677			Putative membrane protein	0.48	0.51		**−**	
CD2841			Putatie amidohydrolase		0.57	σ^G^	**+**	
**proteins of unknown function**								
CD0543			Conserved hypothetical protein	0.38	0.57	σ^G^	**−**	
CD1297			Conserved hypothetical protein	0.31	0.66		**−**	
CD1298			Conserved hypothetical protein	0.19	0.55		**−**	
CD1301.1			Conserved hypothetical protein	0.28	0.45		**−**	
CD1354			Conserved hypothetical protein	0.22	0.48	σ^G^	**−**	
CD1463			Conserved hypothetical protein	0.06	0.22	σ^G^	**+**	
CD1880			Conserved hypothetical protein	0.07	0.25	σ^G^	**+**	
CD2112			Conserved hypothetical protein	0.03	0.26	σ^G^	**+**	
CD2245.1			Conserved hypothetical protein	0.10	0.3	σ^G^	**+**	
CD2375			Conserved hypothetical protein	0.14	0.24	σ^G^	**+**	
CD2808			Conserved hypothetical protein	0.05	0.64	σ^G^	**−**	
CD2809			Conserved hypothetical protein	0.05	0.45	σ^G^?	**+**	
CD1568			Conserved hypothetical protein		0.60	σ^G^	**−**	
CD3610			Conserved hypothetical protein		0.66	σ^G^	**+**	

a A gene is considered differentially expressed when the *P* value is <0.05 using the statistical analysis described in Materials and Methods.

b We search for promoters recognized by the different sigma factors active in forespore upstream of the TSS mapped by RNA-seq. These promoters are listed in Table S6. 3 promoters with the −10 and/or −35 elements less conserved are indicated with a question mark.

c Proteins that are detected associated with the spore by a proteomic approach [39].

**Table 2:** The mother cell line expression with the identification of the σ^E^ and σ^K^ regulons and the regulation by SpoIIID of σ^E^-controlled genes.

Gene		Function	Expression ratio in transcriptome^a^		Promoter^b^	Detected in spore^c^	operon
Members of the σ^E^ regulon			*sigE*/630Δ*erm*	*spoIIID*/630 Δ*erm*			
**sporulation**							
*CD0124*	*spoIID*	peptidoglycane hydrolase SpoIID	0.24		σ^E^	**−**	
*CD1192*	*spoIIIAA*	ATP-binding stage III sporulation protein	0.05	3.85^d^	σ^E^	**−**	*spoIIIAA-spoIIIAH*
*CD1193*	*spoIIIAB*	Stage III sporulation protein AB	0.05	3.8		**−**	
*CD1194*	*spoIIIAC*	Stage III sporulation protein AC	0.03	4.9		**−**	
*CD1195*	*spoIIIAD*	Stage III sporulation protein AD	0.07	3.4		**−**	
*CD1196*	*spoIIIAE*	Stage III sporulation protein AE	0.09	2.4		**−**	
*CD1197*	*spoiIIIAF*	Stage III sporulation protein AF	0.08	3.6		**−**	
*CD1198*	*spoIIIAG*	Stage III sporulation protein AG	0.02	7.75^d^	σ^E^	**−**	
*CD1199*	*spoIIIAH*	Stage III sporulation ratchet engulfment protein	0.05	3.3		**−**	
*CD0126*	*spoIIID*	Transcriptional regulator SpoIIID	0.04		σ^E^	**−**	
*CD2629*	*spoIVA*	Assembly of cortex and coat layers	0.02	3.5	σ^E^	**+**	
*CD3567*	*sipL*	Functional homolog to SpoIVD	0.05	3	σ^E^	**+**	
*CD2443*		Conserved hypothetical protein YqfC-like	0.05		σ^E^	**+**	*CD2443-CD2442*
*CD2442*	*spoIV*	Stage IV sporulation protein YqfD-like	0.14			**−**	
*CD1230*	*sigK*	Fragment of sigma-K factor	0.13		σ^E^ and σ^K^	**−**	
*CD1234*		Putative phage protein, skin element	0.22		σ^E^	**−**	
*CD0106*	*cwlD*	N-acetylmuramoyl-L-alanine amidase	0.14		σ^E^	**−**	
*CD2247*	*cspBA*	serine germination protease	0.11		σ^E^	**+**	*cspAB-cspC*
*CD2246*	*cspC*	serine germination protease	0.16			**+**	
*CD3542*	*spmA*	Spore maturation protein A	0.16		σ^E^	**−**	*spmA-spmB*
*CD3541*	*spmB*	Spore maturation protein B	0.11	2		**−**	
*CD1511*	*cotB*	Spore outer coat layer protein CotB	0.05	3.5	σ^E^	**+**	
*CD0213*		Putative spore coat protein	0.42			**−**	*CD0214-CD0213*
*CD0214*		Conserved hypothetical protein	0.23			**+**	
*CD1320*		Putative peptidase, M16 family	0.25			**−**	*CD1320-CD1322*
*CD1321*		Putative sporulation protein	0.23			**−**	
*CD1322*	*dapG*	Aspartokinase 1	0.32			**−**	
*CD2641*		Putative sporulation protein	0.13		σ^E^	**−**	*CD2641-CD2639*
*CD2640*	*nrdR*	Transcriptional regulator, NrdR family	0.23			**−**	
*CD2639*		Putative cytotoxic factor	0.21			**−**	
*CD0782*		Putative sporulation protein YunB	0.32			**−**	
*CD1168*		YlbJ-like protein, spore cortex formation	0.18			**−**	
*CD3455*		C-terminal protease, homolog of CtpB	0.25		σ^E^	**−**	
*CD3494*		Putative spore protein	0.20		σ^E^	**−**	*CD3494-CD3493*
*CD3493*		Putative membrane protein	0.18			**−**	
**envelopes**							
*CD3464*		Conserved hypothetical protein	0.04		σ^E^	**−**	*CD3464-CD3463*
*CD3463*	*alr2*	Alanine racemase 2	0.05			**+**	
*CD2761*		N-acetylmuramoyl-L-alanine amidase	0.40			**−**	
*CD2833*		Putative calcium-transporting ATPase	0.22	2.25^d^	σ^E^	**−**	
*CD0760*		Putative Ca2+/Na+ antiporter	0.20		σ^E^	**−**	
*CD3483*		Putative zinc/iron permease	0.34		σ^E^	**−**	
*CD2445*		Transmembrane signaling protein, TspO	0.07	2.4	σ^E^	**−**	
*CD0131*		Putative membrane protein	0.18			**−**	
*CD0314*		Putative membrane protein	0.15		σ^E^	**−**	
*CD1301*		Putative membrane protein	0.33			**−**	
*CD1416*		Putative membrane protein	0.20	2.2	σ^E^	**−**	
*CD1928*		Putative membrane protein	0.14	3^d^		**−**	*CD1928-CD1929*
*CD1929*		Putative membrane protein	0.27			**−**	
*CD1940*		Putative membrane protein	0.08		σ^E^	**−**	
*CD2800*		Putative membrane protein	0.11	2.6	σ^E^	**−**	
*CD3636*		Putative membrane protein	0.42			**−**	*CD3636-CD3635*
*CD3635*		Conserved hypothetical protein	0.29			**−**	
**metabolism**							
*CD3638*		Conserved hypothetical protein	0.21		σ^E^	**−**	*CD3638-CD3637*
*CD3637*		NADPH-dependent FMN reductase	0.27			**−**	
*CD2429.1*		4Fe-4S ferredoxin	0.27		σ^E^	**−**	*CD2429.1-CD2428*
*CD2429*		Flavodoxin/ferredoxin oxidoreductase, alpha	0.31			**−**	
*CD2428*		Flavodoxin/ferredoxin oxidoreductase, beta	0.38			**−**	
*CD3251*		Putative dehydrogenase	0.25		σ^E^	**−**	
*CD3258*		Iron hydrogenase	0.08	2.3^d^	σ^E^	**−**	
*CD2000*	*isp*	Intracellular serine protease	0.18		σ^E^	**−**	
*CD3652*		Putative peptidase, M1 family	0.12			**+**	
*CD3521*		Putative peptidase T, M20B family	0.33		σ^E^	**+**	
*CD1085*		Putative membrane protein	0.26		σ^A^	**−**	*CD1085-CD1086*
*CD1086*		Putative peptidase, M20D family	0.26			**−**	
*CD1555*		Putative amino acid permease	0.22			**−**	
*CD1746*	*gltC*	Sodium/glutamate symporter	0.20		σ^E^	**−**	
*CD1259*	*brnQ-1*	Branched chain amino acid transporter	0.39			**−**	
*CD2439*		Diacylglycerol kinase/undecaprenol kinase	0.45			**+**	
*CD1068*		Polysaccharide biosynthesis protein	0.23			**−**	
*CD3257*		Polysaccharide deacetylase	0.11	2.1		**−**	
*CD3248*		Polysaccharide deacetylase	0.09	2.3^d^	σ^E^	**−**	
*CD1319*		Polysaccharide deacetylase	0.06	3.2^d^	σ^E^	**+**	
*CD0982*	*ubiA*	Putative UbiA prenyltransferase	0.39			**−**	
**miscellaneous**							
*CD3235*	*ssb*	Single-stranded DNA-binding protein	0.03	3.6^d^	σ^E^	**−**	
*CD1167*	*recV*	Tyrosine DNA recombinase, XerC/D family	0.26		σ^E^	**−**	
*CD2864*		Putative hydrolase	0.08	4	σ^E^	**−**	
*CD3298*		Putative ATP/GTP-binding protein	0.19		σ^E^	**−**	
*CD3462*	*mazE*	Putative antitoxin EndoAI	0.05			**−**	*CD3462-CD3461*
*CD3461*	*mazF*	Endoribonuclease toxin	0.11			**−**	
*CD2865*		Putative bacterioferritin	0.19	2.2^d^	σ^E^	**+**	
*CD0757*		Putative diguanylate kinase signaling protein	0.45			**−**	
*CD1616*		Putative diguanylate kinase signaling protein	0.37			**−**	
*CD2637*		Two-component sensor histidine kinase	0.30			**−**	
**proteins of unknown function**							
*CD0129*		Conserved hypothetical protein, DUF1256	0.15		σ^E^	**−**	
*CD2121*		Conserved hypothetical protein	0.23			**−**	
*CD0311*		Conserved hypothetical protein	0.02	4.1	σ^E^	**−**	
*CD2374*		Conserved hypothetical protein	0.23			**−**	
*CD1884*		Conserved hypothetical protein	0.11		σ^E^	**−**	
*CD1726*		Conserved hypothetical protein	0.05	3.9^d^	σ^E^	**−**	
*CD3457*		Conserved hypothetical protein	0.05	2.6	σ^E^	**−**	
*CD3465*		Conserved hypothetical protein	0.40			**−**	*CD3466-CD3465*
*CD1066*		Conserved hypothetical protein	0.16			**−**	
*CD3522*		Conserved hypothetical protein	0.09		σ^E^	**+**	
*CD2816*		Conserved hypothetical protein	0.11		σ^E^	**−**	
*CD3234*		Conserved hypothetical protein	0.09			**−**	
*CD1930*		Conserved hypothetical protein	0.27			**+**	
*CD1063*		Conserved hypothetical protein	0.11	3	σ^E^	**−**	

Members of the σ^K^ regulon			*sigE*/630 Δ*erm*	*sigK*/630 Δ*erm*			
**sporulation**							
*CD1613*	*cotA*	Spore outer coat layer protein CotA	0.10	0.05	σ^K^	**+**	
*CD0596*		Conserved hypothetical protein		0.02	σ^K^ ?^e^	**+**	*CD0596-cotCB*
*CD0597*	*cotF*	Spore coat peptide assembly protein		0.02		**+**	
*CD0598*	*cotCB*	Spore-coat protein CotCB manganese catalase	0.18	0.01		**+**	
*CD2399*		Conserved hypothetical protein	0.14	0.01	σ^K^ ?^e^	**+**	*CD2399-CD2401*
*CD2400*	*cotJB2*	Spore coat peptide assembly protein CotJB 2	0.10	0.01		**+**	
*CD2401*	*cotD*	Spore coat protein CotD manganese catalase	0.11	0.01		**+**	
*CD1433*	*cotE*	Spore coat protein CotE peroxiredoxin/chitinase	0.14	0.03	σ^K^	**+**	
*CD0551*	*sleC*	Spore cortex-lytic enzyme pre-pro-form	0.15	0.14	σ^K^	**+**	
*CD0332*	*bclA1*	Putative exosporium glycoprotein	0.21	0.08	σ^K^ ?^e^	**+**	
*CD3230*	*bclA2*	Putative exosporium glycoprotein	0.12	0.04	σ^K^ ?^e^	**−**	
*CD3349*	*bclA3*	Exosporium glycoprotein BclA3	0.01	0.02	σ^K^ ?^e^	**−**	
*CD2968*	*dpaA*	Dipicolinate synthase subunit A	0.30	0.08	σ^K^	**−**	*CD2968-CD2967*
*CD2967*	*dpaB*	Dipicolinate synthase subunit B	0.29	0.09		**−**	
*CD3569*		Sporulation-specific protease, YabG-like		0.22	σ^K^	**−**	
**envelopes**							
*CD0119*	*glmM*	Phosphoglucosamine mutase		0.49		**−**	
*CD0120*	*glmS*	Glucosamine-fructose-6-P aminotransferase		0.52	σ^K^/σ^A^	**−**	
*CD2664*	*murE*	UDP-N-acetylmuramyl-tripeptide synthetase	0.45	0.42	σ^K^/σ^A^	**−**	
*CD3007*		Putative L,D-transpeptidases	0.37	0.48		**+**	
*CD2184*		Putative N-acetylmuramoyl-L-alanine amidase	0.21	0.13	σ^K^	**−**	
*CD1898*		Putative N-acetylmuramoyl-L-alanine amidase		0.12	σ^K^	**+**	*CD1898-CD1897*
*CD1897*		Conserved hypothetical protein		0.13		**−**	
*CD0514*	*cwpV*	Cell surface protein	0.63	0.20		**+**	
*CD1518*	*feoA*	Ferrous iron transport protein		0.05	σ^K^	**−**	*CD1518-CD1517*
*CD1517*	*feoB*	Ferrous iron transport protein B		0.05		**−**	
*CD1904*		ABC-type transport system, permease	0.26	0.13		**−**	
*CD1927*	*ytlC*	ABC transporter component, ATP-binding	0.3	0.08	σ^K^	**−**	
*CD1891*		Fragment of ABC-type transport system	0.26	0.08	σ^K^	**−**	
*CD2720*		Putative transporter		0.16		**−**	
*CD0902*		Putative cation efflux protein	0.29	0.17	σ^K^	**−**	
*CD2144*		Putative membrane protein	0.46	0.12	σ^K^	**−**	
*CD2346*		Putative membrane protein		0.24		**−**	
*CD2459*		Putative glucokinase, ROK family		0.41	σ^K^	**−**	*CD2459-CD2458*
*CD2458*		Transporter, Major Facilitator Superfamily		0.39		**−**	
**miscellaneous**							
*CD3032*		Putative PLP-dependent transferase	0.32	0.11		**+**	
*CD0309*		Putative hydrolase, HAD superfamily		0.38		**−**	
*CD3350*		Putative glycosyl transferase, family 2		0.09		**−**	
*CD1951*		putative Acyl-CoA N-acyltransferase		0.19		**−**	
*CD0749*		Putative DNA helicase, UvrD/REP type	0.32	0.05		**−**	
*CD2537*		Membrane-associated 5′-nucleotidase/phosphoesterase	0.15	0.22		**−**	
*CD1845*		Membrane protein Tn1549	0.14	0.17		**−**	*CD1845-CD1846*
*CD1846*		Conjugative transposon protein Tn1549	0.15	0.20		**−**	
*CD0564*		Putative ATP-dependent protease		0.39	σ^K^	**−**	
**proteins of unknown function**							
*CD0196*		Fragment of conserved hypothetical protein	0.18	0.18		**−**	
*CD0896*		Conserved hypothetical protein	0.05	0.02	σ^K^	**−**	
*CD1063.1*		Conserved hypothetical protein	0.17	0.21	σ^K^	**+**	
*CD1063.2*		Conserved hypothetical protein	0.01	0.04	σ^K^	**−**	*CD1063.2-CD1063.3*
*CD1063.3*		Conserved hypothetical protein	0.01	0.04		**−**	
*CD1065*		Conserved hypothetical protein	0.06	0.30	σ^K^ ?^e^	**−**	
*CD1067*		Conserved hypothetical protein	0.00	0.03	σ^K^	**+**	
*CD1133*		Conserved hypothetical protein	0.12	0.16	σ^K^	**+**	
*CD1581*		Conserved hypothetical protein	0.01	0.01		**+**	
*CD2055*		Conserved hypothetical protein		0.33		**−**	
*CD2409*		Conserved hypothetical protein		0.11	σ^K^	**−**	
*CD3580*		Conserved hypothetical protein	0.11	0.12	σ^K^	**+**	
*CD3613*		Conserved hypothetical protein		0.04	σ^K^	**+**	
*CD3620*		Conserved hypothetical protein, similar to YmaF	0.34	0.04		**+**	

a A gene is considered differentially expressed when the *P* value is <0.05 using the statistical analysis described in Materials and Methods.

b we search for promoters recognized by the different sigma factors active in mother cell upstream of the TSS mapped by RNA-seq. These promoters are listed in Table S7.

c Proteins that are detected associated with the spore by a proteomic approach [39].

d Using the consensus sequence recognized by SpoIIID in *B. subtilis* [14], we identified a putative SpoIIID binding motif upstream of these genes.

e σ^K^? indicated the presence of sequences similar to σ^K^ consensus elements upstream of these genes but the TSS was not mapped (see Table S7).

#### The σ^F^ regulon

Among the 111 genes controlled by σ^F^ in transcriptome, 25 were downregulated in the *sigF* mutant but not in the *sigE*, *sigG* or *sigK* mutant (Table 1). We identified 10 σ^F^-dependent promoters controlling the expression of 14 genes. The σ^F^ regulon is the smallest of the four-compartment specific regulons as observed in *B. subtilis* 9,10. The orthologs of *B. subtilis* genes involved in sporulation regulatory functions, in spore morphogenesis or in germination [5], [16], [17], [20] were downregulated in the *sigF* mutant compared to strain 630Δerm. σ^F^ positively controlled the expression of *spoIIR*, encoding a signaling protein that in *B. subtilis* triggers the activation of the membrane bound SpoIIGA protease that in turn cleaves the pro-σ^E^ protein and leads to the production of an active σ^E^ factor [5]. The *spoIIQ* and *spoIIP* genes, the expression of which was also downregulated in the *sigF* mutant, are involved in the engulfment process. SpoIIP is a component of a molecular machine involved in dissolution of the septal cell wall during engulfment in *B. subtilis* [35], [36]. The *B. subtilis* SpoIIQ protein interacts with a mother cell-specific protein across the intermembrane space, an interaction that helps driving engulfment and that is also central to the formation of a channel linking the mother cell to the forespore [6], [37], [38]. In addition, σ^F^ was required to transcribe the *gpr* gene encoding a protease that is important for the degradation of small acid-soluble spore proteins (SASPs) during germination. The orthologs of these genes are members of the σ^F^ regulon in *B. subtilis* [9], [10], [16]. On the contrary, *gpr*, *spoIIR* and *spoIIP* are not controlled by σ^F^ in *C. acetobutylicum* [22]. σ^F^ was also required for the expression of genes involved in cell wall metabolism: *sleB*, *CD2141* and *CD1229* encoding a spore cortex hydrolysing protein, a DAla-DAla carboxypeptidase and a peptidoglycan (PG) glycosyltransferase, respectively. Moreover, σ^F^ positively controlled the expression of 5 genes encoding proteins previously detected in the spore proteome [39]: a glyceraldehyde-3P-dehydrogenase (CD0580), three peptidases (CD2660, CD2661 CD3595), and an RNA helicase (CD0761) (Table 1). Finally, 3 genes encoding proteins of unknown function and 2 genes encoding putative membrane proteins (*CD2686* and *CD2856*) were downregulated in the *sigF* mutant.

#### The σ^E^ regulon

We found 97 genes to be downregulated in a *sigE* mutant but not in a *sigG* mutant or in a *sigK* mutant (Table 2). TSS mapping allowed us to identify 47 σ^E^-dependent promoters controlling the expression of 63 genes (Table S7). σ^E^ controls the largest of the four cell type-specific sporulation regulons as also found for *B. subtilis* [9], [12]. Several σ^E^ target genes in *C. difficile* are orthologs of genes known to control engulfment, cortex formation, initiation of spore coat assembly, preparation of the late phase of sporulation or germination in *B. subtilis* (Table 2). This is consistent with the morphological block imposed by a *sigE* insertional mutation, which arrests development at the asymmetric division stage [19]. *spoIID*, which encodes a PG hydrolase required for the engulfment of the forespore by the mother cell was controlled by σ^E^. In *B. subtilis*, SpoIID is associated to SpoIIP and SpoIIM to form the DMP machine necessary for engulfment [35], [36]. The *spoIIP* gene was only controlled by σ^F^ in the transcriptome analysis. However, in qRT-PCR experiments, we showed that *spoIIP* expression decreased 10-fold in a *sigE* mutant and 800-fold in a *sigF* mutant. This suggested that this gene was under the control of both sigma factors as observed in *B. subtilis* 10,12. Also, even though a membrane protein (CD1221) sharing weak similarity to the N-terminal part of *B. subtilis* SpoIIM is present in the *C. difficile* genome, its gene was not differentially expressed between the 630Δerm strain and the *sigE* or *sigF* mutant in our conditions. In addition, σ^E^ activated the expression of the *spoIIIAA* octacistronic operon encoding an ATPase and membrane proteins that localize to the outer forespore membrane in *B. subtilis*, and integrate with SpoIIQ a novel type of secretion system [6], [37], [38].

In *B. subtilis*, the synthesis of the protective envelopes that encase the spore, the coat and the cortex PG, is initiated under σ^E^ control [12]. In *C. difficile*, the expression of *spoIVA* and *sipL* encoding an ortholog of the SpoIVA morphogenetic ATPase and a functional homolog to *B. subtilis* SpoVID was downregulated in the *sigE* mutant. These proteins are required for proper spore coat localization around the forespore in *C. difficile* [40]. The expression of *cotB* encoding a recently identified coat protein [41] was also specifically regulated by σ^E^. Accordingly, a σ^E^-dependent promoter was mapped upstream of *spoIVA*, *sipL* and *cotB* (Table S7). Moreover, an ortholog of the *cwlD* gene, encoding a N-acetylmuramoyl-L-alanine amidase required for the synthesis of muramic ∂-lactam, a specific cortex PG compound [42], was controlled by σ^E^. On the contrary, the second enzyme of this pathway, the PdaA deacetylase, was produced in the forespore (see below). The synthesis of other polysaccharide deacetylases (CD3257, CD3248, CD1319), of a polysaccharide biosynthetic enzyme (CD1068), of an alanine racemase (Alr2) and of a N-acetylmuramoyl-L-alanine amidase (CD2761) was also under σ^E^ control. *CD1168* encoding an YlbJ-like protein, and the *CD2443-CD2442* operon encoding YqfC and YqfD-like proteins were controlled by σ^E^. The inactivation of *yqfC*, *yqfD* and *ylbJ* in *B. subtilis* reduces heat resistance of the spore and prevents the development of refractility, both phenotypes attributed to defects in cortex formation [12], [13]. In addition, the expression of the *spmA*-*spmB* operon involved in spore core dehydratation and heat resistance in *B. subtilis* and *C. perfringens* [43] was reduced in the *sigE* mutant. We observed a positive control by σ^E^ of the synthesis of CD2865, an homolog of a bacterioferritin involved in iron storage and oxidative stress protection in the anaerobe *Desulfovibrio vulgaris* [44].

Several genes under σ^E^ control in *B. subtilis* are required for spore germination because they are involved in modifications of the cortex or in coat assembly. In *C. difficile*, σ^E^ was required for the transcription of the *csp* operon encoding serine proteases. In *C. perfringens* and *C. difficile*, CspB is necessary for pro-SleC maturation to form the spore cortex lytic enzyme SleC during germination [45], [46], [47] while CspC is a germinant receptor for taurocholate [48]. The promoter of the *C. difficile csp* operon matched the consensus for σ^E^-dependent promoters while *sleC* was transcribed from a σ^K^-dependent promoter (see below). We also observed a positive control by σ^E^ of three other proteases (CD2000, CD3455, CD1320). CD3455 shares 38% identity with CtpB, a protease required for σ^K^ activation in *B. subtilis* [6] and the Isp ortholog of CD2000, is involved in sporulation in *Bacillus thuringiensis* [49]. Presumably, these proteases also participate in the spore formation process in *C. difficile*.

Another major function under σ^E^ control in *B. subtilis* is the maintenance of a sufficient level of metabolic activity to enable the completion of sporulation [9], [12]. σ^E^ also controlled the expression of several metabolic genes in *C. difficile* (Table 2). Among them are genes encoding proteins likely to be required for oxidoreduction pathways and energy metabolism: an oxido-reductase (CD2429.1-CD2429-CD2428), a FMN reductase (CD3637), a dehydrogenase (CD3251) and an iron hydrogenase (CD3258). In addition, σ^E^ activated the expression of genes encoding peptidases (CD3652, CD1086, CD3521) or amino acids permeases (CD1746, CD1555 and CD1259). Moreover, the *CD2833* and *CD0760* genes encoding a Ca^2+^-transporting ATPase and a Ca^2+^/Na^+^ antiporter, respectively, were downregulated in the *sigE* mutant. These proteins are probably important for the import or export of Ca^2+^, a key element of the sporulation and germination processes in endospore forming Firmicutes [47].

Other *C. difficile* σ^E^ controlled genes do not belong to any of the categories above. Of particular interest, σ^E^ controlled the expression of the *mazF-mazE* operon, encoding the unique toxin-antitoxin (TA) system of *C. difficile* [50]. In *B. subtilis*, a different TA system, SpoIISA-SpoIISB synthesized in the mother cell, is involved in sporulation [51]. MazF cleaves mRNA while the SpoIISA toxin is targeted to the cell membrane. The role of MazEF in the sporulation process remains to be characterized. Finally, 18 genes encoding proteins of unknown function were downregulated in the *sigE* mutant compared to the 630Δerm strain. Only 15 members of the σ^E^ regulon are detected in the spore proteome [39]. This included the SpoIVA and SipL proteins, the CotB coat protein, the Csp-like proteases, the YqfC like protein and three proteins of unknown function (CD0214, CD3522 and CD1930).

#### The σ^G^ regulon

From the transcriptome experiments, we identified 50 genes controlled by σ^G^ but not by σ^K^ (Table 1). The TSS mapping performed by RNA-seq allowed us to identify 30 σ^G^-dependent promoters controlling the expression of 33 genes. Several σ^G^-controlled genes encode proteins sharing similarities with proteins involved in spore resistance in *B. subtilis* [5], [16], [52]. Dipicolinic acid (DPA) is a major spore component important for spore resistance. The expression of the *spoVA* operon whose products are required for the import of DPA into the forespore from the mother cell [53], decreased in a *sigG* mutant (Table 1). A *C. perfringens spoVA* mutation prevents the accumulation of DPA and reduces spore viability [47]. As observed in *B. subtilis*, we also showed the requirement for σ^G^ for the expression of all the genes encoding SASPs; these proteins bind to the forespore chromosome protecting the DNA from damage [52]. Indeed, the *sspA* (*CD2688*) and *sspB* (*CD3249*) genes encoding alpha/beta-type SASP and two other genes annotated as SASPs (*CD1290* and *CD3220.1*) were expressed under the direct σ^G^ control (Table 1). Moreover, *CD0684* encoding an ATP-dependent protease sharing similarity with FtsH, a protein involved in protein quality control and stress resistance in eubacteria, was downregulated in a *sigG* mutant. In addition, genes encoding proteins presumably involved in mitigating oxidative stress were also controlled by σ^G^. *CD1567* (*cotG*), *CD1631* (*sodA*) and *CD2845* encode a catalase, a superoxide dismutase and a rubrerythrin, respectively. Interestingly, SodA and CotG has recently been detected at the *C. difficile* spore surface [54]. These results suggested that proteins synthesized under the control of σ^G^ are located to the spore surface in *C. difficile*.

Expression of several genes required for cell wall synthesis and cortex formation was controlled by σ^G^. Thus, expression of *CD1430* (*pdaA*) encoding a N-acetylmuramic acid deacetylase involved in the formation of muramic ∂-lactam in the spore cortex PG and indirectly required for efficient spore germination [42] was downregulated in a *sigG* mutant. In addition, *dacF*, encoding a D-alanyl-D-alanine carboxypeptidase regulating the degree of cross-linking of the spore PG as well as *CD0784* and *uppS* coding for an amidase and an undecaprenyl pyrophosphate synthetase, respectively were also positively controlled by σ^G^.

Finally, the transcriptome analysis revealed 16 σ^G^-controlled genes coding for proteins of unknown function. One of the most important aspects of spore biology is the mechanism by which dormant spores sense a suitable environment for germination and trigger the process through the specific recognition of germinants by germination receptors. These receptors are located in the spore inner membrane and are synthesized in the forespore under σ^G^ control in *B. subtilis* [9], [10]. No known homologs of the *B. subtilis gerA*, *gerB* and *gerK* operons as well as of the *gerA*, *gerKB*, *gerKA* and *gerKC* genes of *C. perfringens* are found in the *C. difficile* genome [55], [56]. However, a bile acid germinant receptor CspC has been recently identified [48]. Nine genes encoding probable membrane proteins (CD0792, CD0793, CD1677, CD1789, CD2051, CD2465, CD2635, CD2636, CD3551.1) were downregulated in the *sigG* mutant and these proteins may be involved in germination. 55% of our σ^G^-controlled genes encode proteins associated to the spore in proteome [39]. This is in agreement with the crucial role of σ^G^ in the synthesis of key components protecting the spore or in preparation for germination.

#### The σ^K^ regulon

In transcriptome analysis, we found 56 genes positively controlled by σ^K^ (Table 2). The TSS mapping allowed us to identify 24 σ^K^-dependent promoters controlling the expression of 29 genes. In *B. subtilis*, σ^K^ plays a crucial role in the last steps of the spore coat assembly [5], [57]. While the *B. subtilis* spore coat comprises over 70 different proteins, only few of them are conserved in *C. difficile* [57]. Recent studies have identified eight components of the *C. difficile* spore coat, CotA, CotB, CotCB, CotD, CotE, CotF, CotG and SodA [41], [54]. CotCB and CotD are catalases while CotE is a bifunctional protein with peroxiredoxin and chitinase activities [41], [54]. Interestingly, the expression of the *cotA* and *cotE* genes as well as the *CD0596*-*cotF*-*cotCB* and *CD2399*-*cotJB2*-*cotD* operons was downregulated in a *sigK* mutant compared to strain 630Δerm. We mapped a σ^K^-dependent promoter upstream of *cotA* and *cotE* while σ^K^ consensus elements were found upstream of *CD0596* and *CD2399* (Table S7). The expression of *CD3569* encoding a YabG-like protease involved in post-translational modification of spore surface proteins in *B. subtilis* [57] was also positively controlled by σ^K^. Three orthologs of the *B. anthracis bclA* gene (*bclA1*, *bclA2* and *bclA3*) are present in *C. difficile*. BclA of *B. anthracis* is a collagen-like protein that forms the hair-like nap of the exosporium, and is the immunodominant antigen of the spore surface [58]. The *bclA1*, *bclA2* and *bclA3* genes were positively controlled by σ^K^ and sequences similar to promoters recognized by σ^K^ are found upstream of these genes (Table S7).

An important conserved function of the mother cell is DPA production. The expression of *dpaA* and *dpaB* encoding the dipicolinate synthase was controlled by σ^K^. This operon was transcribed from a σ^K^-dependent promoter as observed in *B. subtilis* [9]. DPA is then transported into the spore by the SpoVA transporter, which is synthesized in the forespore. Mother cell lysis is a late developmental event that in *B. subtilis* involves the σ^K^-controlled production of the CwlC and CwlH N-acetylmuramic acid L-alanine amidases [14], [59]. Two genes encoding N-acetylmuramic acid L-alanine amidases (*CD1898* and *CD2184*) were downregulated in a *sigK* mutant and are possibly involved in mother cell lysis. We also found that *sleC* encoding the major spore cortex lytic enzyme that is essential for *C. difficile* germination [60] was a member of the σ^K^ regulon.

Other genes controlled by σ^K^ have no counterparts in *B. subtilis*. *CD0564* encodes a putative Lon-type protease. The late production of CD0564 from a σ^K^ promoter (Table 2) may play a role in the degradation of early mother cell-specific proteins such as σ^E^ or the MazE antitoxin whose ortholog in *E. coli* is degraded by ClpXP and Lon proteases [61]. If so, proteolysis of MazE may lead to the accumulation of active MazF, which may possibly participate in mother cell death. Finally, the transcriptome analysis showed that genes encoding 17 proteins of unknown function were downregulated in a *sigK* mutant. Interestingly, nine of these proteins are associated to the spore [39] and we mapped a σ^K^-dependent promoter upstream five of their encoding genes (*CD1063.1*, *CD1067*, *CD1133*, *CD3580* and *CD3613*). These proteins are most likely spore coat proteins but further work would be necessary to characterize their localization and their function in *C. difficile*. In conclusion, 21 out of the 57 genes controlled by σ^K^ were found to encode components of the spore proteome [39]. The overall composition of the σ^K^ regulon is in agreement with the crucial role of σ^K^ in the assembly of the spore surface layers, spore maturation and mother cell lysis in *C. difficile* as well as in other endospore forming Firmicutes [19], [57].

### III. The forespore line of gene expression

Our global approaches allowed to obtain new insights into the forespore line of gene expression, which is governed by the RNA polymerase sigma factors, σ^F^ and σ^G^, and the DNA-binding transcriptional regulator, SpoVT.

#### Control of *sigF* and *sigG* expression

The *spoIIAA-spoIIAB-sigF*operon, which is responsible for the synthesis and the activation/inactivation of σ^F^, is transcribed by the RNA polymerase σ^H^ holoenzyme and is positively controlled by Spo0A [27]. Like in *B. subtilis* and other spore formers, the *spoIIGA*, *sigE* and *sigG* genes are clustered in *C. difficile* [16], [20]. The *B. subtilis sigG* gene is transcribed from two promoters, one located upstream of *spoIIGA* (σ^A^) and the second located upstream of *sigG* transcribed first by σ^F^ and later by σ^G^ after engulfment [5]. In both *B. subtilis* and *C. acetobutylicum*, three transcripts are detected for the *spoIIGA*-*sigE*-*sigG* locus, corresponding to a *spoIIGA-sigE-sigG*, a *spoIIGA-sigE* and a *sigG* transcript [5], [33], [62]. In *C. difficile*, a σ^A^-dependent promoter (GTGACA and TATAAT boxes) was mapped by RNA-seq upstream of *spoIIGA* (TSS at position 3052270 in the genome of strain 630). A promoter was also mapped upstream of *sigG* both by RNA-seq (Table S6) and by 5′-RACE [27]. The associated consensus motifs clearly correspond to those found for the forespore sigma factors (Fig. 1A) but appear closer to that found for σ^F^ promoters as the −10 element contains a G at position 23 and lacks the A conserved at position 32 for σ^G^-dependent promoters (Fig. 1A). However, *sigG* expression was not downregulated in the *sigF* mutant in the transcriptome analysis as well as by qRT-PCR, under our conditions. In *B. subtilis*, *sigG* expression also appears not to be regulated in transcriptome analyses comparing *sigF* ^+^ and *sigF* ^−^ strains [9], [10]. By contrast, *sigG* expression is downregulated in a *sigF* mutant in *C. acetobutylicum* [22] suggesting some differences in the control of *sigG* expression. In *C. difficile*, the absence of control of *sigG* expression by σ^F^ strongly suggests that *sigG* is transcribed from at least two promoters, one in front of *spoIIGA* recognized by σ^A^, and one just upstream of *sigG* recognized by σ^F^ and/or by σ^G^. Studies with a P*sigG*-SNAP^Cd^ transcriptional fusion indicate that this second promoter responsible for the forespore-specific transcription of *sigG* is dependent on σ^F^ [19]. A more complete analysis of the fine tuning of *spoIIGA-sigE*-*sigG* locus expression will require further investigations.

It is worth noting that the expression of many *sigG*-controlled genes (82% of σ^G^ regulon members) including those for key sporulation proteins like SpoVA, SspA, SspB and PdaA was strongly reduced in a *sigF* mutant in transcriptome (Table 1). The absence of detection of the complete σ^G^ regulon in the *sigF* mutant might be due to the timing of sampling (14 h) that is probably not optimal for *sigG* targets. Moreover, inactivation of *sigF* also eliminated fluorescence from a P*sspA*-SNAP^Cd^ fusion [19]. Thus, σ^F^ directly or indirectly controls expression of the σ^G^ regulon. One possibility is that synthesis of the σ^G^ protein is abolished in a *sigF* mutant as observed in *C. acetobutylicum* and *C. perfringens* [22], [23]. Alternatively, the positive regulation of σ^G^ target genes by σ^F^ could be mediated through modulation of σ^G^ activity. In *B. subtilis*, for instance, two anti-sigma factors, SpoIIAB and CsfB (Gin) negatively regulate σ^G^ activity. CsfB, in particular, plays an important role in σ^G^ regulation in the forespore, and is produced under σ^F^ control [63], [64]. No CsfB ortholog is present in the *C. difficile* genome and the phenotype expected if σ^F^ would be required for the synthesis of an anti-σ^G^ factor would be the opposite. However, we cannot exclude that proteins synthesized under σ^F^ control might be necessary for σ^G^ activity. Yet another possibility is that both σ^F^ and σ^G^ were involved in the transcription of most σ^G^-target genes. In *B. subtilis*, σ^F^ and σ^G^ have overlapping promoter specificities and some genes are under the dual control of these sigma factors [9], [10]. This could be also the case in *C. difficile*. Finally, σ^F^ might control σ^G^ targets through an as yet unknown regulatory factor.

#### The role of SpoVT in sporulation

In *B. subtilis*, two transcriptional regulators participate in the forespore regulatory network, RfsA and SpoVT. SpoVT controls the synthesis of about half of the members of the σ^G^ regulon [10]. RfsA is absent from the genome of *C. difficile* and several Clostridia while an ortholog of SpoVT (55% identity with SpoVT of *B. subtilis*) is present. Interestingly, we found that *spoVT* expression was controlled by both σ^F^ and σ^G^ in transcriptome (Table 1). We confirmed by qRT-PCR a 280- and a 14-fold decrease of *spoVT* transcription in a *sigF* or in a *sigG* mutant compared to strain 630Δerm, respectively (Table 3). We also showed that the expression of *spoVT* was restored to the wild-type level in the *sigG* mutant complemented by *sigG* while its expression was 19-fold higher in the *sigF* mutant complemented by *sigF* compared to the wild-type strain (Table S9). Upstream of the *spoVT* TSS, we identified a promoter resembling both the σ^F^ and σ^G^ consensus elements with the presence of a G at position 23 and an A at position 32 (Table S6). So, *spoVT* might be a direct target of both σ^F^ and σ^G^ because contrary to other σ^G^-controlled genes, σ^F^ inactivation caused a much more important decrease of *spoVT* expression than σ^G^ inactivation.

**Table 3:** Control of σ^G^ or σ^K^ target genes by both σ^F^ and σ^E^.

Gene		Function	Expression ratio		
Forespore σ^G^-dependent			*sigF*/630Δerm	*sigE*/630Δerm	*sigG*/630Δerm
**transcriptome**					
*CD1486*		Putative ribosome recycling factor	0.10	0.26	0.17
*CD1631*	*sodA*	Superoxide dismutase (Mn)	0.06	0.21	0.32
*CD1880*		Conserved hypothetical protein	0.07	0.21	0.25
*CD3249*	*sspB*	Small, acid-soluble spore protein beta	0.01	0.04	0.07
*CD3551.1*		Putative membrane protein	0.11	0.31	0.18
**qRT-PCR**					
*CD0773*	*spoVAC*	DPA uptake protein, SpoVAC	<0.01	0.06	0.02
*CD0792*		Putative membrane protein, DUF81 family	<0.01	0.02	0.01
*CD1213*		Stage IVB sporulation protein B, peptidase	0.04	0.08	0.12
*CD1290*		Putative small acid-soluble spore protein	<0.01	0.05	0.01
*CD1430*	*pdaA*	Putative d-lactam-biosynthetic deacteylase	<0.01	0.14	0.03
*CD2375*		Conserved hypothetical protein	0.03	0.24	0.05
*CD2465*		Putative amino acid/polyamine transporter	0.18	0.15	0.19
*CD2636*		Putative membrane protein	<0.01	0.13	0.03
*CD2688*	*sspA*	Small, acid-soluble spore protein alpha	<0.01	0.05	0.02
*CD3499*	*spoVT*	Transcriptional regulator, SpoVT	<0.01	0.06	0.07

Mother cell σ^K^-dependent			*sigF*/630Δerm	*sigE*/630Δerm	*sigK*/630Δerm
**transcriptome**					
*CD0332*	*bclA1*	Putative exosporium glycoprotein	0.43	0.21	0.08
*CD0598*	*cotCB*	Spore-coat CotCB manganese catalase	0.18	0.18	0.01
*CD0896*		Conserved hypothetical protein	0.21	0.05	0.02
*CD1067*		Conserved hypothetical protein	0.07	0.00	0.03
*CD1581*		Conserved hypothetical protein	0.03	0.01	0.01
*CD1613*	*cotA*	Spore outer coat layer protein CotA	0.13	0.10	0.05
*CD2664*	*murE*	UDP-N-acetylmuramyl-tripeptide synthetase	0.51	0.45	0.42
*CD3230*	*bclA2*	Putative exosporium glycoprotein	0.16	0.12	0.04
*CD3349*	*bclA3*	Exosporium glycoprotein BclA3	0.12	0.01	0.02
*CD3620*		Conserved hypothetical protein	0.35	0.34	0.04
**qRT-PCR**					
*CD0551*	*sleC*	Spore cortex-lyic enzyme pre-pro-form	0.34	<0.01	0.01
*CD1133*		Conserved hypothetical protein	0.40	0.02	0.10
*CD1433*	*cotE*	Spore coat protein CotE peroxiredoxin/chitinase	0.32	0.01	0.01
*CD2401*	*cotD*	Spore coat CotD manganese catalase	0.24	0.01	<0.01

A gene is considered differentially expressed when the *P* value is <0.05 using the statistical analysis described in Materials and Methods.

qRT-PCR were performed as described in Materials and Methods.

**Figure 2:**
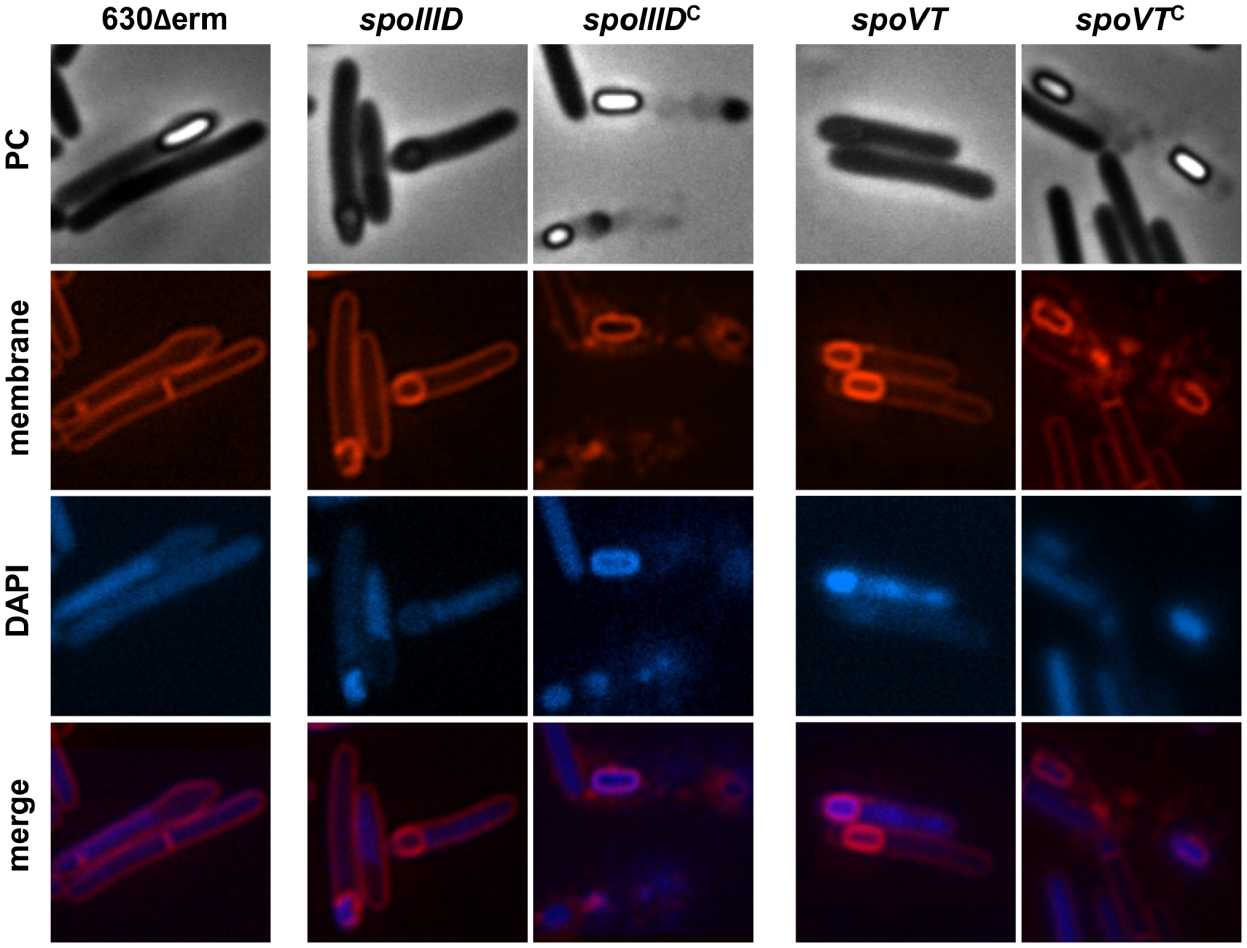
Morphological characterization of the *spoIIID* and *spoVT* mutants. Cells of the *C. difficile* 630Δerm strain, the *spoIIID* or *spoVT* mutant and the complemented strains, CDIP262, carrying a multicopy *spoIIID* gene controlled by its native promoter or CDIP263, carrying a multicopy *spoVT* gene controlled by its native promoter were collected after 24 h of growth in SM broth, stained with the DNA stain DAPI and the membrane dye FM4-64 and examined by phase contrast and fluorescence microscopy.

To test the possible role of SpoVT in sporulation in *C. difficile*, we constructed a *spoVT* mutant (Fig. S2). A complemented strain, CDIP263 carrying a multicopy allele of *spoVT* under the control of its native promoter was also obtained. To determine the impact of SpoVT inactivation on spore morphogenesis, samples of the strain 630Δerm, of the *spoVT* mutant and of the complemented strain CDIP263 (*spoVT*::*erm*, pMTL84121-*spoVT*) were collected and labeled with the DNA stain DAPI and the lipophilic membrane dye FM4-64. Phase contrast and fluorescence microscopy experiments showed that the *spoVT* mutant was able to complete the engulfment process (Fig. 2). However, this mutant formed phase dark immature spores, which were not released from the sporangial cells, clearly suggesting that the cortex is absent or highly reduced. The wild-type phenotype was restored in the complemented strain CDIP263. We further tested the ability of this mutant to sporulate. After 72 h of growth in SM medium, the 630Δerm strain or the *spoVT* mutant containing the plasmid pMTL84121-*spoVT* produced 4×10^6^ and 10^7^ heat-resistant spores/ml, respectively. In contrast, no clones were obtained after an heat treatment for the *spoVT* mutant. This was probably due to the lack of production of heat-resistant spores as suggested by the phase dark spores phenotype observed for this mutant (Fig. 2). In conclusion, SpoVT is required for mature spore formation in *C. difficile* but the phenotype of the *spoVT* mutant of *C. difficile* differs from that of the same mutant of *B. subtilis*. Indeed, the *spoVT* mutant of *C. difficile* formed phase dark spores instead of phase bright spores [65] and the *spoVT* mutant of *B. subtilis* has a reduced ability to sporulate [65] while we were unable to detect heat resistant spores for the mutant of *C. difficile*.

Then, we used qRT-PCR to measure the impact of SpoVT inactivation on the expression of selected σ^G^ targets after 20 h of growth. We showed that the expression of *sspA* and *sspB* decreased 70- and 12-fold, respectively in the *spoVT* mutant compared to strain 630Δerm (Table S11). A positive effect of SpoVT on *sspA* and *sspB* expression is also observed in *B. subtilis* [65], [66]. The decreased expression of the *sspA* and *sspB* genes is probably not sufficient to explain the phenotypes observed for the *spoVT* mutant but we did not see a reproducible differential expression for other tested genes in our conditions. However, the expression of *spoIIR* and *gpr*, two members of the σ^F^ regulon, was upregulated 4- and 9-fold in a *spoVT* mutant compared to strain 630Δerm earlier during sporulation (15 h) (Table S11). This negative effect might be due either to a direct control by SpoVT playing the role of RfsA in the negative control of σ^F^ targets or to an indirect effect associated with the blocked sporulation in the *spoVT* mutant.

No other genes encoding transcriptional regulators or proteins with DNA binding motifs were regulated by σ^F^ or σ^G^ in our transcriptome study (Table 1). Even though we cannot exclude that other regulatory proteins are at play, SpoVT is probably the key, if not the only, ancillary regulator of forespore gene expression. Further work will be required to analyse more precisely the morphology of the *spoVT* mutant and to understand at the molecular level the complex role of SpoVT in the regulatory network controlling sporulation.

### IV. The mother cell line of gene expression

As discussed above, *sigE* transcription is probably initiated at the σ^A^-dependent promoter located upstream of *spoIIGA*, which forms an operon with *sigE* and maybe also with *sigG*. In *B. subtilis*, σ^E^ positively controls the expression of *spoIIID* and *gerR* encoding regulatory proteins. GerR acts as a repressor of certain early σ^E^-controlled genes and as an activator of some σ^K^-dependent genes [67]. SpoIIID is an ambivalent regulator, acting as a repressor as well as an activator of a late class of σ^E^-controlled genes [14]. SpoIIID and σ^E^ jointly activate the expression of *sigK*, and σ^K^ in turn drives production of another ancillary regulator, GerE [14]. In *C. difficile*, the SpoIIID regulator is present while GerR and GerE are absent. The SpoIIID protein of *C. difficile* shares 64% identity with SpoIIID from *B. subtilis* with conservation of the HTH motif and of the basic DNA binding motif located near the C-ter of the protein (Fig. S3) [68].

#### Characterization of the SpoIIID regulon

The expression of *spoIIID* was strongly curtailed in a *sigE* mutant (25- and 800-fold in transcriptome and qRT-PCR) whereas the *spoIIID* expression was restored when a plasmid pMTL84121 containing the *sigE* gene with its promoter was introduced into the *sigE* mutant (Table S9). A TSS corresponding to a σ^E^-controlled promoter (ATA-N_16_-CATATATA) was mapped upstream of *spoIIID* as observed in *B. subtilis*. It is interesting to note that in *C. perfringens* the expression of *spoIIID* is not controlled by σ^E^ [21].

In *B. subtilis*, SpoIIID positively or negatively regulates almost half of the σ^E^ target genes [14]. To test the possible role of SpoIIID in the mother cell regulatory network in *C. difficile*, we constructed a *spoIIID* mutant (Fig. S2) and compared the expression profiles of the 630Δerm strain and of the *spoIIID* mutant after 15 h of growth in SM medium. We found 96 genes differentially expressed with a p value <0.05 between the 630Δerm strain and the *spoIIID* mutant (Table S10). 12 and 84 genes were down and upregulated in the *spoIIID* mutant, respectively. We then performed qRT-PCR on a subset of genes regulated by SpoIIID in our transcriptome. The qRT-PCR results confirmed the microarrays data for the tested genes (Table S5). First, 47 genes that are not under the control of sporulation sigma factors in our transcriptome analyses were regulated by SpoIIID either positivelly (10 genes) or negativelly (37 genes) (Table S10). Most of these genes encode proteins involved in metabolism. Among the genes under the negative control of *spoIIID*, 28 were *bona fide* members of the σ^E^-regulon (Table 2). Therefore, a principal function of SpoIIID is to inhibit the transcription of about 30% of the genes transcribed by σ^E^ as observed in *B. subtilis* [14]. SpoIIID repressed the expression of the *spoIIIA* operon and of the *spoIVA*, *sipL*, *cotB* and *spm* genes. In *B. subtilis*, *spoIIIAA* and *spoIVA* are direct SpoIIID targets [14]. By contrast, the *spoIID* gene, which is a direct SpoIIID target in *B. subtilis*, was not regulated by SpoIIID in transcriptome and qRT-PCR experiments in our conditions. In addition, the expression of genes encoding a transmembrane signaling protein (CD2445), an iron hydrogenase (CD3258), a bacterioferritin (CD2865), three polysaccharide deacetylases (CD3257, CD3248 and CD1319) and of several genes of unknown function was positively controlled by σ^E^ and negativelly regulated by SpoIIID (Table 2). By using the consensus sequence recognized by SpoIIID in *B. subtilis* [14], we identified a putative SpoIIID binding motif upstream of 11 of these genes (Table 2) suggesting that they might be direct SpoIIID targets in *C. difficile*. However, the characterization of direct and indirect SpoIIID targets and the experimental identification of the DNA binding motif of SpoIIID will deserve further investigations.

Interestingly, SpoIIID was also an activator of the expression of two genes belonging to the σ^K^ regulon, *bclA3* and *CD1067* in our transcriptome experiment (Table S10). To determine if *sigK* itself and other members of the σ^K^ regulon were under SpoIIID control, we performed qRT-PCR experiments (Table S11) using RNA extracted after 15 h of growth or after 24 h, a time where the σ^K^ targets are more highly expressed. We found that *sigK* expression was 25-fold and 250-fold downregulated in the *spoIIID* mutant after 15 h and 24 h of growth, respectively. This clearly indicated that the expression of *sigK* was positively controlled by SpoIIID in *C. difficile* as observed in *B. subtilis* [14]. In *B. subtilis*, SpoIIID is also required for the expression of *spoIVCA* encoding the site-specific recombinase involved in the excision of the *skin* element that creates a functional *sigK* gene [69]. In contrast, we did not observe a control of *CD1231* encoding the specific recombinase of the *skin* element by SpoIIID but also by SigE both in transcriptome and qRT-PCR experiments. This result strongly suggests a different mechanism of control for the *skin* excision in *C. difficile* compared to *B. subtilis*. We also showed that eleven *bona fide* σ^K^ targets such as *cotA*, *cotCB*, *cotD*, *cotE*, *sleC*, *bclA1*, *bclA2*, *bclA3*, *CD3580*, *CD1067* and *CD1133* were downregulated 30- to 1000-fold in the *spoIIID* mutant as compared to the parental strain after 24 h of growth (Table S11). The positive regulation of members of the σ^K^ regulon by SpoIIID might be due either to a direct binding of this regulator to their promoter regions or to an indirect effect mediated through the control of *sigK* transcription by SpoIIID.

To investigate the role of SpoIIID in sporulation, we examined the morphology of the *spoIIID* mutant. Samples of the 630Δerm strain, of the *spoIIID* mutant and of the complemented strain CDIP262 carrying a multicopy allele of *spoIIID* under the control of its native promoter (*spoIIID*::*erm*, pMTL84121-*spoIIID*) were collected and labeled with the DNA stain DAPI and the lipophilic membrane dye FM4-64. Phase contrast and fluorescence microscopy experiments showed that the *spoIIID* mutant completed engulfment of the forespore by the mother cell and formed partially refractile spores with irregular shapes and positioning (Fig. 2). The wild-type phenotype with the production of free spores was restored in the strain CDIP262 containing a copy of *spoIIID* on a plasmid (Fig. 2). We also tested the ability of the *spoIIID* mutant to sporulate. After 72 h of growth in SM medium, the 630Δerm strain produced 9×10^5^ heat-resistant spores/ml while a titer of 3×10^2^ heat-resistant spores/ml was obtained for the *spoIIID* mutant. When we complemented the *spoIIID* mutant with a pMTL84121-*spoIIID* plasmid, the titer of heat resistant spores increased to 5×10^5^/ml. Interestingly, both the morphology and the sporulation efficiency of the *spoIIID* mutant is reminiscent of the oligosporogenous phenotype obtained for the *sigK* mutant of *C. difficile* [19] in agreement with the dependency on SpoIIID of the transcription of *sigK*. The morphology and the sporulation efficiency of the *spoIIID* mutant of *C. difficile* are similar to those of the *B. subtilis* mutant [70], [71]. As demonstrated in *B. subtilis* [14], SpoIIID in *C. difficile* plays a pivotal role in the mother cell line of gene expression switching off the transcription of many members of the σ^E^ regulon and switching on the expression of *sigK* and of members of the σ^K^ regulon.

#### Control of *sigK* transcription

In addition to the positive control of *sigK* expression by SpoIIID, its expression also decreased 8- and 600-fold in a *sigE* mutant, in transcriptome and qRT-PCR experiments, respectively. The introduction of a plasmid pMTL84121 containing the *sigE* gene with its promoter into the *sigE* mutant restored *sigK* expression (Table S9). Interestingly, we mapped two TSS, 67 nt (P1) and 26 nt (P2) upstream of the translational start site of *sigK* (Table S7). A canonical −10 box (CATATTAT) for mother cell sigma factors is located upstream of the TSS corresponding to P1 while either a TTA sequence 15 bp upstream from the −10 box or a TTT motif with a more classical 16–18 bp spacing between the −10 and −35 elements could be proposed for a −35 motif. So, this promoter resembles a σ^E^-dependent promoter (Fig. 1B and Table S7). Upstream of the second TSS (P2), we found a consensus for σ^K^-dependent promoters with a −10 box (CATATAAT) and a −35 box (AC) (Table S7). We note that in *B. subtilis*, following the initial transcription of *sigK* under the command of σ^E^ and SpoIIID, an autoregulatory loop is established, which is responsible for about 60% of *sigK* transcription [72]. In *C. difficile*, *sigK* is likely first transcribed by σ^E^-associated RNA polymerase at P1 and then by σ^K^-associated RNA polymerase at P2 probably later during sporulation. In any event, lending support to the idea that σ^E^ has a crucial role in *sigK* transcription, 65% of the σ^K^-controlled genes were positively controlled by σ^E^ including genes encoding key components of the spore surface layers (*cotCB*, *cotA*, *cotE*, *cotD*, *sleC*, *bclA1*, *bclA2*, *bclA3*). Most of these genes were much more downregulated in the *sigK* mutant than in the *sigE* mutant (Table 2) suggesting that the timing of sampling of the *sigE* mutant (14 h) is probably not optimal for σ^K^ targets. This might explain the absence of detection of the complete σ^K^ regulon in the *sigE* mutant. Importantly, with the exception of *nrdR*, we did not identify other genes for putative transcriptional regulators that were downregulated in *sigE* or *sigK* mutant (Table 2). Overall, our data indicates that in *C. difficile*, the mother cell line of gene expression is deployed according to a hierarchical regulatory cascade simpler than in *B. subtilis* [14].

The regulation of mother cell sigma factors synthesis in *C. difficile* also differs from other Clostridia. In *C. perfringens*, a biphasic pattern of *sigK* expression (early and late in sporulation) is observed and σ^E^ and σ^K^ coregulate the expression of each other [21]. In *C. botulinum*, *sigK* is expressed at the onset of stationary phase and σ^K^ positively controls the expression of *sigF* and *spo0A* [73]. In *C. difficile*, we did not observe a control of *spo0A*, *sigF* or *bona fide* members of the σ^E^ regulon by σ^K^ (Table 2). In both *C. perfringens* and *C. botulinum*, sporulation is arrested at an early stage in a *sigK* mutant and not at a late stage as observed in *B. subtilis* and *C. difficile* [5], [19]. It is also worth noting that the *sigK* gene is disrupted by a *skin* element in *B. subtilis* and *C. difficile* but not in *C. acetobutylicum*, *C. perfringens* and *C. botulinum*, suggesting that excision of this element may be an important factor in controlling the timing of σ^K^ activity in *C. difficile*.

### V. Communication between the forespore and the mother cell

A hallmark of sporulation in *B. subtilis* is the existence of cell-cell signaling pathways that link the forespore and mother cell-specific lines of gene expression. Because these pathways operate at critical morphological stages of sporulation, the result is the coordinated deployment of the two lines of gene expression, in close register with the course of morphogenesis [5], [6]. Indeed, σ^F^ is required for the activation of pro-σ^E^ into σ^E^ in the mother cell and σ^E^ in turn is necessary to activate σ^G^ in the forespore. Finally, σ^G^ is required for the activation of pro-σ^K^ into σ^K^ in the mother cell [5], [6]. We obtained new information on the intercompartment signaling pathway in *C. difficile*.

#### Absence of a strict control of the σ^E^ regulon by σ^F^

**Figure 3:**
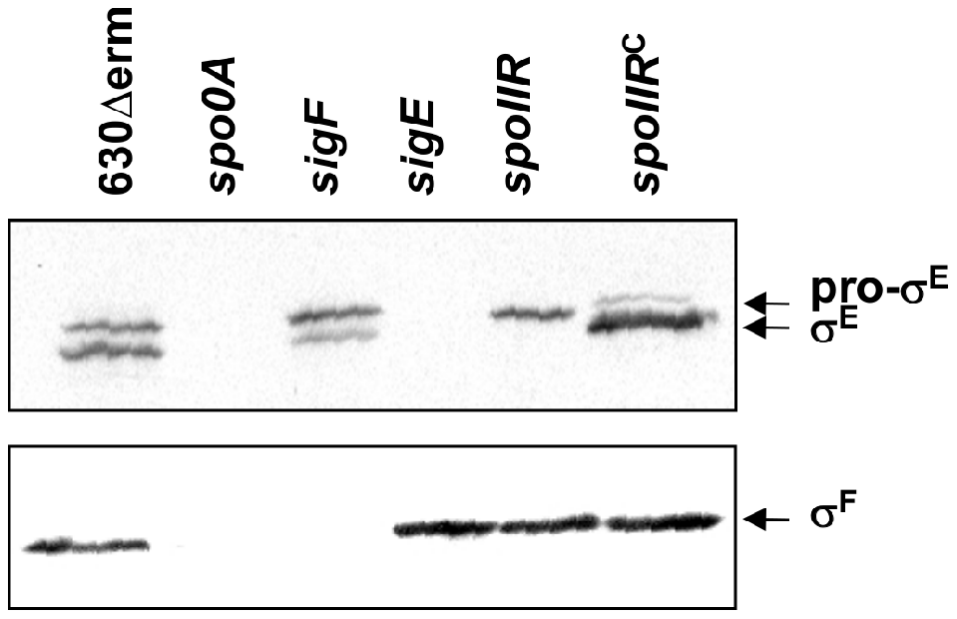
The involvement of σ^F^ and SpoIIR in the control of formation of the processed σ^E^ protein. 15 µg of total protein extracted from 630Δerm strain, the *spo0A*, *sigF*, *sigE* and *spoIIR* mutants and a complemented strain, CDIP246, a *spoIIR* mutant carrying a multicopy *spoIIR* gene controlled by its native promoter were loaded on a SDS-PAGE (12%). The various samples were analysed by immunoblotting with polyclonal anti-σ^E^ and anti-σ^F^ antibodies as described in Materials and Methods.

In *B. subtilis*, the σ^E^ regulon is indirectly controlled by σ^F^, which is required for the proteolytical activation of pro-σ^E^. Surprisingly, we did not find any global control of the σ^E^ regulon by σ^F^ in *C. difficile* (Table S1 and Table S2). We confirmed by qRT-PCR that the expression of *bona fide* σ^E^ targets such as *spoIIIAA*, *spoIVA*, *spoIIID* or *CD2864* did not significantly decrease in a *sigF* mutant (Fig. S4). This is a major difference relative to the *B. subtilis* sporulation regulatory network [74] and indicates that σ^F^ is not strictly required for σ^E^ functionality in *C. difficile*. One possibility is that the SpoIIGA mediated proteolytical activation of pro-σ^E^ is not fully dependent on σ^F^. To test this hypothesis, we detected in several strains (630Δerm, *spo0A*, *sigF* and *sigE* strains) the σ^F^, σ^E^ and pro-σ^E^ polypeptides by Western-blotting using antibodies raised against either σ^F^ or σ^E^ (Fig. 3). In strain 630Δerm, we observed σ^F^ production while two forms corresponding to σ^E^ and pro-σ^E^ were detected with an anti-σ^E^ antibody. In a *spo0A* mutant, neither σ^E^, pro-σ^E^ nor σ^F^ were detected (Fig. 3). This result is in agreement with the strong decrease of expression of the *spoIIAA* operon in a *spo0A* mutant [27]. As shown by qRT-PCR, *spo0A* inactivation also led to a 240-fold decrease of *sigE* expression indicating that this regulator controls pro-σ^E^ synthesis. It has also been shown that Spo0A directly binds with low affinity to the *spoIIGA* and *spoIIAA* promoter regions [30]. In a *sigE* mutant, we did not detect σ^E^ or pro-σ^E^ with an anti-σ^E^ antibody while we specifically detected σ^F^ with an anti-σ^F^ antibody. Interestingly, in a *sigF* mutant in which σ^F^ was absent, we detected pro-σ^E^ but also the processed σ^E^ form in a reduced quantity compared to the situation in a wild-type strain (Fig. 3). The expression of the σ^E^ regulon in the *sigF* mutant shows that even reduced the level of active σ^E^ in this mutant is sufficient to allow the transcription of σ^E^-controlled genes. This clearly demonstrates that an active σ^E^ protein can be produced in the absence of σ^F^ in *C. difficile* contrary to *B. subtilis* [75]. In *C. perfringens* and *C. acetobutylicum*, neither pro-σ^E^ nor σ^E^ protein are produced in a *sigF* mutant [22], [23] suggesting a diversity in the mode of forespore control of σ^E^ activity among spore forming Firmicutes.

#### The role of SpoIIR in the regulatory cascade

**Figure 4:**
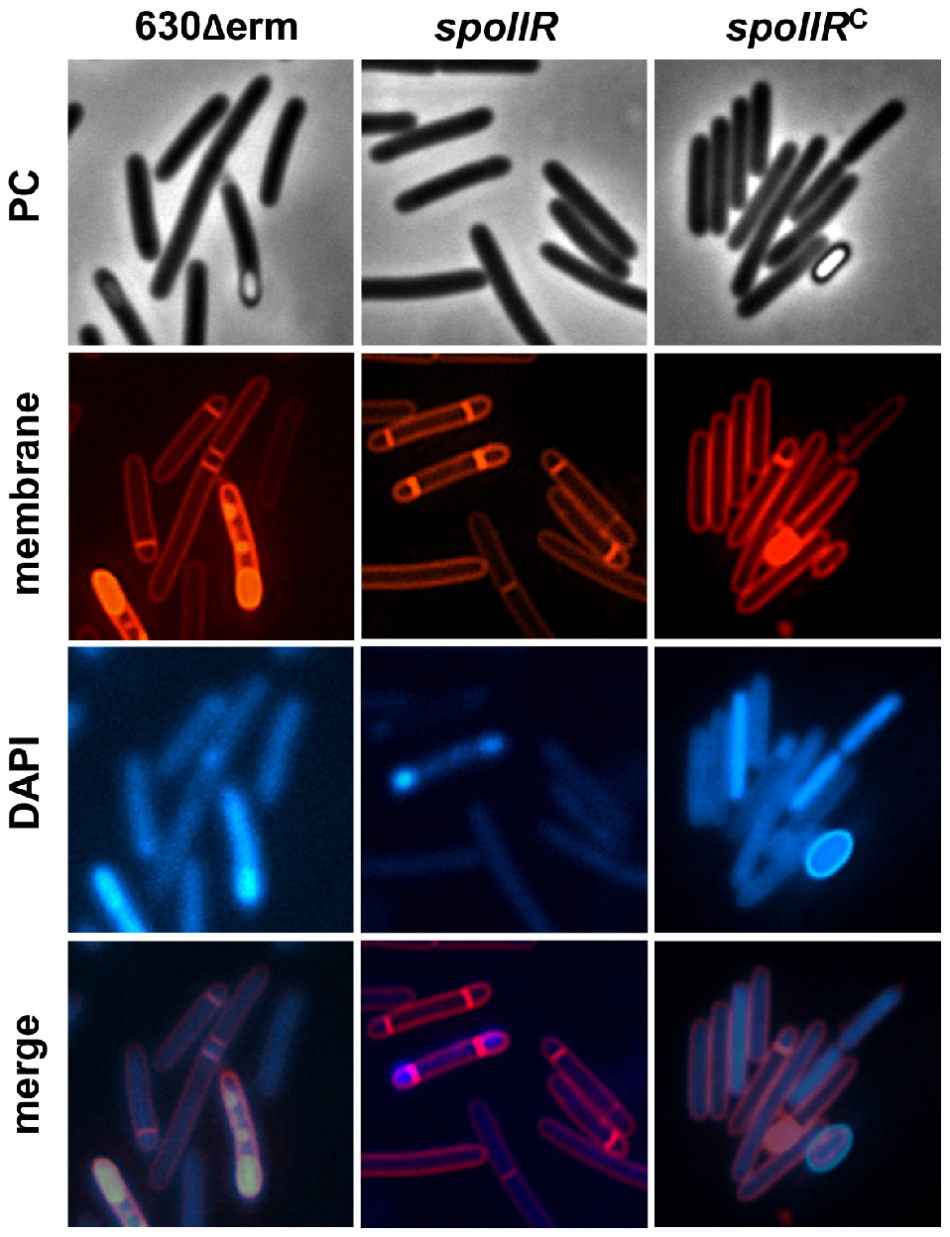
Morphological characterization of a *spoIIR* mutant. Cells of the *C. difficile* 630Δerm strain, the *spoIIR* mutant and the complemented strain, CDIP246, carrying a multicopy *spoIIR* gene controlled by its native promoter were collected after 24 h of growth in SM broth, stained with the DNA stain DAPI and the membrane dye FM4-64 and examined by phase contrast and fluorescence microscopy.

In *B. subtilis*, σ^F^ drives production of the signaling protein SpoIIR, which is secreted across the forespore inner membrane into the intermembrane space, where it stimulates the SpoIIGA-dependent pro-σ^E^ processing in the mother cell [6], [11], [76]. The results exposed above raised the possibility that SpoIIR is dispensable for pro-σ^E^ processing in *C. difficile*, or that expression of the *spoIIR* gene is partially independent on σ^F^. To determine the role of SpoIIR in *C. difficile*, we inactivated *spoIIR*. A complemented strain, CDIP246, carrying a multicopy allele of *spoIIR* under the control of its native promoter was also constructed. We first examined the morphology of the *spoIIR* mutant. Phase contrast and fluorescence microscopy experiments showed that the *spoIIR* mutant was blocked at the asymmetric septation stage and accumulated disporic cells (Fig. 4) as shown for the *spoIIR* mutant in *B. subtilis* [11]. The wild-type phenotype was restored in the complemented strain CDIP246. The phenotype caused by the *spoIIR* mutation in *C. difficile* phenocopied that imposed by a *sigE* mutation [19] strongly suggesting that σ^E^ is inactive in this mutant. In *B. subtilis*, loss of σ^E^ or interference with its activation leads to disporic forms [35], [77]. We also measured the sporulation efficiency of these three strains after a heat treatment. After 72 h of growth in SM medium, the strain 630Δerm produced 2×10^6^ heat-resistant spores/ml. In contrast, no heat resistant spores were detected for the *spoIIR* mutant. When we complemented the *spoIIR* mutant using a pMTL84121-*spoIIR* plasmid, the titer of heat resistant spores was of 9×10^5^/ml. So, inactivation of the *spoIIR* gene resulted in a complete inability of *C. difficile* to sporulate.

To test the prediction that σ^E^ is inactive in the *spoIIR* mutant, we compared the accumulation of σ^F^, σ^E^ and pro-σ^E^ polypeptides in strain 630Δerm and in the *spoIIR* mutant by immunoblotting. σ^F^ and pro-σ^E^ but not σ^E^ accumulated in the *spoIIR* mutant (Fig. 3). To independently test the impact of the *spoIIR* mutation on the activity of σ^E^, the expression of selected σ^E^ target genes was analyzed by qRT-PCR. The expression of *spoIIIAA*, *spoIVA*, *spoIIID* and *sigK* genes decreased 10-, 8-, 16- and 23-fold in a *spoIIR* mutant compared to strain 630Δerm (Table S11) while the expression of *sigE* itself was not reduced in this mutant. When the pMTL84121-*spoIIR* plasmid was introduced into the *spoIIR* mutant, the expression of *spoIIIAA*, *spoIVA*, and *spoIIID* increased 10-, 12- and 7-fold compared to strain 630Δerm while the expression of the *sigK* gene was restored to the wild-type level. Therefore, both the immunoblot and the qRT-PCR studies support the idea that *spoIIR*, but not σ^F^, is strictly required for the activation of σ^E^ (Fig. 3, Table S11).

**Figure 5:**
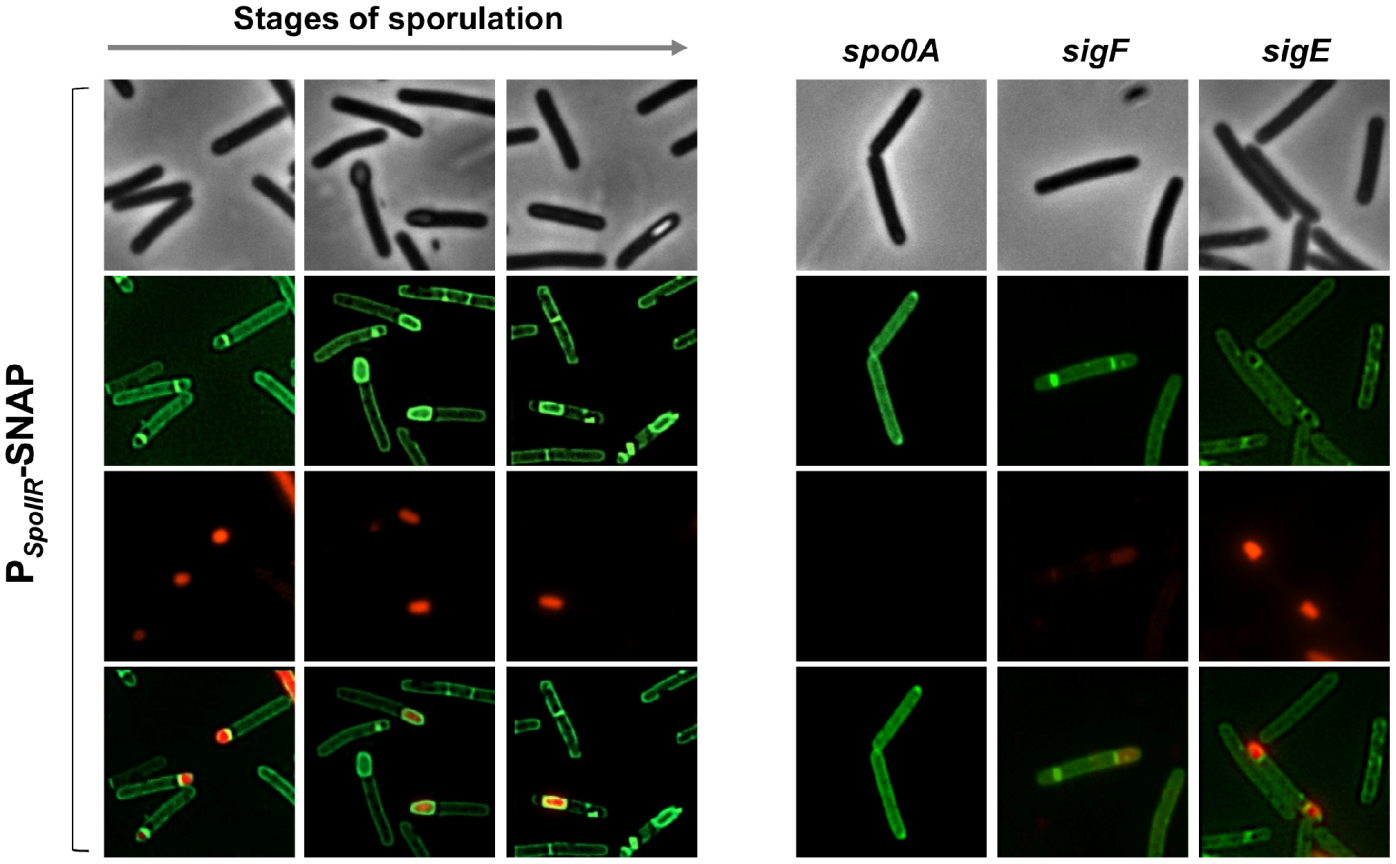
Fluorescence of a P*spoIIR*-SNAP fusion in strain 630Δerm and in a *spo0A*, *sigF* or *sigE* mutant. Cells of the *C. difficile* 630Δerm strain, and of the *spo0A*, *sigF* and *sigE* mutants carrying a P*_spoIIR_*-SNAP^Cd^ transcriptional fusion in a multicopy plasmid were collected 24 h of following inocculation in SM broth. Cells were labelled with the fluorescent substrate TMR to allow localization of SNAP^Cd^ production driven by the *spoIIR* promoter, stained with the DNA marker DAPI and the membrane dye MTG and examined by phase contrast and fluorescence microscopy.

It follows that the expression of *spoIIR* has to occur in part independently of σ^F^. We first showed that the expression of *spoIIR* decreased 2.5- and 38-fold in a *sigF* mutant in transcriptome and by qRT-PCR (Table 1 and Table S5) while the introduction of pMTL84121-*sigF* into the *sigF* mutant increased *spoIIR* expression 9-fold compared to strain 630Δerm (Table S9). So, the *spoIIR* transcription is under σ^F^ control. We also constructed a transcriptional fusion between the *spoIIR* promoter and the SNAP tag [19]. This P*spoIIR*-SNAP^Cd^ fusion was introduced by conjugation into strain 630Δerm and a *spo0A*, *sigF* or *sigE* mutant. Labeling with the fluorescent substrate TMR allowed localization of P*spoIIR*-SNAP^Cd^ expression. When examined by fluorescence microscopy, most of the cells of strain 630Δerm showed fluorescence in the forespore as expected for a gene under σ^F^ control (Fig. 5). *spoIIR* expression was not controlled by σ^E^ since a fluorescence signal was detected in the small compartments of disporic cells in a *sigE* mutant. Interestingly, a fluorescence of the *spoIIR*-SNAP^Cd^ fusion was still observed in the *sigF* mutant suggesting that some expression of *spoIIR* occurs in the absence of σ^F^. In a *spo0A* mutant, the fluorescence completely disappeared and the *spoIIR* expression strongly decreased by qRT-PCR. So, the residual expression observed in the *sigF* mutant is likely Spo0A-dependent. Presumably, the expression of *spoIIR* in the *sigF* mutant allows the production of sufficient SpoIIR to trigger pro-σ^E^ processing. It is worth noting that *spoIIR* is not downregulated in a *sigF* mutant in *C. acetobutylicum* and only partially under σ^F^ control in *C. difficile* (Fig. 5). Also interestingly, in *B. subtilis*, the forced expression of *spoIIR* in pre-divisional cells, still allows pro-σ^E^ processing to occur, even in the absence of σ^F^ [11], [78]. It is possible that the activation of σ^E^ in ancestral spore formers occurred independently of the forespore. The expression of *spoIIR* under the exclusive control of σ^F^ may have appeared later and would allow a better coordination between the forespore and mother cell lines of gene expression.

#### The regulation of σ^G^-dependent genes by σ^E^

In *B. subtilis*, most of the σ^G^ activity occurs after engulfment completion. In addition, the expression of *sigG* target genes in the engulfed forespore depends upon σ^E^ activation in the mother cell at least in part through synthesis of the SpoIIIA proteins [5], [6], [37], [38]. In the transcriptome analysis, we observed that the expression of 6 genes belonging to the σ^G^ regulon (*sspB*, *CD1486*, *CD1631*, *CD1880*, *CD2465* and *CD3551.1*) was downregulated in the *sigE* mutant (Table 3). We also examined by qRT-PCR if a *sigE* mutation affected the expression of several other σ^G^ targets. The expression of 9 additional σ^G^-controlled genes decreased in a *sigE* mutant (Table 3). Interestingly, SpoIIID also repressed the expression of 12 members of the σ^G^ regulon (*sspA*, *sspB*, *spoVT*, *CD1631*, *CD1463*, *CD2112*, *CD2245.1*, *CD2375*, *CD2808*, *CD2809*, *CD3489*, *CD3610*) in transcriptome (Table S10). This clearly indicated that both σ^E^ and SpoIIID participated in the control of σ^G^-target genes in *C. difficile*. In *B. subtilis*, σ^G^ activity is dependent on the SpoIIIA-SpoIIQ channel [6], [37], [38]. The SpoIIIA proteins are encoded by the octacistronic *spoIIIAA-AH* operon, which is expressed in the mother cell under the direct control of σ^E^ and SpoIIID [9], [12], [14]. As noted above, the *spoIIIAA* operon is a member of the σ^E^ and *spoIIID* regulons also in *C. difficile* (Table 2 and Table S10) and is transcribed from two promoters recognized by σ^E^ located upstream of the *spoIIIAA* and *spoIIIAG* cistrons (Table S7), as observed in *B. subtilis* [79]. It thus seems possible that control of forespore-specific gene expression by σ^E^ and SpoIIID involves similar mechanisms in *C. difficile*. However, we note that fluorescence from a P*sspA*-SNAP^Cd^ fusion is detected in the forespore compartment of *sigE* mutant cells [19]. This indicates that a strict requirement for σ^E^ is not observed for the expression of *C. difficile* σ^G^-targets contrary to what is shown in *B. subtilis* suggesting a less tight control of σ^E^ on gene expression in the forespore.

#### The absence of control of the σ^K^ regulon by σ^G^

In *B. subtilis*, *sigG* regulates the expression of the σ^K^ regulon in the mother cell through the control of pro-σ^K^ processing [5], [6]. A *sigG* mutant is blocked just after engulfment completion, and does not show any signs of assembly of the surface layers around the forespore. Surprisingly, no σ^K^ target genes were downregulated in a *sigG* mutant in the transcriptome analysis and this lack of effect of the *sigG* mutation was confirmed by qRT-PCR for eight σ^K^-controlled genes (*cotA*, *cotCB*, *cotD*, *bclA1*, *bclA2*, *bclA3*, *CD1067*, *CD3620*). Thus, synthesis of the spore coat proteins belonging to the σ^K^ regulon (CotA, CotCB, CotD, CotE, CotF) is σ^G^-independent. This result is in agreement with the phenotype of the *C. difficile sigG* mutant that shows deposition of some coat material around the engulfed forespore [19]. Interestingly, mutations that bypass the need for σ^G^ for pro-σ^K^ processing, or a pro-less allele of the *sigK* gene in *B. subtilis* allow expression of σ^K^ targets [80]. In *C. difficile*, the pro-sequence of σ^K^ is absent and no homologs of SpoIVFB, the membrane-embedded protease that processes pro-σ^K^ in *B. subtilis* and of two other membrane proteins (SpoIVFA and BofA) that form a complex with and control the localization and activity of SpoIVFB, can be found [15], [17], [25], [81]. Thus, σ^K^ activity is not controlled through processing of a pre-protein in *C. difficile* and this may be related to the absence of σ^G^ control. However, both the synthesis and the processing of pro-σ^K^ are also σ^G^ independent in *C. perfringens* [23]. Therefore, the absence of control of σ^K^ by σ^G^ is not restricted to Clostridia lacking a pro-σ^K^ protein.

Intriguingly, *C. difficile* codes for two homologs of the *B. subtilis* signaling protein SpoIVB. CD1213 and CD0783 share 37% and 36% identity with SpoIVB, respectively. *CD0783* and *CD1213* belong to the σ^F^ and σ^G^ regulons, respectively (Table 1). In *B. subtilis*, SpoIVB is a protease synthesized under the control of σ^F^ and σ^G^. Similarly, *spoIIP* is transcribed by σ^F^ and σ^E^ in *B. subtilis*, whereas two *spoIIP* genes are present in *B. anthracis* and transcribed by σ^F^ and σ^E^, respectively [82]. SpoIVB is secreted to the intermembrane space where it cleaves SpoIVFA releasing SpoIVFB and allowing pro-σ^K^ processing [5], [6]. However, *B. subtilis* SpoIVB has two distinct developmental functions: pro-σ^K^ processing control and another role essential for spore formation probably corresponding to the cleavage of SpoIIQ [83], [84]. It is possible that the *C. difficile* SpoIVB-like proteins retained the second role, which might correspond to an ancestral function.

Unexpectedly, 14 σ^K^ target genes were downregulated in a *sigF* mutant compared to strain 630Δerm in transcriptome (*cotA, cotCB, bclA1, bclA2, bclA3, cwpV, CD0896 CD1067, CD1581, CD1891, CD2184, CD2537, CD2664, CD3620*). We further showed by qRT-PCR that 4 additional σ^K^ target genes (*sleC*, *cotD*, *cotE* and *CD1133*) were positively controlled by σ^F^ (Table 3) suggesting a more global regulation of σ^K^ targets by σ^F^. This effect might be mediated through an indirect control by σ^F^ of the synthesis or the activity of σ^K^ itself or of another regulator of members of the σ^K^ regulon. Another hypothesis might be that the σ^K^ activity requires engulfment completion a process blocked in a *sigF* mutant but not in a *sigG* mutant. Interestingly, the synthesis of pro-σ^K^ and σ^K^ in *C. perfringens* is also abolished in a *sigF* mutant but not in a *sigG* mutant [23] suggesting a switch in the forespore control of σ^K^-activity from σ^G^ to σ^F^. However, a *sigK* mutant of *C. perfringens* is blocked early in sporulation, at the asymmetric division stage [21]. The molecular mechanisms involved in the σ^F^-dependent control of late mother cell-specific gene expression remain to be determined.

#### Conclusion

Global approaches combining transcriptome and TSS mapping allow us to have a view of the expression pattern during the early and late stages of sporulation in both forespore and mother cell and to define the four compartment-specific sigma regulons in *C. difficile*. We identified 25, 97, 50 and 56 members for the σ^F^, σ^E^, σ^G^ and σ^K^ regulons, respectively and in each regulon, we found key representatives of the homologous regulons of *B. subtilis* as proposed previously [16]. However, while larger than the evolutionary conserved core machinery for endosporulation, of about 145 genes [15], [17], this set of *C. difficile* sporulation genes (around 225 genes) corresponds to about half the number of genes under the control of cell type-specific sigma factors of *B. subtilis* [9], [10], [12]. A more dynamic study with a detailed kinetic analysis would provide additional members for each regulon but it is also probable that the presumably more ancestral type of sporulation process proposed for Clostridia [15], [17], [18], [20] involves a smaller collection of proteins. This has already been observed for the initiation of sporulation where the complex signaling transduction pathway involving in *B. subtilis* a phosphorelay that modulates Spo0A activity is replaced by a simple two-component system [18], [20], [29]. In general, the most significant variations between the *B. subtilis* and *C. difficile* sporulation process are observed at the interface with their environment: the signal transduction pathway triggering sporulation initiation, composition of the coat shell and the germination-activating pathways [16], [18], [20], [47]. Indeed, the germination signals and germination receptors differ among spore forming Firmicutes and only a few spore coat layer proteins are conserved in Bacilli and Clostridia [47], [57]. In each regulon, a significant fraction of genes encodes proteins with unknown function but our work offer new insights about the role of some of them. Some σ^G^-controlled membrane proteins might be involved in germination, which remain poorly characterized in *C. difficile* [48], [55]. Three proteins of unknown function belonging to the σ^E^ regulon (CD0214, CD1930, CD3522) and several proteins synthesized under σ^K^ control (CD1063.1, CD1067, CD1133, CD3580 and CD3613) that are detected in the spore proteome [39] are probably new spore coat components. Finally, we found several oxygen detoxification proteins that might help in the long-term survival of clostridial spores as recently proposed by Galperin *et al* [17]. This probably favors the dissemination of spores of strictly anaerobic Clostridia in aerobic environment, a crucial step for persistence and transmission of pathogenic Clostridia [1], [2].

**Figure 6:**
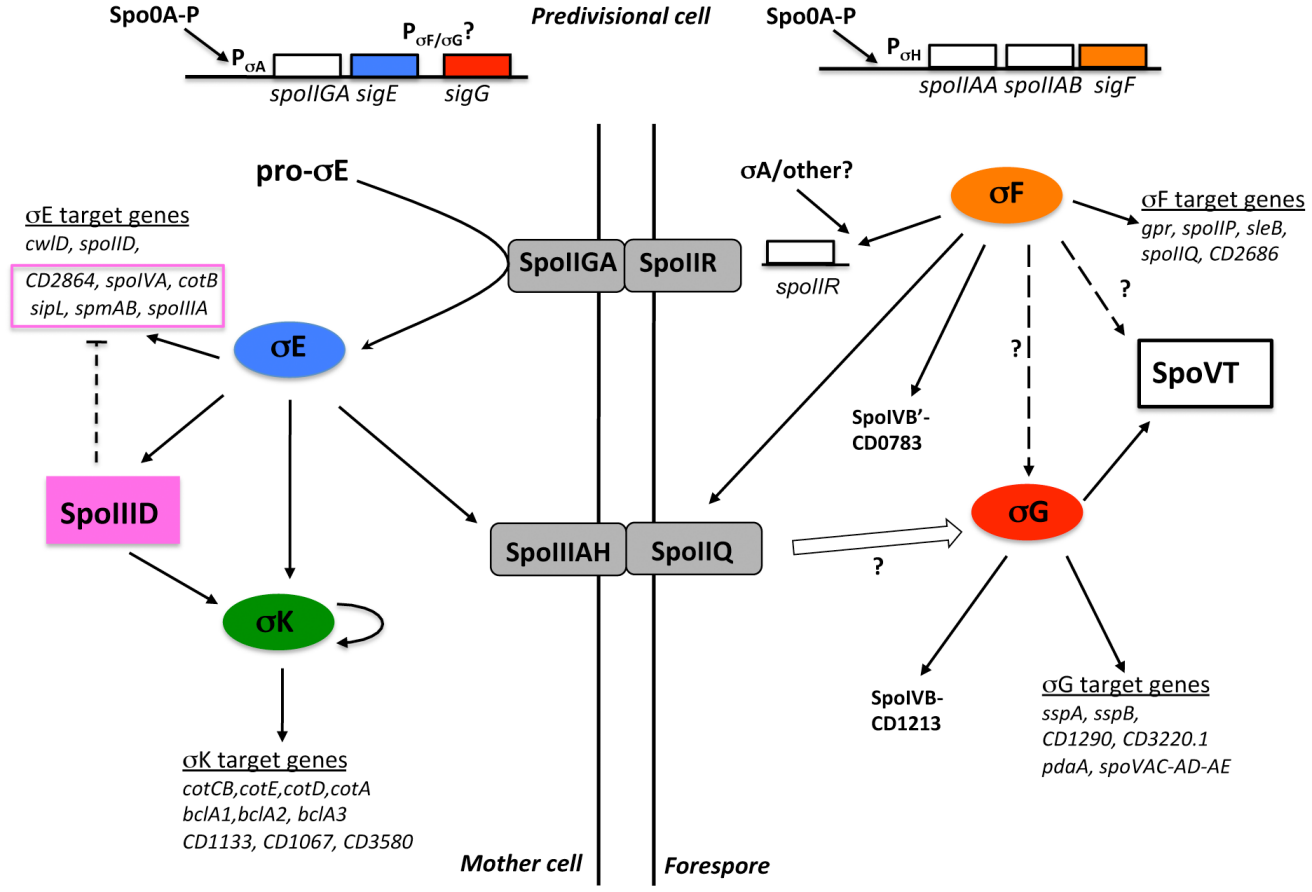
Model of the regulatory network controlling sporulation in *C. difficile*. The upper part of the figure represents the cell before asymmetric division. The parallel vertical lines represent the two membranes separating the forespore (right) and the mother cell (left). The four sigma factors of sporulation are encircled (by oval boxes). Precursor protein of σ^E^ is indicated as pro-σ^E^. Square boxes correspond to transcriptional regulators. Proteins associated with the membrane or located into the intermembrane space are illustrated as embedded in the parallel vertical lines. Black solid arrows and white arrow indicate activation at the transcriptional level or at the level of protein activity, respectively. Broken arrows with a question mark represent mechanisms that are not yet fully understood. Dotted arrows indicate a possible direct transcriptional activation or repression. The σ^E^ target genes under the negative control of SpoIIID are inside a pink box.

In this work, we also exposed important deviations from the *B. subtilis* paradigm in *C. difficile*. Both the global analysis of the program of gene expression under the control of the four cell type-specific sigma factors and the morphological characterization of the corresponding mutants (this work, [19]) indicate that coupling between gene expression and morphogenesis is less tight in *C. difficile* than in *B. subtilis* (Fig. 6). First, the σ^E^ regulon in the *C. difficile* mother cell is not strictly under σ^F^ control despite the fact that the forespore product SpoIIR is required for pro-σ^E^ processing. The residual *spoIIR* expression in the *sigF* mutant might be responsible for this less strict connection. Second, the tight coordination between σ^G^ and σ^K^ activities observed in *B. subtilis*, is absent in *C. difficile* since σ^K^ activity does not depend on σ^G^ as clearly shown by the morphology of a *sigK* mutant [19]. In the absence of a σ^K^ precursor, the rearrangement of the *sigK* gene associated to excision of the *skin* element may be the only way to control the timing of σ^K^ activity in *C. difficile* [16], [20], [25] and this event does not appear to be under σ^G^ control. However, a control of the forespore on σ^K^ target genes seems to be maintained through a σ^F^-dependent regulation.

Recent data discussed above also suggest differences in the regulatory network controlling sporulation among Clostridia. This is especially the case for the impact of each sigma factor inactivation on the synthesis of the others and for the role of σ^K^ [21], [23], [73], [85]. The timing of *sigK* expression, the phenotype of *sigK* mutants and/or some σ^K^ targets differ. In addition, with the exception of *C. difficile*, σ^K^ activity is controlled through processing in Clostridia while the insertion of a *skin* element in the *sigK* gene is found only in *C. difficile* strains [25]. The rather low probability to observe orthologs in clostridial genomes for many *C. difficile* regulon members also suggests a moderate conservation of the sporulation sigma-factor regulons among Clostridia. This work gives new insights about the diversity and evolution of the sporulation process. The sporulation in *C. difficile* might reflect a more ancestral way of sporulation while a more sophisticated system of developmental control would have been gradually introduced during evolution.

## Materials and Methods

### Bacterial strains, growth conditions and sporulation assay

*C. difficile* strains and plasmids used in this study are presented in Table 4. *C. difficile* strains were grown anaerobically (10% H_2_, 10% CO_2_, and 80% N_2_) in TY or in Brain Heart Infusion (BHI, Difco), which was used for conjugation. Sporulation medium (SM) [86] was used for sporulation assays. SM medium (pH at 7.4) contained per liter : 90 g Bacto tryptone, 5 g Bacto peptone, 1 g (NH_4_)_2_SO_4_, 1.5 g Tris Base. When necessary, cefoxitin (Cfx; 25 µg/ml), thiamphenicol (Tm; 15 µg/ml) and erythromycin (Erm; 2.5 µg/ml) were added to *C. difficile* cultures. *E. coli* strains were grown in Luria-Bertani (LB) broth. When indicated, ampicillin (100 µg/ml) or chloramphenicol (15 µg/ml) was added to the culture medium.

**Table 4:** Strains and plasmids used in this study.

Strains	Genotype	Origin
***E. coli***		
TOP 10	F^−^ *mcr*A D(*mrr-hsdRMS-mcrBC*) f80*lac*ZΔM15 Δ*lac*X74 *deo*R, *rec*A1 *ara*D139 D(*ara-leu*)7697 *gal*K *rps*L(StrR) *end*A1 *nup*G	Invitrogen
BL21 (DE3)	F^−^ *ompT gal dcm lon hsdS*_B_(r_B_^−^ m_B_^−^) λ(DE3 [*lacI* lacUV5-T7 gene 1 *ind1 sam7 nin5*])	New England Biolabs
HB101 (RP4)	*supE*44 *aa*14 *galK*2 *lacY*1 Δ (*gpt-proA*) 62 *rpsL*20 (Str^R^)*xyl-5 mtl-1 recA*13 Δ (*mcrC-mrr*) *hsdS*_B_(r_B_^−^m_B_^−^) RP4 (Tra^+^ IncP Ap^R^ Km^R^ Tc^R^)	laboratory stock
***C. difficile***		
630Δerm	630 Δ*erm*	[88]
CDIP3	630Δerm *spo0A*::*erm*	[27]
CDIP224	630Δerm *spoIIID*::*erm*	This work
CDIP227	630Δerm *spoVT*::*erm*	This work
CDIP238	630Δerm *spoIIR*::*erm*	This work
CDIP246	630Δerm *spoIIR*::*erm* pMTL84121-*spoIIR*	This work
CDIP262	630Δerm *spoIIID*::*erm* pMTL84121-*spoIIID*	This work
CDIP263	630Δerm *spoVT*::*erm* pMTL84121-*spoVT*	This work
AHCD533	630Δerm *sigF*::*erm*	[19]
AHCD532	630Δerm *sigE*::*erm*	[19]
AHCD534	630Δerm *sigG*::*erm*	[19]
AHCD535	630Δerm *sigK*::*erm*	[19]
AHCD548	630Δerm *sigF*::*erm* pMTL84121-*sigF*	[19]
AHCD549	630Δerm *sigE*::*erm* pMTL84121-*sigE*	[19]
AHCD550	630Δerm *sigG*::*erm* pMTL84121-*sigG*	[19]
AHCD551	630Δerm *sigK*::*erm* pMTL84121-*sigK^skin+^*	[19]
AHCD606	630Δerm P*spoIIR*-SNAP^Cd^	This work
AHCD622	630Δerm *spo0A*::*erm* P*spoIIR*-SNAP^Cd^	This work
AHCD623	630Δerm *sigF*::*erm* P*spoIIR*-SNAP^Cd^	This work
AHCD624	630Δerm *sigE*::*erm* P*spoIIR*-SNAP^Cd^	This work
**Plasmids**		
pFT34	pET28a-*sigF*-*his*_6_	This work
pFT35	pET28a-*sigE*-*his*_6_	This work
pFT47	pMTL84121-SNAP^Cd^	[19]
pMS462	pFT47-P*spoIIR*-SNAP^Cd^	This work
pMS459	pMTL007::Cdi-*CD3564*-38a	This work
pDIA6123	pMTL007::Cdi-*CD0216-*39s	This work
pDIA6124	pMTL007::Cdi-*CD3499-*157a	This work
pDIA6132	pMTL84121-*spoVT*	This work
pDIA6133	pMTL84121-*spoIIID*	This work
pDIA6135	pMTL84121-*spoIIR*	This work

Sporulation assay were performed as follows. After 72 h of growth in SM medium, 1 ml of culture was divided into two samples. To determine the total number of cells, the first sample was serially diluted and plated on BHI with 0.1% taurocholate (Sigma-Aldrich). Taurocholate is required for the germination of *C. difficile* spores [4]. To determine the number of spores, the vegetative bacteria of the second sample were heat killed by incubation for 30 min at 65°C prior to plating on BHI with 0.1% taurocholate. The percentage of sporulation was determined as the ratio of the number of spores/ml and the total number of bacteria/ml (×100).

### Construction of *C. difficile* strains

The ClosTron gene knockout system [87] was used to inactivate the *spoIIID* (*CD0126*), *spoVT* (*CD3499*) and *spoIIR* (*CD3564*) genes giving strains CDIP224 (630Δerm *CD0126*::*erm*), CDIP227 (630Δerm *CD3499*::*erm*) and CDIP238 (630Δerm *CD3564*::*erm*). Primers to retarget the group II intron of pMTL007 to these genes (Table S12) were designed by the Targetron design software (http://www.sigmaaldrich.com). The PCR primer sets were used with the EBS universal primer and intron template DNA to generate by overlap extension PCR, a 353-bp product that would facilitate intron retargeting to *CD0126*, *CD3499* or *CD3564*. The PCR products were cloned between the HindIII and BsrGI sites of pMTL007 to yield pDIA6123 (pMTL007::Cdi-*CD0216*-39s), pDIA6120 (pMTL007::Cdi-*CD3499*-157a) and pMS459 (pMTL007::Cdi-*CD3564*-38a). The presence and orientation of the insert in pMS459 was verified by DNA sequencing using the pMTL007-specific primers pMTL007-F and pMTL007-R. pDIA6120, pDIA6123 and pMS459 were introduced into *E. coli* HB101 (RP4) and the resulting strains subsequently mated with *C. difficile* 630Δerm [88]. *C. difficile* transconjugants were selected by sub-culturing on BHI agar containing Tm and Cfx and then plated on BHI agar containing Erm. Chromosomal DNA of transconjugants was isolated as previously described. PCR using the ErmRAM primers (ErmF and ErmR) confirmed that the Erm resistant phenotype was due to the splicing of the group I intron from the group II intron following integration. In order to verify the integration of the Ll.LtrB intron into the right gene targets, we performed PCR with two primers flanking the insertion site in *CD0126* (LS184-LS185), *CD3499* (LS186-LS187) or *CD3564* (IMV649-LS113) and in one hand with the intron primer EBSu and in other hand with a primer in *CD0126* (LS184), *CD3499* (LS187) or *CD3564* (LS113).

To complement the *spoIIR* mutant, the *spoIIR* gene with its promoter (−169 to +791 from the translational start site), was amplified by PCR using oligonucleotides IMV642 and IMV641 (Table S12). To complement the *spoIIID* and the *spoVT* mutants, the *spoIIID* gene with its promoter (−117 to +320 from the translational start site) and the *spoVT* gene with its promoter (−165 to +731 from the translational start site), were amplified by PCR using oligonucleotides LS283 and IMV647 or IMV644 and IMV648 (Table S12). The PCR fragments were cloned into the XhoI and BamHI sites of pMTL84121 [89] to produce plasmids pDIA6132 (*spoVT*), pDIA6133 (*spoIIID*) and pDIA6135 (*spoIIR*). Using the *E. coli* HB101 (RP4) strain containing pDIA6135 as donor, this plasmid was transferred by conjugation into the *C. difficile spoIIR* mutant giving strain CDIP246 (Table 4). Similarly the plasmids pDIA6132 and pDIA6133 were transferred by conjugation into the *spoVT* or *spoIIID* mutant giving strains CDIP263 and CDIP262.

To construct a P*spoIIR*-SNAP fusion reporter strain, a 470 bp fragment encompassing the region upstream of the *spoIIR* gene was amplified by PCR using primers PspoIIR-SNAP EcoRIFw and PspoIIR-SNAP XhoIRev (Table S12). The PCR product was inserted between the EcoRI and XhoI sites of pFT47 [19] to create pMS462. pMS462 was introduced into *E. coli* HB101 (RP4) and then transferred to *C. difficile* 630Δerm, *spo0A*::*erm*, *sigF*::*erm* or *sigE*::*erm* strains by conjugation. Transconjugants were selected on BHI agar plates containing Tm and Cfx followed by plating on BHI agar containing Tm.

### RNA extraction, quantitative real-time PCR and transcriptional start site mapping

For RNA-seq experiment allowing TSS mapping, total RNA was isolated from *C. difficile* 630 strain grown in TY medium after 4 h and 10 h of growth or under starvation conditions that correspond to a 1 h resuspension of exponentially growing cells (6 h of growth) into phosphate-buffered saline (PBS: 137 mM NaCl, 10 mM Phosphate, 2.7 mM KCl, pH 7.4) [31]. The Tobacco Acid Pyrophosphatase (TAP)+/− library construction and high-throughput sequencing was realized on a mixed sample combining RNAs extracted from these three different growth conditions as previously described [31]. After Illumina sequencing, the reads were mapped to the *C. difficile* genome using Bowtie [90] then converted into BAM files with the Samtools [91]. The data were visualized at a strand-specific manner using COV2HTML (http://mmonot.eu/COV2HTML/). All TSS detected by RNA-seq were inspected manually. Deep sequencing data are available at https://mmonot.eu/COV2HTML/visualisation.php?str_id=-14.

To perform transcriptional analysis for each sigma factor, we first tested the impact of their inactivation on the expression of two target genes at different times to optimize conditions. For this purpose, we used *bona fide* targets of these sigma factors in *B. subtilis* [16]: *gpr* and *spoIIR* for σ^F^, *spoIIIAA* and *spoIVA* for σ^E^ and *sspA* and *sspB* for σ^G^. For σ^K^, we used *cotCD* and *cotA* encoding recently described *C. difficile* spore coat proteins [41]. This preliminary test allowed us to define a time for maximal differential expression for these targets between wild-type and mutant strains for each sigma factor. To study σ^E^- or σ^F^-dependent control, we harvested cells of strain 630Δerm, the *sigE* and the *sigF* mutants after 14 h of growth in SM medium. The strain 630Δerm and the *sigG* or the *sigK* mutant were harvested after 19 h (630Δerm, *sigG* mutant) and 24 h (630Δerm, *sigK* mutant) of growth in SM medium. For the *spoIIR* and *spoIIID* mutants, we harvested cells after 14 h and 15 h of growth in SM medium, respectively. The culture pellets were resuspended in RNApro solution (MP Biomedicals) and RNA extracted using the FastRNA Pro Blue Kit, according to the manufacturer's instructions. The RNA quality was determined using RNA 6000 Nano Reagents (Agilent). For the *spo0A* mutant and the 630Δerm strain, RNA previously obtained were used to test the impact of Spo0A inactivation on gene expression [27].

Quantitative real-time PCR (qRT-PCR) analysis was performed as previously described [27]. The primers used for each marker are listed in Table S12. In each sample, the quantity of cDNAs of a gene was normalized to the quantity of cDNAs of the DNApolIII gene. The relative change in gene expression was recorded as the ratio of normalized target concentrations (ΔΔCt) [92].

### Microarray design, DNA-array hybridization and transcriptome analysis

The microarray of *C. difficile* 630 genome (GEO database accession number GPL10556) was designed as previously described [27]. Transcriptome was performed using for each condition four (630Δerm compared to the mutant inactivated for each sigma factor) or two different RNA preparations (630Δerm compared to *spoIIID*). Labeled DNA hybridization to microarrays and array scanning were done as previously described [27]. The slides were analyzed using R and limma software (Linear Model for Microarray Data) from Bioconductor (www.bioconductor.org). We corrected background with the ‘normexp’ method, resulting in strictly positive values and reducing variability in the log ratios for genes with low levels of hybridization signal. Then, we normalized each slide with the ‘loess’ method [93]. To test for differential expression, we used the bayesian adjusted t-statistics and we performed a multiple testing correction of Benjamini and Hochberg based on the false discovery rate (FDR) [94]. A gene was considered as differentially expressed when the p-value is <0.05. The complete data set was deposited in the GEO database with a series record accession number GSE43202.

### In silico analysis of promoters

We analyzed the TSS data by the following *in silico* iterative strategy. For σ^F^, σ^E^, σ^G^ and σ^K^, we first worked with a reduced training set corresponding mainly to promoters located upstream of genes having an orthologous gene controlled by the same sigma factor in *B. subtilis* [16], of genes encoding experimentally characterized spore coat proteins for σ^K^ [41] or of genes encoding proteins associated with the spore [39] (Table S8). Half-sites (boxes) were then manually re-aligned to maximize similarity and to construct recognition profiles for each sigma factor. The score of a candidate promoter was defined as the sum of positional nucleotide weights W(b_i_,i), with an additional term V(k) to account for preferred interbox spacer length: S(b_1_…b_m_x_1_…x_k_b_m+1_b_m+n_) = σ_i = 1…m+n_ W(b_i_,i)+V(k), where b_1_…b_m_, b_m+1_b_m+n_ are nucleotides in the promoter boxes, x denotes any nucleotide, m and n are the distal and proximal box lengths, respectively, k is the spacer length. Positional nucleotide weights were defined as W(b,i) = log (N(b,i)+0.5)−0.25xσ_d = A,T,G,C_ log (N(d,i)+0.5), where N(b,i) is the count of nucleotide b at alignment position i, and box length weight was defined as V(k) = log (M(k)+0.5)−(1/(k_max_−k_min_+1))xσ_j = kmin…kmax_ log (M(j)+0.5), k_max_ and k_min_ are the maximal and minimal observed spacer length, respectively, and M(k) is the count of spacer length k. The necessary procedures were implemented in SignalX [95]. These profiles determined with the training set were further used to score all identified TSS. Candidate promoters scoring higher than the lowest-scoring promoter in the training set were retained for further analysis (Table S8). A powerful approach, as for transcription factors, is to apply comparative techniques based on the assumptions that functional regulatory sites should be conserved in related species. Genome sequences from *C. difficile* six closest relatives from the *Clostridium* genus (*C. saccharolyticum*, *C. thermocellum*, *C. tetani*, *C. cellulolyticum*, *C. ljungdahlii*, *C. kluyveri*) and *B. subtilis* 168 were downloaded from GenBank. We searched the intergenic regions of the six Clostridia using the profiles defined for *C. difficile* (Table S8). As the exact TSS positions in these species are unknown, we analyzed regions (−100 +10) relative to annotated start codons. The data on *B. subtilis* promoters were obtained from DBTBS [96]. Finally, for each candidate promoter, we calculated the number of genomes also having a candidate promoter upstream of an orthologous gene. Orthologs were defined using the bidirectional best hit criterion implemented in GenomeExplorer [95].

### Purification of σ^F^-His_6_ and σ^E^-His_6_ for antibody production

The coding sequences of *sigF* and *sigE* were amplified by PCR using primer pairs CDsigF-pET28aFw/CDsigF-pET28aRev and CDsigE-pET28aFw/CDsigE-pET28aRev, respectively. The resulting fragments were introduced between the NcoI and XhoI sites of pET28a to produce pFT35 (σ^F^-His_6_) and pFT34 (σ^E^-His_6_). BL21(DE3) derivatives carrying pFT34 or pFT35 were grown in LB to an OD_600 nm_ of 0.5 and induced with 1 mM IPTG for 4 h. The cells were then harvested by centrifugation at 4000 *g*, ressuspended in 20 mM phosphate, 1 mM phenylmethyl-sulfonyl fluoride (PMSF), 10 mM imidazole, and lysed using a French pressure cell (18000 lb/in^2^). After centrifugation, the supernatant (or, for the case of σ^E^-His_6_, the sediment after solubilization with 8M Urea for 30 min) was loaded onto a 1 ml Histrap column (Amersham Phamarcia Biotech). The bound proteins were eluted with a discontinuous imidazole gradient. The purified proteins were used for the production of rabbit polyclonal antibodies (Davids Biotechnologie GmbH).

### Whole cell lysates and immunoblot analysis

To prepare *C. difficile* whole cell extracts, 10 ml samples were withdrawn from SM cultures at the desired times following inoculation and the cells collected by centrifugation (4000 *g*, 10 min, at 4°C). Cells were lysed in 1 ml buffer (10 mM Tris pH 8.0, 10 mM MgCl_2_, 0.5 mM EDTA, 0.2 mM NaCl, 10% glycerol, 1 mM PMSF) using a French pressure cell. Samples (15 µg of protein) were resolved by 12% SDS-PAGE, transferred to a nitrocellulose membrane (BioRad), and subjected to immunoblot analysis as described previously [97]. Antibodies against σ^F^ and σ^E^ were used at a 1∶2000 dilution. A rabbit secondary antibody conjugated to horseradish peroxidase (Sigma) was used at a 1∶5000 dilution. The immunoblots were developed with enhanced chemiluminescence reagents (Amersham Pharmacia Biotech).

### Microscopy and image analysis

1 ml samples were withdrawn from SM cultures at the desired times following inoculation, and the cells collected by centrifugation (4000 *g*, 10 min, at 4°C). The cells were washed with 1 ml of PBS and ressuspended in 0.1 ml of PBS supplemented with the membrane dye FM4-64 (10 µg.ml^−1^) and the DNA stain DAPI (4′,6-diamidino-2-phenylindole; 50 µg.ml^−1^) (Molecular Probes, Invitrogen). For SNAP staining, 1 ml samples were stained for 30 min with 50 nM SNAP-Cell TMR-Star (New England Biolabs) as described [19]. Cells were washed four times by centrifugation (4000 *g*, 5 min) and ressupended in 1 ml of PBS. Following washing, the cells were ressuspended in 1 ml of PBS supplemented with the membrane dye Mitotracker Green (0.5 µg.ml^−1^) (Molecular Probes, Invitrogen). Cells were mounted on 1.7% agarose coated glass slides and imaged in a Leica DM6000B microscope as previously described [64]. Fluorescent signals were visualized with a phase contrast Uplan F1 100× objective and captured with a CCD Andor Ixon^EM^ camera (Andor Technologies). Images were acquired and analyzed using the Metamorph software suite version 5.8 (Universal Imaging).

## Supporting Information

Figure S1 Alignment of the σ^E^ and σ^K^ proteins of *B. subtilis* and *C. difficile*. The amino acid sequences from σ^E^ and σ^K^ of *B. subtilis* and *C. difficile* are aligned. The amino acids conserved in these four sequences are indicated by a star. The region 4.2, which may interact with the −35 regions of their cognate promoters is underlined. In *B. subtilis*, the specificity of interaction of these sigma factors with the −35 region sequences (a T for σ^E^ and a C for σ^K^) is associated with the presence of a glutamine at position 217 of σ^E^ and of an arginine in σ^K^ [32]. These amino acids are indicated in red and blue, respectively.
(PDF)

Figure S2 Inactivation of the *spoIIR, spoIIID and spoVT* genes in *C. difficile* using the ClosTron system. **A**: Schematic representation of gene inactivation by a type II Intron with an associated RAM. The group II intron (bracket), originally in pMTL007 (top), carries a RAM element (yellow) interrupting an ermB determinant (blue). The intron was retargeted to the sig gene of interest (black; middle) by altering the IBS, EBS1 and EBS2 sequences (grey and white stripes; top) by overlapping PCR. Splicing out of the td group I intron from the ermB gene in the RAM restores a functional marker allowing positive selection of mutants following intron integration. Primers used to confirm the integration and orientation of the type II intron are also indicated (bottom). Genetic organisation of the *C. difficile* chromosome in the vicinity of *spoIIR spoIIID* and *spoVT*. The red arrow indicates the point of insertion of the re-targeted type II introns used for gene disruption. The extent of the DNA fragment present in the indicated replicative plasmids used for in trans complemetation of the insertional mutations is shown below each of the genetic maps, except for the *sigK* gene (see also Fig. 6). **B**: Chromosomal DNA of Em^R^ *C. difficile* conjugants and of strain 630Δerm were screened by PCR using primer pairs RAM-F/R to confirm splicing out of the group I intron in the mutant (lane 1 and 2). To verify the integration of the Ll.LtrB intron into the right gene targets, we further performed PCR using chromosomal DNA of strain 630Δerm and of each mutant (lane 3 and 4) with two primers flanking the insertion site in *CD0126-spoIIID* (LS184-LS185), *CD3499-spoVT* (LS186-LS187) or *CD3564-spoIIR* (IMV649-LS113). Finally, we also performed PCR using chromosomal DNA of strain 630Δerm and of each mutant (lane 5 and 6) with the intron primer EBSu in one hand and with a primer in *CD0126-spoIIID* (LS184) in *CD3499*-*spoVT* (LS187) or *CD3564-spoIIR* (LS113) in other hand. Chromosomal DNA from the 630Δerm strain corresponded to lane 1, 3 and 5 while chromosomal DNA of each mutant corresponded to lane 2, 4 and 6. The smart ladder (Eurogentec) was used as a molecular weight marker. **C**: Southern blot analysis of genomic DNA from *C. difficile* 630Δerm, *spoIIR*, *spoIIID*, and *spoVT* mutant strains with an intron probe. Chromosomal DNA (6 µg in each reaction) was digested with *Hind*III. Southern blot analyses were performed using Amersham ECL Direct Nucleic Acid labelling and detection reagents, in accordance with the manufacturer's guidelines and visualised using Super Signal West Femto Maximum Sensitivity Substrate (Thermo Scientific). The probe was produced by PCR using OBD522 and OBD523 primers (Table S12), designed within the group II intron sequence.
(PDF)

Figure S3 Alignment of the SpoIIID regulator of several Bacilli and Clostridia. The amino acid sequences from SpoIIID of *B. subtilis* (bs), *B. cereus* (bc), *B. antracis* (ba), *C. acetobutylicum* (ca), *C. perfringens* (cp) and *C. difficile* (cd) are aligned. The amino acids conserved in these four sequences are indicated by a star. The two regions essential for DNA binding are indicated: an helix-turn-helix motif (HTH) and a basic region near the C-terminus part of the protein [68].
(PDF)

Figure S4 Control of σ^E^ targets by σ^F^ and σ^E^. Total RNAs were extracted from *C. difficile* 630Δerm strain, the *sigF* mutant and the *sigE* mutant grown in SM medium for 14 h. After reverse transcription, specific cDNAs were quantified by qRT-PCR using DNA PolIII gene for normalization (See Materials and Methods). The expression ratio of strain 630Δerm/*sigE* and 630Δerm/*sigF* were indicated in white and black, respectively. Error bars correspond to standard deviation from at least two biological replicates.
(PDF)

Table S1 List of genes controlled by σ^F^ in transcriptome. A gene is considered differentially expressed between the 630Δerm strain and the *sigF* mutant when the P value is <0.05 using the statistical analysis described in Materials and Methods. We did not include genes with a fold-change <2-fold. However, some genes with a fold-change less than 2-fold were included when they appeared to be in the same transcription unit with regulated genes for which the fold-change was ≥2 or when they were regulated by other sigma factors of sporulation.
(PDF)

Table S2 List of genes controlled by σ^E^ in transcriptome. A gene is considered differentially expressed between the 630Δerm strain and the *sigE* mutant when the *P* value is <0.05 using the statistical analysis described in Materials and Methods. We did not include genes with a fold-change <2-fold. However, some genes with a fold-change less than 2-fold were included when they appeared to be in the same transcription unit with regulated genes for which the fold-change was ≥2 or when they were regulated by other sigma factors of sporulation.
(PDF)

Table S3 List of genes controlled by σ^G^ in transcriptome. A gene is considered differentially expressed between the 630Δerm strain and the *sigG* mutant when the P value is <0.05 using the statistical analysis described in Materials and Methods.
(PDF)

Table S4 List of genes controlled by σ^K^ in transcriptome. A gene is considered differentially expressed between the 630Δerm strain and the *sigK* mutant when the *P* value is <0.05 using the statistical analysis described in Materials and Methods. We did not insert in this list genes with a fold-change <2-fold. However, some genes had a fold-change less than 2-fold but were included because they appeared to be in the same transcription unit with regulated genes for which the fold-change was ≥2 or because they were regulated by other sigma factors of sporulation.
(PDF)

Table S5 Validation of microarrays data by qRT-PCR on selected genes. qRT-PCR experiments were performed on two different RNA preparations for each mutant. The results presented corresponded to the mean of at least two independent experiments.
(PDF)

Table S6 Identification of promoters recognized by σ^F^ and σ^G^. The transcriptional start sites and the −10 and −35 boxes are indicated in red and blue, respectively. The position of 5′ start was identified by RNA-seq analysis with indicated score corresponding to the read length (51 bases) coverage ratio for TAP+ and TAP− samples. For σ^F^ and σ^G^, the genes underlined are those validated by *in silico* analysis (see Materials and Methods) and listed in Table S8. TSS can be visualized at https://mmonot.eu/COV2HTML/visualisation.php?str_id=-14.
(PDF)

Table S7 Identification of promoters recognized by σ^E^ and σ^K^. The transcriptional start sites and the −10 and −35 boxes are indicated in red and blue, respectively. The position of 5′ start was identified by RNA-seq analysis with indicated score corresponding to the read length (51 bases) coverage ratio for TAP+ and TAP− sample. For σ^E^ and σ^K^, the genes underlined are those validated by in silico analysis (see Materials and Methods) and listed in Table S8. TSS can be visualized at https://mmonot.eu/COV2HTML/visualisation.php?str_id=-14.
(PDF)

Table S8 *In silico* validation of promoters located upstream of genes identified in transcriptome as regulated by σ^F^, σ^E^, σ^G^ or σ^K^. We analyzed the promoters identified by TSS mapping by an iterative *in silico* strategy (see Materials and Methods). The training sets of genes used to first construct the recognition profiles for σ^F^, σ^E^, σ^G^ and σ^K^ were highlighted in yellow. For each promoter the score was obtained as defined in materials and methods. Using the same profiles, we searched the intergenic regions (positions (−100 +10) relative to start codons) of six closely related *Clostridium* species, *C. saccharolyticum*, *C. thermocellum*, *C. tetani*, *C. cellulolyticum*, *C. ljungdahlii*, *C. kluyveri*. We also analyzed the *B. subtilis* promoters using the DBTBS database [96]. The absence of an orthologous gene is indicated by 0. When an orthologous gene is present the score and the locus-tag are indicated.
(PDF)

Table S9 Complementation of the mutants inactivated for the sigma factors of sporulation. qRT-PCR experiments were performed on two different RNA preparations for each mutant and each complemented strain. Cells were harvested after 14 h of growth for the strain 630Δerm, the *sigE* and *sigF* mutants and the *sigF* mutant containing pMTL84121-*sigF* and the *sigE* mutant containing pMTL84121-*sigE*, after 20 h of growth for the strain 630Δerm, the *sigG* mutant and the *sigG* mutant containing pMTL84121-*sigG* and after 24 h of growth for the strain 630Δerm, the *sigK* mutant and the *sigK* mutant containing pMTL84121-*sigK^skin+^*. The results presented correspond to the mean of at least two independent experiments.
(PDF)

Table S10 List of genes controlled by SpoIIID in transcriptome. RNA was extracted from strain 630Δerm strain and the *spoIIID* mutant after 15 h of growth in SM medium. A gene is considered differentially expressed between the 630Δerm strain and the *spoIIID* mutant when the P value is <0.05 using the statistical analysis described in Materials and Methods.
(PDF)

Table S11 Control of expression of sporulation genes by SpoIIID, SpoVT or SpoIIR. Cells of the 630Δerm strain and of the *spoIIID* mutant were harvested after 15 h or 24 h of growth in SM medium. Cells of the 630Δerm strain and of the *spoVT* mutant were harvested after 15 h or 20 h of growth in SM medium. Cells of the 630Δerm strain and of the *spoIIR* mutant were harvested after 14 h of growth in SM medium. qRT-PCR experiments were performed on two different RNA preparations. The results presented corresponded to the mean of at least two independent experiments. NR = not regulated.
(PDF)

Table S12 List of oligonucleotides.
(PDF)
