# Supplementary material for: Genome-Wide Analysis of Cell Type-Specific Gene Transcription during Spore Formation in Clostridium difficile
Source: PLoS Genet. 2013 Oct 3;9(10):e1003756. doi: 10.1371/journal.pgen.1003756 (PMC3789822; doi:10.1371/journal.pgen.1003756)
Supplement: Figure S1 — Alignment of the σE and σK proteins of B. subtilis and C. difficile. The amino acid sequences from σE and σK of B. subtilis and C. difficile are aligned. The amino acids conserved in these four sequences are indicated by a star. The region 4.2, which may interact with the −35 regions of their cognate promoters is underlined. In B. subtilis, the specificity of interaction of these sigma factors with the −35 region sequences (a T for σE and a C for σK) is associated with the presence of a glutamine at position 217 of σE and of an arginine in σK [32]. These amino acids are indicated in red and blue, respectively. (PDF) [file pgen.1003756.s001.pdf]

Figure S1. Alignement of the σ^E and σ^K proteins of *B. subtilis* and *C. difficile*

```
SigE-CD         MLRLKERIISFITMLGIKLKIIKPKGIYYMGGANILPPPLKPEEEMELLQKLET--DESV
SigE-BS         MKKLKLRLTHLWYKLLMKLGL-KSDEVYYIGGSEALPPPLSKDEEQVLLMKLPNG-DQAA
SigK-CD                                      MAALKSFEKPLTPEEEIEYLTKFKIENDKSA
SigK-BS               MVTGVFAALGFVVKE--LVFLVSYVKNNAFPQPLSSSEEKKYL-ELMAKGDEHA
                              *                  : :  **. .**   * ::    *: .

SigE-CD         KSILIERNLRLVVYISRKFENTGIDVEDLISIGTIGLIKAVNTFKLNKNIKLATYASRCI
SigE-BS         RAILIERNLRLVVYIARKFENTGINIEDLISIGTIGLIKAVNTFNPEKKIKLATYASRCI
SigK-CD         KDTLIERNMRLVAYIAKKYNNSTEDQDDLISIGTIGLIKAIETYNIDKGTRLATYASRCI
SigK-BS         RNMLIEHNLRLVAHIVKKFENTGEDAEDLISIGTIGLIKGIESYSAGKGTKLATYAARCI
                :  ***:*:***.:* :*::*:  : :************.::::.  *  :*****:***

SigE-CD         ENEI----------------------------------------------------LMYL
SigE-BS         ENEI----------------------------------------------------LMYL
SigK-CD         ENEI----------------------------------------------------LMNI
SigK-BS         ENEIVITKGGCIHPSLIRFNIYGVRIHNGNFFHDKVNNCFFIFKSMPPLFVMNNEILMHL
                ****                                                    ** :

SigE-CD         RKNNKKKTEVSFDEPLNIDLDGNELLLSDVLGTENDEIYKIIEEEIDRDLL-VMALDRLS
SigE-BS         RRNNKIRSEVSFDEPLNIDWDGNELLLSDVLGTDDDIITKDIEANVDKKLL-KKALEQLN
SigK-CD         RSNKKNKTQVSLQDPIGTDKEGNEISLLDILGTEANYVLDEVELKVQVGKLYEQLNKILT
SigK-BS         RALKKTKKDVSLHDPIGQDKEGNEISLIDVLKSENEDVIDTIQLNMELEKV-KQYIDILD
                *  :* :.:**:.:*:. * :***: * *:* :: : : . :: :::   :     . *

SigE-CD         DREKQIMELRFGLIDKGIEKTQKEVAGMLGISQSYISRLEKKIISRLQKEMKKFV-----
SigE-BS         EREKQIMELRFGLVGEE-EKTQKDVADMMGISQSYISRLEKRIIKRLRKEFNKMV-----
SigK-CD         PREREIVQLRYGLTPYG-YKTQREIAQKLDISRSYVSRIEKKALKKLEKELVQES-----
SigK-BS         DREKEVIVGRFGLDLKK-EKTQREIAKELGISRSYVSRIEKRALMKMFHEFYRAEKEKRK
                 **::::  *:**      ***:::*  :.**:**:**:**: : :: :*: :

SigE-CD         -----
SigE-BS         -----
SigK-CD         -----
SigK-BS         KAKGK
```
