# Supplementary figures and images for: Genome-Wide Analysis of Cell Type-Specific Gene Transcription during Spore Formation in Clostridium difficile
Source: PLoS Genet. 2013 Oct 3;9(10):e1003756. doi: 10.1371/journal.pgen.1003756 (PMC3789822; doi:10.1371/journal.pgen.1003756)

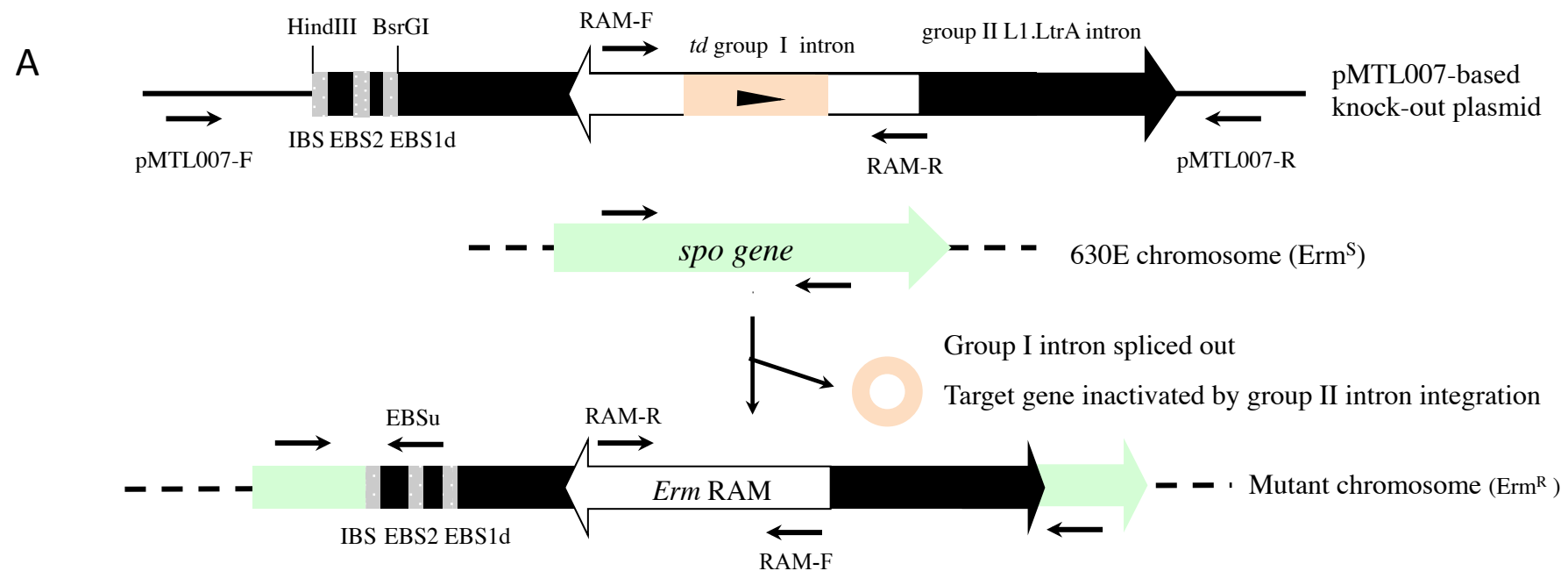

**B**

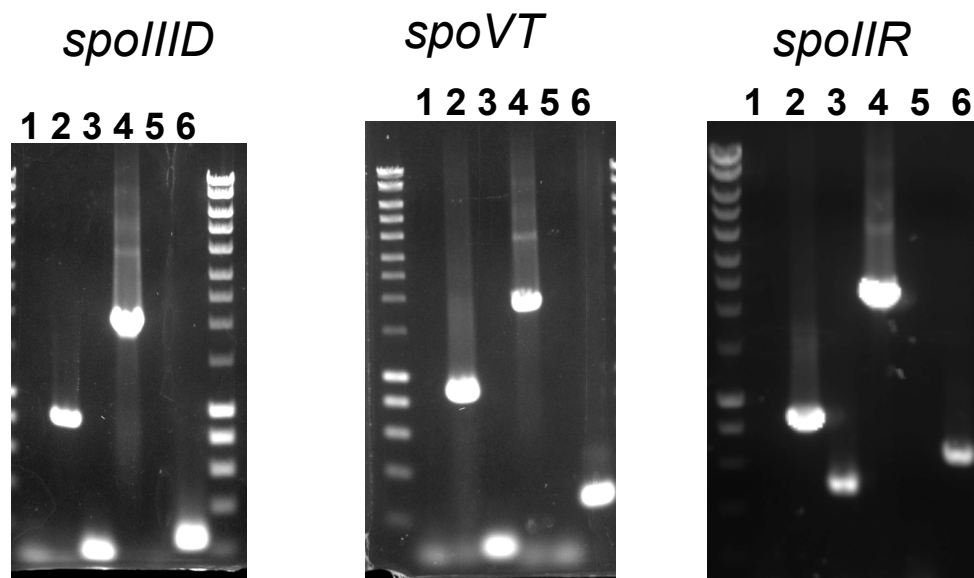

**C**

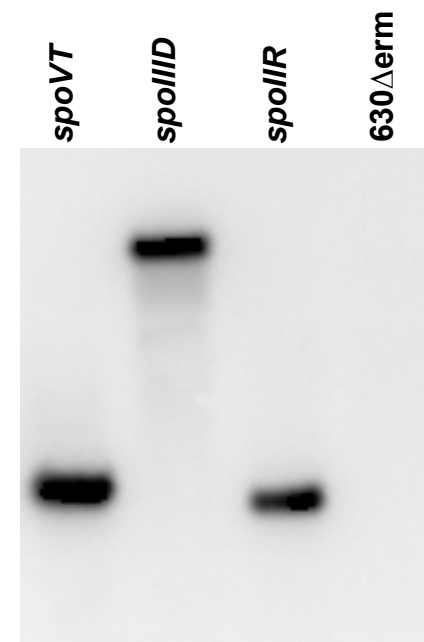

**Figure S2**

Supplement: Figure S2 — Inactivation of the spoIIR, spoIIID and spoVT genes in C. difficile using the ClosTron system. A: Schematic representation of gene inactivation by a type II Intron with an associated RAM. The group II intron (bracket), originally in pMTL007 (top), carries a RAM element (yellow) interrupting an ermB determinant (blue). The intron was retargeted to the sig gene of interest (black; middle) by altering the IBS, EBS1 and EBS2 sequences (grey and white stripes; top) by overlapping PCR. Splicing out of the td group I intron from the ermB gene in the RAM restores a functional marker allowing positive selection of mutants following intron integration. Primers used to confirm the integration and orientation of the type II intron are also indicated (bottom). Genetic organisation of the C. difficile chromosome in the vicinity of spoIIR spoIIID and spoVT. The red arrow indicates the point of insertion of the re-targeted type II introns used for gene disruption. The extent of the DNA fragment present in the indicated replicative plasmids used for in trans complemetation of the insertional mutations is shown below each of the genetic maps, except for the sigK gene (see also Fig. 6). B: Chromosomal DNA of EmR C. difficile conjugants and of strain 630Δerm were screened by PCR using primer pairs RAM-F/R to confirm splicing out of the group I intron in the mutant (lane 1 and 2). To verify the integration of the Ll.LtrB intron into the right gene targets, we further performed PCR using chromosomal DNA of strain 630Δerm and of each mutant (lane 3 and 4) with two primers flanking the insertion site in CD0126-spoIIID (LS184-LS185), CD3499-spoVT (LS186-LS187) or CD3564-spoIIR (IMV649-LS113). Finally, we also performed PCR using chromosomal DNA of strain 630Δerm and of each mutant (lane 5 and 6) with the intron primer EBSu in one hand and with a primer in CD0126-spoIIID (LS184) in CD3499-spoVT (LS187) or CD3564-spoIIR (LS113) in other hand. Chromosomal DNA from the 630Δerm strain correspond [file pgen.1003756.s002.pdf]

**Figure S3. Alignement of the SpoIIID regulator of several bacilli and clostridia.**

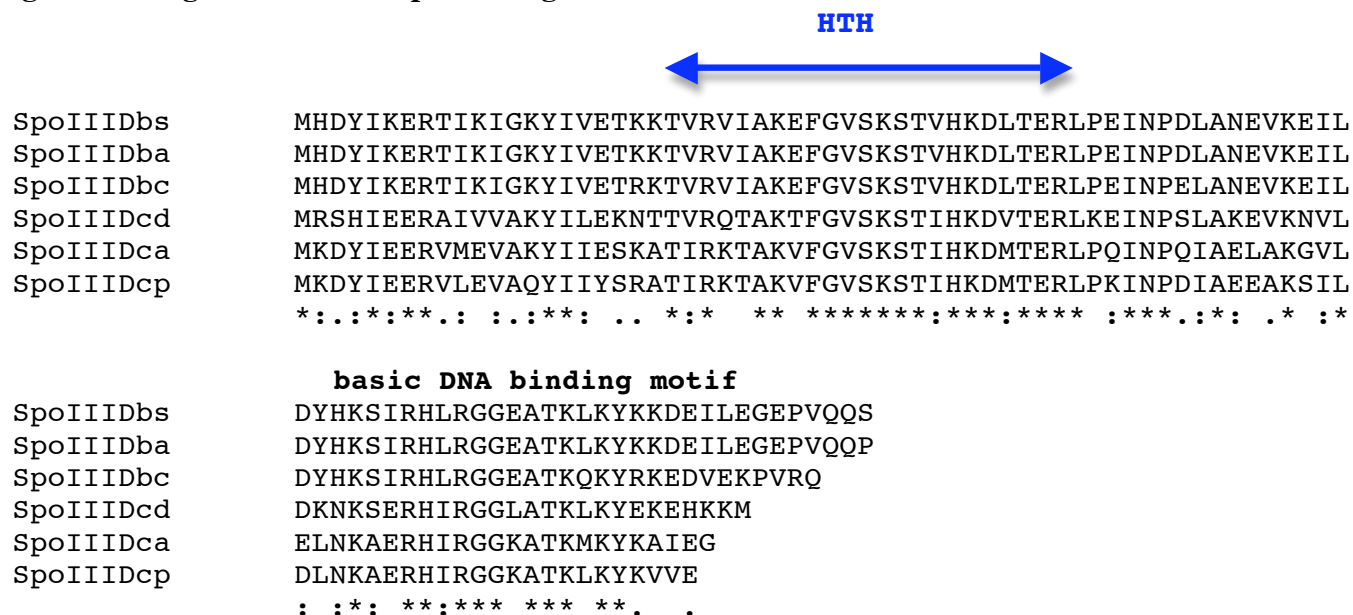

Supplement: Figure S3 — Alignment of the SpoIIID regulator of several Bacilli and Clostridia. The amino acid sequences from SpoIIID of B. subtilis (bs), B. cereus (bc), B. antracis (ba), C. acetobutylicum (ca), C. perfringens (cp) and C. difficile (cd) are aligned. The amino acids conserved in these four sequences are indicated by a star. The two regions essential for DNA binding are indicated: an helix-turn-helix motif (HTH) and a basic region near the C-terminus part of the protein [68]. (PDF) [file pgen.1003756.s003.pdf]

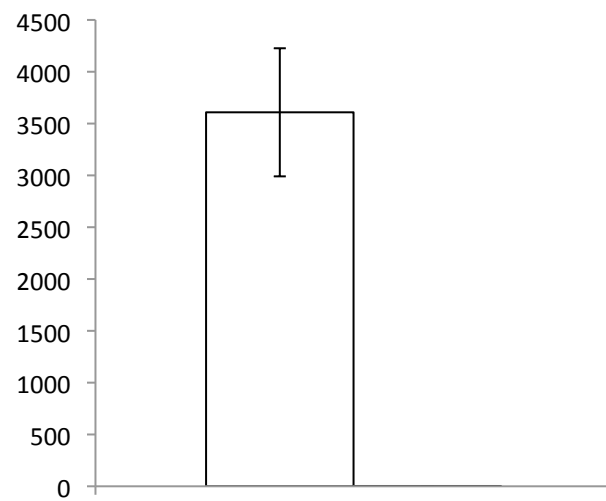

***spolIIAA***

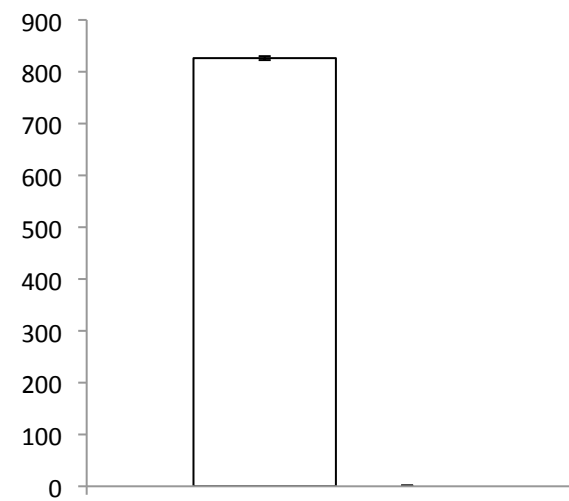

***spolIID***

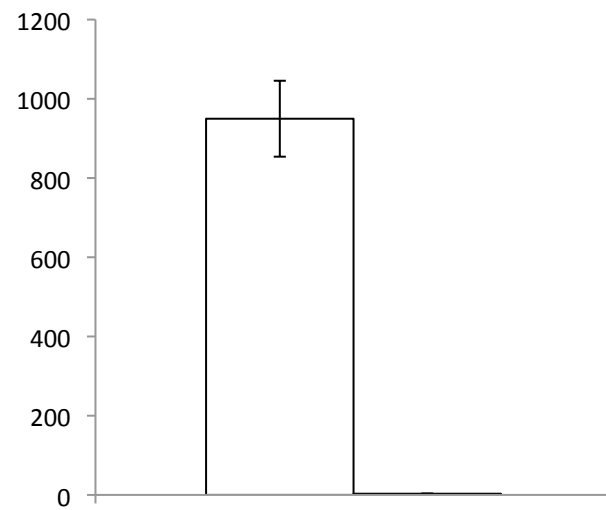

***spolIVA***

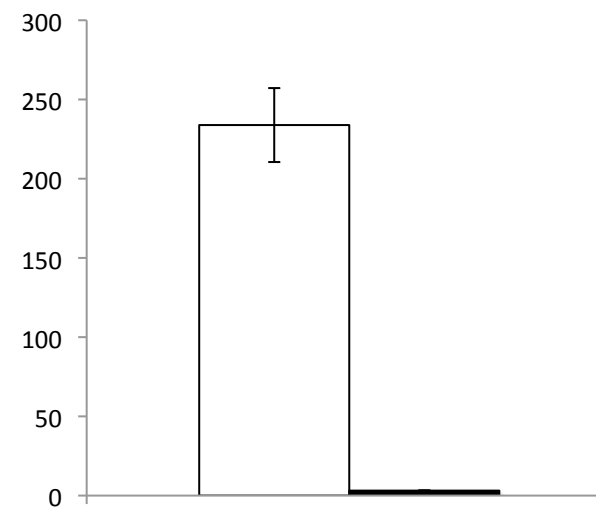

***CD2864***

**Figure S4.**

Supplement: Figure S4 — Control of σE targets by σF and σE. Total RNAs were extracted from C. difficile 630Δerm strain, the sigF mutant and the sigE mutant grown in SM medium for 14 h. After reverse transcription, specific cDNAs were quantified by qRT-PCR using DNA PolIII gene for normalization (See Materials and Methods). The expression ratio of strain 630Δerm/sigE and 630Δerm/sigF were indicated in white and black, respectively. Error bars correspond to standard deviation from at least two biological replicates. (PDF) [file pgen.1003756.s004.pdf]
