# Supplementary material for: Genome-Wide Analysis of Cell Type-Specific Gene Transcription during Spore Formation in Clostridium difficile
Source: PLoS Genet. 2013 Oct 3;9(10):e1003756. doi: 10.1371/journal.pgen.1003756 (PMC3789822; doi:10.1371/journal.pgen.1003756)
Supplement: Table S1 — List of genes controlled by σF in transcriptome. A gene is considered differentially expressed between the 630Δerm strain and the sigF mutant when the P value is <0.05 using the statistical analysis described in Materials and Methods. We did not include genes with a fold-change <2-fold. However, some genes with a fold-change less than 2-fold were included when they appeared to be in the same transcription unit with regulated genes for which the fold-change was ≥2 or when they were regulated by other sigma factors of sporulation. (PDF) [file pgen.1003756.s005.pdf]

Table S1. List of genes controlled by σ^F in transcriptome

Gene		Function	Expression ratio in transcriptome sigF/630Δerm
**Sporulation**			
CD0125	*spoIIQ*	Stage II sporulation protein Q	**0.11**
CD2470	*gpr*	Spore endopeptidase	**0.22**
CD2469	*spoIIP*	Stage II sporulation protein P	**0.14**
CD2468		Conserved hypothetical protein	**0.28**
CD3564	*spoIIR*	Pro-SigE endopeptidase signalling protein	**0.43**
CD3563	*sleB*	Spore-cortex-lytic protein	**0.16**
CD3499	*spoVT*	Stage V sporulation protein T	**0.07**
CD0783	*spoIVB'*	Stage IV sporulation protein SpoIVB, S55 peptidase family	**0.10**
CD0773	*spoVAC*	Stage V sporulation protein AC	**0.24**
CD0774	*spoVAD*	Stage V sporulation protein AD	**0.27**
CD0775	*spoVAE*	Stage V sporulation protein AE	**0.32**
CD2688	*sspA*	Small, acid-soluble spore protein alpha	**0.00**
CD3249	*sspB*	Small, acid-soluble spore protein alpha	**0.01**
CD1290		Putative small acid-soluble spore protein SASP	**0.29**
CD3220.1		Small acid-soluble spore protein	**0.28**
CD1230	*sigK*	Fragment of RNA polymerase sigma-K factor SigK (Part1)	**0.47**
CD1613	*cotA*	Spore outer coat layer protein CotA	**0.13**
CD0598	*cotCB*	Spore-coat protein CotCB manganese catalase	**0.18**
CD0214		Conserved hypothetical protein	**0.30**
CD0213		Putative spore coat protein	**0.47**
CD0332	*bclA1*	Putative exosporium glycoprotein	**0.43**
CD3230	*bclA2*	Putative exosporium glycoprotein	**0.16**
CD3349	*bclA3*	Exosporium glycoprotein BclA3	**0.12**
**Stress**			
CD1567		putative manganese catalase	**0.25**
CD2845	*rbr*	Rubrerythrin	**0.14**
CD1631	*sodA*	Superoxide dismutase (Mn)	**0.06**
CD3462	*mazE*	Putative antitoxin EndoAI	**0.20**
CD3461	*mazF*	Endoribonuclease toxin	**0.31**
**enveloppes**			
CD1430	*pdaA*	Putative δ-lactam-biosynthetic deacteylase	**0.06**
CD1291	*dacF*	D-alanyl-D-alanine carboxypeptidase	**0.11**
CD2141		Serine-type D-Ala-D-Ala carboxypeptidase	**0.30**
CD2184		Putative N-acetylmuramoyl-L-alanine amidase	**0.39**
CD3463	*alr2*	Alanine racemase 2	**0.23**
CD3464		Conserved hypothetical protein	**0.18**
CD0784		Putative N-acetylmuramoyl-L-alanine amidase	**0.23**
CD1229		Putative peptidoglycan glycosyltransferase	**0.42**
CD2664	*murE*	UDP-N-acetylmuramyl-tripeptide synthetase	**0.51**
CD2762	*uppS*	Putative undecaprenyl pyrophosphate synthetase	**0.20**
CD0514	cwpV	Cell surface protein	**0.61**
CD0792		Putative membrane protein, DUF81 family	**0.39**
CD0793		Putative membrane protein, DUF81 family	**0.26**
CD1297		Putative membrane protein	**0.31**
CD1298		Conserved hypothetical protein	**0.19**
CD1677		Putative membrane protein	**0.48**
CD1940		Putative membrane protein	**0.25**
CD2315		Putative exported protein	**0.31**
CD2634		Conserved hypothetical protein	**0.27**
CD2635		Putative membrane protein	**0.33**
CD2636		Putative membrane protein	**0.20**
CD2686		Putative membrane protein	**0.33**
CD2856		Putative membrane protein	**0.46**
CD3551.1		Putative membrane protein	**0.11**

CD1707		Putative C4-dicarboxylate anaerobic carrier, DcuC family	**0.29**
CD2107		Xanthine/uracil/thiamine/ascorbate permease family protein	**0.37**
CD1891		Fragment of ABC-type transport system, substrate-binding pr	**0.40**
CD2102		Putative Na(+)/H(+) antiporter	**0.49**
CD2465		Putative amino acid/polyamine transporter	**0.36**
**metabolism**			
CD0684		Putative ATP-dependent peptidase, M41 family	**0.29**
CD1319		Putative polysaccharide deacetylase	**0.37**
CD0580	*gapN*	Glyceraldehyde-3-phosphate dehydrogenase (NADP(+)) (GAI	**0.36**
CD1543		putative FMN-dependent NADH-azoreductase	**0.16**
CD2431		Putative nitrite/sulphite reductase	**0.23**
CD2661		Putative peptidase, M16 family	**0.47**
CD2660		Putative peptidase, M16 family	**0.48**
CD0047	*ispD*	2-C-methyl-D-erythritol 4-phosphate cytidylyltransferase	**0.48**
CD0048	*ispF*	2-C-methyl-D-erythritol 2,4-cyclodiphosphate synthase	**0.50**
CD3595		Aminopeptidase	**0.55**
CD1707		Putative C4-dicarboxylate anaerobic carrier, DcuC family	**0.29**
CD3521		Putative peptidase T, M20B family	**0.38**
CD2537		Membrane-associated 5'-nucleotidase/phosphoesterase	**0.42**
CD0649		Putative peptidase, S9 family	**3.80**
CD0650		Putative peptidase, S9 family	**5.21**
CD0651		Putative permease	**4.65**
CD1599	*thiD*	Phosphomethylpyrimidine kinase	**2.36**
CD1600	*thiM*	Hydroxyethylthiazole kinase	**2.23**
CD1601	*thiE*	Thiamine-phosphate pyrophosphorylase	**1.83**
CD1700	*ribD*	Riboflavin biosynthesis protein ribD [Includes: Diaminohydro›	**3.74**
CD1699	*ribE*	Riboflavin synthase alpha subunit	**2.31**
CD1908	*eutS*	Ethanolamine carboxysome structural protein	**3.60**
CD1909	*eutP*	Ethanolamine utilization protein	**2.69**
CD1910	*eutV*	Two-component response regulator, Ethanolamine specific	**2.22**
CD1911	*eutW*	Two-component sensor histidine kinase, Ethanolamine specifi	**2.15**
CD0108	*nrdD*	Anaerobic ribonucleoside triphosphate reductase	**1.95**
CD0109	*nrdG*	Anaerobic ribonucleoside-triphosphate reductase-activating p	**1.77**
**miscelaneous**			
CD1486		Putative ribosome recycling factor	**0.10**
CD0761		Putative ATP-dependent RNA helicase	**0.46**
CD3220		Putative methyltransferase	**0.39**
CD1323	tepA	Protein export-enhancing factor	**0.38**
**regulators**			
CD0648		Transcriptional regulator, GntR family	**2.79**
CD2928		Conserved hypothetical protein	**2.15**
CD2927		Transcriptional regulator, Phage-type	**2.33**
**Proteins of unknown function**			
CD0311		Conserved hypothetical protein	**0.32**
CD0347		Conserved hypothetical protein	**0.35**
CD0348		Fragment of conserved hypothetical protein (Part 2)	**0.35**
CD0543		Conserved hypothetical protein	**0.38**
CD0896		Conserved hypothetical protein	**0.21**
CD1067		Conserved hypothetical protein	**0.07**
CD1301.1		Conserved hypothetical protein	**0.28**
CD1354		Conserved hypothetical protein	**0.22**
CD1463		Conserved hypothetical protein	**0.06**
CD1581		Conserved hypothetical protein	**0.03**
CD1880		Conserved hypothetical protein	**0.07**
CD2112		Conserved hypothetical protein	**0.03**
CD2245.1		Conserved hypothetical protein	**0.10**
CD2375		Conserved hypothetical protein	**0.14**
CD2687		Conserved hypothetical protein	**0.36**
CD2808		Conserved hypothetical protein	**0.05**

CD2809	Conserved hypothetical protein, DUF1540 family	**0.05**
CD2816	Conserved hypothetical protein	**0.43**
CD3620	Conserved hypothetical protein	**0.35**
CD3271	Conserved hypothetical protein	**2.11**
CD2067	Conserved hypothetical protein	**2.05**

A gene is considered differentially expressed between the 630Δerm strain and the *sigF* mutant when the P value is <0.05 using the statistical analysis described in Materials and Methods. We did not include genes with a fold-change < 2-fold. However, some genes with a fold-change less than 2-fold were included when they appeared to be in the same transcription units with regulated genes for which the fold-change was ≥2 or when they were regulated by other sigma factors of sporulation.
