# Supplementary material for: Genome-Wide Analysis of Cell Type-Specific Gene Transcription during Spore Formation in Clostridium difficile
Source: PLoS Genet. 2013 Oct 3;9(10):e1003756. doi: 10.1371/journal.pgen.1003756 (PMC3789822; doi:10.1371/journal.pgen.1003756)
Supplement: Table S2 — List of genes controlled by σE in transcriptome. A gene is considered differentially expressed between the 630Δerm strain and the sigE mutant when the P value is <0.05 using the statistical analysis described in Materials and Methods. We did not include genes with a fold-change <2-fold. However, some genes with a fold-change less than 2-fold were included when they appeared to be in the same transcription unit with regulated genes for which the fold-change was ≥2 or when they were regulated by other sigma factors of sporulation. (PDF) [file pgen.1003756.s006.pdf]

Table S2. List of genes controlled by σ^E in transcriptome

Gene		Function	Expression ratio in transcriptome sigE/630Δerm
**Sporulation**			
CD0124	*spoIID*	peptidoglycane hydrolase SpoIID	0.24
CD1192	*spoIIIAA*	ATP-binding stage III sporulation protein	0.05
CD1193	*spoIIIAB*	Stage III sporulation protein AB	0.05
CD1194	*spoIIIAC*	Stage III sporulation protein AC	0.03
CD1195	*spoIIIAD*	Stage III sporulation protein AD	0.07
CD1196	*spoIIIAE*	Stage III sporulation protein AE	0.09
CD1197	*spoiIIIAF*	Stage III sporulation protein AF	0.08
CD1198	*spoIIIAG*	Stage III sporulation protein AG	0.02
CD1199	*spoIIIAH*	Stage III sporulation ratchet engulfment protein	0.05
CD0126	*spoIIID*	Transcriptional regulator SpoIIID	0.04
CD2629	*spoIVA*	Stage IV sporulation protein A	0.02
CD0783	*spoIVB*	Stage IV sporulation protein SpoIVB, S55 peptidase family	0.12
CD3567	*sipL*	Functional homolog to SpoIVD	0.05
CD2442	*spoIV*	Stage IV sporulation protein YqfD-like	0.14
CD2443		Conserved hypothetical protein YqfC-like	0.05
CD2967	*spoVFB*	Dipicolinate synthase subunit B	0.29
CD2968	*dpaA*	Dipicolinate synthase subunit A	0.30
CD1230	*sigK*	Fragment of RNA polymerase sigma-K factor SigK (Part1)	0.13
CD0782		Putative sporulation protein YunB	0.32
CD1168		Putative membrane protein, BDBH YlbJ involved in spore cortex formation	0.18
CD1613	*cotA*	Spore outer coat layer protein CotA	0.10
CD1511	*cotB*	Spore outer coat layer protein CotB	0.05
CD0598	*cotCB*	Spore-coat protein CotCB manganese catalase	0.18
CD2399		Conserved hypothetical protein	0.14
CD2400	*cotJB2*	Spore coat peptide assembly protein CotJB 2	0.10
CD2401	*cotD*	Spore coat protein CotD manganese catalase	0.11
CD1433	*cotE*	Spore coat protein CotE peroxiredoxin/chitinase	0.14
CD0213		Putative spore coat protein	0.42
CD0214		Conserved hypothetical protein	0.23
CD0551	*sleC*	Spore cortex-lytic enzyme pre-pro-form	0.15
CD0332	*bclA1*	Putative exosporium glycoprotein	0.21
CD3230	*bclA2*	Putative exosporium glycoprotein	0.12
CD3349	*bclA3*	Exosporium glycoprotein BclA3	0.01
CD3542	*spmA*	Spore maturation protein A	0.16
CD3541	*spmB*	Spore maturation protein B	0.11
CD0106	*cwlD*	Germination-specific N-acetylmuramoyl-L-alanine amidase, Autolysin	0.14
CD2246	*cspC*	Subtilisin-like serine germination related protease	0.16
CD2247	*cspBA*	Subtilisin-like serine germination related protease	0.11
CD3249	*sspB*	Small, acid-soluble spore protein alpha	0.04
CD1320		Putative peptidase, M16 family	0.25
CD1321		Putative sporulation protein	0.23
CD1322	*dapG*	Aspartokinase 1	0.32
CD1068		Putative polysaccharide biosynthesis/sporulation protein	0.23
CD2639		Putative cytotoxic factor	0.21
CD2640	*nrdR*	Transcriptional regulator, repressor NrdR family	0.23
CD2641		Putative sporulation protein	0.13
CD2439		Putative diacylglycerol kinase/undecaprenol kinase	0.45
CD3455		Putative carboxy-terminal protease, homolog of CtpB	0.25
CD3493		Putative membrane protein	0.18
CD3494		Putative spore protein	0.20
CD1234		Putative phage protein, skin element	0.22
**stress**			
CD1631	*sodA*	Superoxide dismutase (Mn)	0.21
CD3461	*mazF*	Endoribonuclease toxin	0.11
CD3462	*mazE*	Putative antitoxin EndoAI	0.05
CD2865		Putative bacterioferritin	0.19
**enveloppes**			
CD2664	*murE*	UDP-N-acetylmuramyl-tripeptide synthetase	0.45
CD3463	*alr2*	Alanine racemase 2	0.05
CD3464		Conserved hypothetical protein	0.04
CD2761		Putative N-acetylmuramoyl-L-alanine amidase	0.40
CD3007		Putative L,D-transpeptidases	0.37

CD2184		N-acetylmuramoyl-L-alanine amidase	**0.21**
CD2761		Putative N-acetylmuramoyl-L-alanine amidase	**0.40**
CD0514	cwpV	Cell surface protein	**0.63**
CD2445		Putative transmembrane signaling protein, TspO/MBR family	**0.07**
CD1845		Putative membrane protein Tn1549-like, CTn5-Orf1	**0.14**
CD1928		Putative membrane protein	**0.14**
CD1929		Putative membrane protein	**0.27**
CD2800		Putative membrane protein	**0.11**
CD1416		Putative membrane protein	**0.20**
CD0314		Putative membrane protein	**0.15**
CD1940		Putative membrane protein	**0.08**
CD3551.1		Putative membrane protein	**0.31**
CD0131		Putative membrane protein	**0.18**
CD1301		Putative membrane protein	**0.33**
CD2144		Putative membrane protein	**0.46**
CD2465		Putative amino acid/polyamine transporter	**0.20**
CD0902		Putative cation efflux protein	**0.29**
CD2833		Putative calcium-transporting ATPase	**0.22**
CD0760		Putative Ca2+/Na+ antiporter	**0.20**
CD3483		Putative zinc/iron permease	**0.34**
**metabolism**			
CD3635		Conserved hypothetical protein	**0.29**
CD3636		Putative membrane protein	**0.42**
CD3637		Putative NADPH-dependent FMN reductase	**0.27**
CD3638		Conserved hypothetical protein	**0.21**
CD3251		Putative dehydrogenase	**0.25**
CD3258		Iron hydrogenase	**0.08**
CD2428		Putative flavodoxin/ferredoxin oxidoreductase beta subunit	**0.38**
CD2429		Putative flavodoxin/ferredoxin oxidoreductase alpha subunit	**0.31**
CD2429.1		Putative 4Fe-4S ferredoxin, iron-sulfur binding domain protein, delta subu	**0.27**
CD2000	*isp*	Intracellular serine protease	**0.18**
CD3652		Putative peptidase, M1 family	**0.12**
CD1085		Putative membrane protein	**0.26**
CD1086		Putative peptidase, M20D family	**0.26**
CD3521		Putative peptidase T, M20B family	**0.33**
CD1746	*gltC*	Sodium/glutamate symporter	**0.20**
CD1555		Putative amino acid permease	**0.22**
CD1259	*brnQ-1*	Branched chain amino acid transport system carrier protein	**0.39**
CD1904		ABC-type transport system, permease	**0.26**
CD1891		Fragment of ABC-type transport system, substrate-binding protein (Part 1	**0.26**
CD1927	ytlC	putative ABC transporter component, ATP-binding	**0.30**
CD1319		Putative polysaccharide deacetylase	**0.06**
CD3248		Polysaccharide deacetylase	**0.09**
CD3257		Putative polysaccharide deacetylase	**0.11**
CD3032		Putative pyridoxal phosphate-dependent transferase	**0.32**
CD0982	*ubiA*	Putative UbiA prenyltransferase	**0.39**
CD2537		Putative membrane-associated 5'-nucleotidase/phosphoesterase	**0.15**
**miscellaenous**			
CD0749		Putative DNA helicase, UvrD/REP type	**0.32**
CD3235	*ssb*	Single-stranded DNA-binding protein	**0.03**
CD1167	*recV*	Tyrosine DNA recombinase, XerC/XerD family	**0.26**
CD1846		Putative conjugative transposon protein Tn1549-like, CTn5-Orf2	**0.15**
CD2864		Putative hydrolase	**0.08**
CD3298		Putative ATP/GTP-binding protein	**0.19**
CD1486		Putative ribosome recycling factor	**0.26**
**regulator**			
CD0757		Putative diguanylate kinase signaling protein	**0.45**
CD1616		Putative diguanylate kinase signaling protein	**0.37**
CD2637		Two-component sensor histidine kinase	**0.30**
**Proteins of unknown function**			
CD0129		Conserved hypothetical protein, DUF1256 family	**0.15**
CD0196		Fragment of conserved hypothetical protein, DUF111 family (part 2)	**0.18**
CD0311		Conserved hypothetical protein	**0.02**
CD0896		Conserved hypothetical protein	**0.05**
CD1063		Conserved hypothetical protein	**0.11**
CD1063.2		Conserved hypothetical protein	**0.01**
CD1063.3		Conserved hypothetical protein	**0.01**

CD1063.1	Conserved hypothetical protein	**0.17**
CD1065	Conserved hypothetical protein	**0.06**
CD1066	Conserved hypothetical protein	**0.16**
CD1067	Conserved hypothetical protein	**0.00**
CD1133	Conserved hypothetical protein	**0.12**
CD1581	Conserved hypothetical protein	**0.01**
CD1726	Conserved hypothetical protein	**0.05**
CD1880	Conserved hypothetical protein	**0.21**
CD1884	Conserved hypothetical protein	**0.11**
CD1930	Conserved hypothetical protein	**0.27**
CD2121	Conserved hypothetical protein	**0.23**
CD2374	Conserved hypothetical protein	**0.23**
CD2816	Conserved hypothetical protein	**0.11**
CD3234	Conserved hypothetical protein	**0.09**
CD3457	Conserved hypothetical protein	**0.05**
CD3465	Conserved hypothetical protein	**0.40**
CD3522	Conserved hypothetical protein	**0.09**
CD3580	Conserved hypothetical protein	**0.11**
CD3620	Conserved hypothetical protein, similar to YmaF	**0.34**

A gene is considered differentially expressed between the 630Δerm strain and the *sigE* mutant when the *P* value is <0.05 using the statistical analysis described in Materials and Methods. We did not include genes with a fold-change < 2-fold. However, some genes with a fold-change less than 2-fold were included when they appeared to be in the same transcription units with regulated genes for which the fold-change was ≥2 or when they were regulated by other sigma factors of sporulation.
