# Supplementary material for: Genome-Wide Analysis of Cell Type-Specific Gene Transcription during Spore Formation in Clostridium difficile
Source: PLoS Genet. 2013 Oct 3;9(10):e1003756. doi: 10.1371/journal.pgen.1003756 (PMC3789822; doi:10.1371/journal.pgen.1003756)
Supplement: Table S3 — List of genes controlled by σG in transcriptome. A gene is considered differentially expressed between the 630Δerm strain and the sigG mutant when the P value is <0.05 using the statistical analysis described in Materials and Methods. (PDF) [file pgen.1003756.s007.pdf]

Table S3. List of genes controlled by σG in transcriptome

Gene		Function	Expression ratio in transcriptome sigG/630Δerm
**Sporulation**			
CD2642	*sigG*	RNA polymerase sigma-G factor	**0.23**
CD0773	*spoVAC*	Stage V sporulation protein AC	**0.26**
CD0774	*spoVAD*	Stage V sporulation protein AD	**0.30**
CD0775	*spoVAE*	Stage V sporulation protein AE	**0.28**
CD1213	*spoIVB*	Stage IV sporulation protein B, peptidase S55 family	**0.60**
CD3499	*spoVT*	Stage V sporulation protein T	**0.22**
CD3249	*sspB*	Small, acid-soluble spore protein alpha	**0.07**
CD2688	*sspA*	Small, acid-soluble spore protein alpha	**0.02**
CD1290		Putative small acid-soluble spore protein SASP	**0.52**
CD3220.1		Small acid-soluble spore protein	**0.66**
**Stress**			
CD2845	*rbr*	Rubrerythrin	**0.27**
CD1631	*sodA*	Superoxide dismutase (Mn)	**0.32**
CD1567		putative manganese catalase	**0.27**
**Enveloppes**			
CD0784		Putative N-acetylmuramoyl-L-alanine amidase	**0.41**
CD1291	*dacF*	D-alanyl-D-alanine carboxypeptidase	**0.31**
CD2762	*uppS*	Putative undecaprenyl pyrophosphate synthetase	**0.62**
CD1430		Putative δ-lactam-biosynthetic de-N-acteylase	**0.21**
CD0792		Putative membrane protein, DUF81 family	**0.43**
CD0793		Putative membrane protein, DUF81 family	**0.33**
CD1788		Conserved hypothetical protein	**0.43**
CD1789		Putative membrane protein, DUF421 family	**0.61**
CD2051		Putative membrane protein	**0.52**
CD2315		Putative exported protein	**0.38**
CD2634		Conserved hypothetical protein	**0.65**
CD2635		Putative membrane protein	**0.50**
CD2636		Putative membrane protein	**0.34**
CD3551.1		Putative membrane protein	**0.18**
CD2465		Putative amino acid/polyamine transporter	**0.52**
**Metabolism**			
CD0684		Putative ATP-dependent peptidase, M41 family	**0.46**
CD3489		Putative oligoendopeptidase F, peptidase M3B family	**0.62**
CD2431		Putative nitrite/sulphite reductase	**0.54**
CD1676	*pcp*	Pyrrolidone-carboxylate peptidase	**0.66**
CD1677		Putative membrane protein	**0.51**
CD2841		Putative amidohydrolase	**0.57**
CD1543		putative FMN-dependent NADH-azoreductase	**0.43**
CD1707		Putative C4-dicarboxylate anaerobic carrier, DcuC family	**0.53**
**Translation**			
CD1486		Putative ribosome recycling factor	**0.17**
**Proteins of unknown function**			
CD0543		Conserved hypothetical protein	**0.57**
CD1297		Conserved hypothetical protein	**0.66**
CD1298		Conserved hypothetical protein	**0.55**
CD1301.1		Conserved hypothetical protein	**0.45**
CD1354		Conserved hypothetical protein	**0.48**
CD1463		Conserved hypothetical protein	**0.22**
CD1568		Conserved hypothetical protein	**0.60**
CD1880		Conserved hypothetical protein	**0.25**
CD2112		Conserved hypothetical protein	**0.26**
CD2245.1		Conserved hypothetical protein	**0.30**
CD2375		Conserved hypothetical protein	**0.24**
CD2808		Conserved hypothetical protein	**0.64**

CD2809	Conserved hypotheticalprotein, DUF1540 family	**0.45**
CD3610	Conserved hypothetical protein	**0.66**

A gene is considered differentially expressed between the 630Δerm strain and the *sigG* mutant when the P value is <0.05 using the statistical analysis described in Materials and Methods.
