# Supplementary material for: Genome-Wide Analysis of Cell Type-Specific Gene Transcription during Spore Formation in Clostridium difficile
Source: PLoS Genet. 2013 Oct 3;9(10):e1003756. doi: 10.1371/journal.pgen.1003756 (PMC3789822; doi:10.1371/journal.pgen.1003756)
Supplement: Table S4 — List of genes controlled by σK in transcriptome. A gene is considered differentially expressed between the 630Δerm strain and the sigK mutant when the P value is <0.05 using the statistical analysis described in Materials and Methods. We did not insert in this list genes with a fold-change <2-fold. However, some genes had a fold-change less than 2-fold but were included because they appeared to be in the same transcription unit with regulated genes for which the fold-change was ≥2 or because they were regulated by other sigma factors of sporulation. (PDF) [file pgen.1003756.s008.pdf]

Table S4. List of genes controlled by σK in transcriptome

Gene		Function	Expression ratio in transcriptome sigK/630Δerm
**Sporulation**			
CD1230		Fragment of RNA polymerase sigma-K factor SigK (Part1)	0.05
CD3569		Sporulation-specific protease, YabG-like protein	0.22
CD1613	*cotA*	Spore outer coat layer protein CotA	0.05
CD2399		Conserved hypothetical protein	0.01
CD2400	*cotJB2*	Spore coat peptide assembly protein CotJB 2	0.01
CD2401	*cotD*	Spore coat protein CotD manganese catalase	0.01
CD0596		Conserved hypothetical protein	0.02
CD0597	*cotJB1*	Spore coat peptide assembly protein	0.02
CD0598	*cotCB*	Spore-coat protein CotCB manganese catalase	0.01
CD1433	*cotE*	Spore coat protein CotE peroxiredoxin/chitinase	0.03
CD2968	*dpaA*	Dipicolinate synthase subunit A	0.08
CD2967	*spoVFB*	Dipicolinate synthase subunit B	0.09
CD0551	*sleC*	Spore cortex-lytic enzyme pre-pro-form	0.14
CD0332	*bclA1*	Putative exosporium glycoprotein	0.08
CD3230	*bclA2*	Putative exosporium glycoprotein	0.04
CD3349	*bclA3*	Exosporium glycoprotein BclA3	0.02
**Enveloppes**			
CD0514	*cwpV*	Cell surface protein	0.20
CD1898		Cell wall hydrolase, N-acetylmuramic acid L-alanine amidase	0.12
CD1897		Conserved hypothetical protein	0.13
CD2184		Putative N-acetylmuramoyl-L-alanine amidase (cell wall hydrolase)	0.13
CD2664	*murE*	UDP-N-acetylmuramyl-tripeptide synthetase	0.42
CD3007		Putative L,D-transpeptidases	0.48
CD2537		Putative membrane-associated 5'-nucleotidase/phosphoesterase	0.22
CD2458		Transporter, Major Facilitator Superfamily (MFS)	0.39
CD2459		Putative glucokinase, ROK family	0.41
CD0902		Putative cation efflux protein	0.17
CD1517	*feoB*	Ferrous iron transport protein B	0.05
CD1518	*feoA*	Ferrous iron transport protein	0.05
CD1904		ABC-type transport system, permease	0.13
CD2720		Putative transporter	0.16
CD1927	*ytlC*	Fragment of ABC transporter component, ATP-binding	0.08
CD1891		Fragment of ABC-type transport system, substrate-binding protein	0.08
CD2346		Putative membrane protein	0.24
CD2144		Putative membrane protein	0.12
CD1824		P-type calcium transport ATPase	2.69
CD0147		Putative transporter	2.25
**Metabolism**			
CD3350		Putative glycosyl transferase, family 2	0.09
CD3032		Putative pyridoxal phosphate-dependent transferase	0.11
CD1951		putative Acyl-CoA N-acyltransferase	0.19
CD0119	*glmM*	Phosphoglucosamine mutase	0.49
CD0120	*glmS*	Glucosamine--fructose-6-phosphate aminotransferase	0.52
CD0995	*serA*	Putative D-3-phosphoglycerate dehydrogenase	6.49
CD0996		Conserved hypothetical protein	3.08
CD2480		Putative hydrolase	2.03
**Miscellaenous**			
CD0749		Putative DNA helicase, UvrD/REP type	0.05
CD1845		Putative membrane protein Tn1549-like, CTn5-Orf1	0.17
CD1846		Putative conjugative transposon protein Tn1549-like, CTn5-Orf2	0.20
CD0564		putative ATP-dependent protease, Lon family	0.39
CD0309		Putative hydrolase, HAD superfamily, subfamily IB	0.38
**Regulator**			
CD2048		Transcriptional regulator, RpiR family	2.05
**Proteins of unknown function**			
CD0196		Fragment of conserved hypothetical protein, DUF111 family (part 2	0.18
CD0896		Conserved hypothetical protein	0.02
CD1063.1		Conserved hypothetical protein	0.21
CD1063.2		Conserved hypothetical protein	0.04
CD1063.3		Conserved hypothetical protein	0.04
CD1065		Conserved hypothetical protein	0.30
CD1067		Conserved hypothetical protein	0.03

CD1133	Conserved hypothetical protein	**0.16**
CD1581	Conserved hypothetical protein	**0.01**
CD2055	Conserved hypothetical protein	**0.33**
CD2409	Conserved hypothetical protein	**0.11**
CD3580	Conserved hypothetical protein	**0.12**
CD3613	Conserved hypothetical protein	**0.04**
CD3620	Conserved hypothetical protein	**0.04**
CD1286	Conserved hypothetical protein	**2.17**
CD1831.1	Conserved hypothetical protein	**2.36**

A gene is considered differentially expressed between the 630Δerm strain and the *sigK* mutant when the *P* value is <0.05 using the statistical analysis described in Materials and Methods. We did not insert in this list genes with a fold-change < 2-fold. However, some genes had a fold-change less than 2-fold but were included because they appeared to be in the same transcription units with regulated genes for which the fold-change was ≥2 or because they were regulated by other sigma factors of sporulation.
