# Supplementary material for: Genome-Wide Analysis of Cell Type-Specific Gene Transcription during Spore Formation in Clostridium difficile
Source: PLoS Genet. 2013 Oct 3;9(10):e1003756. doi: 10.1371/journal.pgen.1003756 (PMC3789822; doi:10.1371/journal.pgen.1003756)
Supplement: Table S5 — Validation of microarrays data by qRT-PCR on selected genes. qRT-PCR experiments were performed on two different RNA preparations for each mutant. The results presented corresponded to the mean of at least two independent experiments. (PDF) [file pgen.1003756.s009.pdf]

Table S5. Validation of Microarrays data by qRT-PCR on selected genes

Gene ID	Name	Expression ratio	
		**transcriptome[a]**	**qRT-PCR[b]**
**SigF-controlled genes**		*sigF*/630Δerm	*sigF*/630Δerm
CD0125	*spoIIQ*	**0.11**	0.08
CD0332	*bclA1*	**0.43**	0.23
CD0580	*gapN*	**0.36**	0.11
CD0783	*spoIVB*	**0.10**	0.12
CD0792		**0.39**	0.006
CD1067		**0.07**	0.22
CD1229		**0.42**	0.08
CD1290		**0.29**	0.006
CD1486		**0.10**	0.035
CD1613	*cotA*	**0.13**	0.04
CD1940		**0.25**	0.18
CD2470	*gpr*	**0.22**	0.001
CD2636		**0.20**	0.008
CD2856		**0.46**	0.24
CD3499	*spoVT*	**0.07**	0.004
CD3564	*spoIIR*	**0.43**	0.025
**SigE-controlled genes**		*sigE*/630Δerm	*sigE*/630Δerm
CD0126	*spoIIID*	**0.04**	0.001
CD0311		**0.02**	0.0002
CD0598	*cotCB*	**0.18**	0.09
CD1192	*spoIIIAA*	**0.05**	0.0003
CD1230	*sigK*	**0.13**	0.002
CD1511	*cotB*	**0.05**	0.009
CD1613	*cotA*	**0.10**	0.0035
CD1616		**0.37**	0.13
CD1746	*gltC*	**0.20**	0.0015
CD1940		**0.08**	0.002
CD2000	*isp*	**0.18**	0.04
CD2629	*spoIVA*	**0.02**	0.004
CD3521		**0.33**	0.17
**SigG-controlled genes**		*sigG*/630Δerm	*sigG*/630Δerm
CD0684		**0.46**	0.11
CD0773	*spoVAC*	**0.26**	0.04
CD0792		**0.43**	0.015
CD1213	*spoIVB*	**0.60**	0.12
CD1290		**0.52**	0.02
CD1430	*pdaA*	**0.21**	0.03
CD1486		**0.17**	0.02
CD2375		**0.24**	0.06
CD2636		**0.34**	0.03
CD2688	*sspA*	**0.02**	0.02
CD3249	*sspB*	**0.07**	0.08
CD3499	*spoVT*	**0.22**	0.08
**SigK-controlled genes**		*sigK*/630Δerm	*sigK*/630Δerm

CD0332	*bclA1*	**0.08**	0.04
CD0551	*sleC*	**0.14**	0.011
CD0598	*cotCB*	**0.01**	0.003
CD1067		**0.03**	0.1
CD1133		**0.16**	0.15
CD1433	*cotE*	**0.03**	0.01
CD1613	*cotA*	**0.05**	0.002
CD2401	*cotD*	**0.01**	0.006
CD3230	*bclA2*	**0.04**	0.01
CD3580		**0.12**	0.07
**SpoIIID-controlled genes**		*spoIIID*/630Δerm	*spoIIID*/630Δerm
CD1067		**0.03**	0.0003
CD1192	*spoIIIAA*	**3.85**	30
CD1511	*cotB*	**3.54**	5.6
CD2629	*spoIVA*	**3.54**	11
CD2375		**5**	3
CD2864		**4**	9
CD3349	*bclA3*	**0.19**	0.07
CD3490	*spoIIE*	**3.14**	5.5
CD3499	*spoVT*	**2.26**	3.7

a. Results from 4 independent experiments
green: genes significantly down-regulated (p.value < 0.05);
red: genes significantly up-regulated (p.value < 0.05)

b. Results from at least 2 independent experiments
