# Supplementary material for: Genome-Wide Analysis of Cell Type-Specific Gene Transcription during Spore Formation in Clostridium difficile
Source: PLoS Genet. 2013 Oct 3;9(10):e1003756. doi: 10.1371/journal.pgen.1003756 (PMC3789822; doi:10.1371/journal.pgen.1003756)
Supplement: Table S6 — Identification of promoters recognized by σF and σG. The transcriptional start sites and the −10 and −35 boxes are indicated in red and blue, respectively. The position of 5′ start was identified by RNA-seq analysis with indicated score corresponding to the read length (51 bases) coverage ratio for TAP+ and TAP− samples. For σF and σG, the genes underlined are those validated by in silico analysis (see Materials and Methods) and listed in Table S8. TSS can be visualized at https://mmonot.eu/COV2HTML/visualisation.php?str_id=-14. (PDF) [file pgen.1003756.s010.pdf]

Table S6. Identification of promoters recognized by σ[F] and σ[G]

gene		sense	TSS position	score	promoter sequences
**SigF-dependent promoters**					
CD0125	*spoIIQ*	1	170627	19	TAATTGTATAAAATGCCAAAAACACGTCAATAATCCAAATG
CD0347		1	422797	6.3	TTCACGATTAAAAAACATAA-ATTGGTAAATACTATAAGATA
CD0580	*gapN*	1	693165	3.68	GAAATGTTTACATAACGAATAAATGGCTATAATTTAAATA
CD0761		1	932667	4.6	TTCATGTATATTATTTAACTATACTGTTTAAAATATTAATTG
CD1229		1	1427634	4	AAATAGTATTAGCCCCTTAGAAATTGTCAATAATGAAATTA
CD1323	*tepA*	1	1533409	1.68	TATCTTAATATATTTGATTT-ATTTGGTAAAACTAAATTA
CD2470	*gpr*	-1	2852330	4	TTCTTGTTATAAAATACTAAATTTTGTTAACAATTATAATTA
CD2856		-1	3339897	2.2	ATTTAGAATAAATCCATGAA-TATGAGAAAAAATAAGTATA
CD3564	*spoIIR*	-1	4165994	4.8	ATTATGAATAAATTAAATTT-AATTGGACATAATTGATGATA
CD2686		-1	3106296	5.1	ACCAAGTATAAAAAATATTATAATTGTAAAACTTACAAA
CD2642	*sigG*	-1	3050598	25.84	AAAAAGTATAAAAAAATTGG-AAATGTAAATAATTTCAATA
found in silico for sigF					
**SigG-dependent promoters**					
**genes used for the consensus**					
CD0684		-1	830199	4.12	AACATGTATATTTTTTCCATGAAAAATAAATAATAAAAAATA
CD0773	*spoVAC*	1	944348	2.3	AATAGGTTTATTTTTTTAAA-ATTAACAAATAATACTTTA
CD1213	*spoIVB*	1	1411366	1.17	TAAATGAATAAATTCTCAGG-AAAAAGGAATTATAAATACA
CD1290		1	1498542	8.39	ACCAATTATAACAATCTTTT-ATTAGTAAAAACTACTTA
CD1463		1	1691613	3.18	ATAACATAATTTTTATTAAAATAATACAGATACTATTTATA
CD1543		1	1792456	4	CAAAAGAATAGAATGGGTATATTTGTGAAATAATAATA
CD1567		1	1814395	5.1	ATTTGGAATAGTATTACAAC-TTAAGTAAATAATAATTTTG
CD1568		1	1815249	2.09	TAAGTGTATATCTATTTGTT-TATTGCAAAACATATAATA
CD1880		1	2186344	1.25	ATTGTGTAAAGTGAGAATACTATTTTCTTATATTGGTAT
CD2112		-1	2441656	11.16	ATTACAAATAAATATATGTAC-TTTGGGAAAAATAAATATAG
CD2245.1		-1	2599068	15	CTTATGAATTTTATTTGTAA-AATTGCTAAAACTATTACA
CD2375		1	2741458	4.63	TAAGTGTATAAAATTTAGAT-AACTTCACATAATAAGTTTA
CD2465		-1	2846279	3.07	TAGACTAATATAAAAAAAAA-CTTTGGGAATACTAAAACCA
CD2688	*sspA*	-1	3107629	1.68	TACCTGAATTAAAAATGTTT-AAAATCACATAATATTATCA
CD2762		-1	3211281	51	GTTTTGATTAATAATGTAATATTTTGATAATAATACCTATTG
CD2808		-1	3278805	17	AAATTGTATAAAAACTTACAG-CTTGGAGAAAATAATAGA
CD2841		-1	3323998	3.84	ACAATGAATAGTATTTACTT-CTTGGCAAACAATAATTA
CD2845	*rbr*	1	3326890	4.62	ATTGAGCATAATTATATCTC-CTATTGGAATAATAAAT
CD3220.1		-1	3769140	9.93	TATTTGTATAAATTTATTTT-AATTGACAAAAATACAAATA
CD3249	*sspB*	1	3804245	3.08	TTTCTGTATATATTATTTTTATTTTGGAGAAATTAGTATTA
CD3489		-1	4079467	2.27	TAATTGAATAATATATCTAA-AAAAAGCCATCCTATTATT
CD3499	*spoVT*	-1	4088959	11	TTTTTGTATATTTTTCTTAAC-AATGGTAAAAATAAACTTA
CD3551.1		-1	4146872	12	AATACGTATTTCTTTTTTCA-TTTCGGGAAAACTAAACACA
CD0543		1	648057	4	TATATGGATAAAAAACCCTAGAAACTCCAAATATAATAAGA
CD1291	*dacF*	1	1498803	1.6	ATTTAGAATAAAAAAAGTTA-AATGTGAGAAAATAAGTTTAA
CD1354		1	1570520	2.96	ATTGATACAATTAATTTTTAATATTGGACATAATAAAATAG
CD1430	*pdaA*	-1	1658355	2	ACTATAAATATTCAAACTATATTATAAGAAATATATTTTAAA
CD1707		1	1981481	3.13	TTTGCTAATAAAAAATTGTAAAAAGGGAAAACTTTAGTCA
CD1789		-1	2072033	4	ACTTTGAATTAAATTTTTTA-TATAGGATATACTAACTAAGTG
CD3610		-1	4217455	4	AAGGTGATTAAATTACATAAATAAAGGCTATAATAGTATGG
**others**					
CD2636		-1	3046122	18.19	GAAGGGAATTTTTTTTGTTT-TTTGCACAAACTATATAA
CD1486		1	1721501	1.4	TTTTAGTAAATAAAATATAA-AAATATAGAAATACTAAATTA
CD2809		1	3278964	42	TAATGGTTATATTCTTGTAATTATATGGACATAATTTATAATA
found in silico for sigG					

The transcriptional start sites and the -10 and -35 boxes are indicated in red and blue, respectively.The position of 5' start was identified by RNA-seq analysis with indicated score corresponding to the read length (51 bases) coverage ratio for TAP+ and TAP- samples. For σF and σG, the genes underlined are those validated by *in silico* analysis (see Materials and Methods) and listed in Table S8. TSS can be visualized at https://mmonot.eu/COV2HTML/visualisation.php?str_id=-14.
