# Supplementary material for: Genome-Wide Analysis of Cell Type-Specific Gene Transcription during Spore Formation in Clostridium difficile
Source: PLoS Genet. 2013 Oct 3;9(10):e1003756. doi: 10.1371/journal.pgen.1003756 (PMC3789822; doi:10.1371/journal.pgen.1003756)
Supplement: Table S7 — Identification of promoters recognized by σE and σK. The transcriptional start sites and the −10 and −35 boxes are indicated in red and blue, respectively. The position of 5′ start was identified by RNA-seq analysis with indicated score corresponding to the read length (51 bases) coverage ratio for TAP+ and TAP− sample. For σE and σK, the genes underlined are those validated by in silico analysis (see Materials and Methods) and listed in Table S8. TSS can be visualized at https://mmonot.eu/COV2HTML/visualisation.php?str_id=-14. (PDF) [file pgen.1003756.s011.pdf]

Table S7. Identification of promoters recognized by σ^E and σ^K

Promoters recognized by SigE-RNA polymerase holoenzyme

Gene		sense	TSS position	score	promoters
**spacing 16-18 bp**					
CD0106	*cwlD*	1	123155	2.66	TATATGGAATATTATAGTGTTTTTATAAGTATACATATACA
CD0124	*spoIID*	1	169491	11.3	AGAAAGTTATAAATTAATAGGGTTGTTCATATACTTAAATA
CD0126	*spoIIID*	1	171391	44.64	AGCTTGTTATATTTTTTGGTGTTTTTACATATATATCATTA
CD0314		1	382298	1.74	AGTATGTTATAAATGCTTTGTGTAAATACATATAATAGTTTA
CD1167	*recV*	-1	1366292	3.54	TTTTTCCATTATATAATATTTCATTTATATTATATAATAAA
CD1192	*spoIIIAA*	1	1394269	9.67	TTTTGGTTATATTCCTTGTCCTTTATAAATAGAATTATATA
CD1198	*spoIIIAG*	1	1398222	9.72	TAATAGTACTAAAAGAGAGGAAGAAAACATAAACTCTAATG
CD1416		1	1640973	14.62	ACTTAGTAATTAATTAATAGGTTGTATCATAAACTTCTATTA
CD1726		1	1999622	1.24	ATAAATACATATAAAGATATTTTTAAGAATAAAATTTATTA
CD1746	*gltC*	-1	2023841	3.8	AATTATATCTACTGATTCTTTTTAATATACATTTATTAATA
CD1884		1	2190340	6.95	GATTGAGAATAGGACAATTTTTAAGATACATAAAATATAAAG
CD1940		-1	2238053	14,36	ATATAAAGATATATTTTACTATTTAAATGCATATCATTATA
CD2247	*cspBA*	-1	2604416	2.14	TCTAATACATTTTAGACTCACACAAAACATATACATTAATA
CD2429.1		-1	2805180	1.04	ATTGATGCATATATAGTATTTATACAAAATATAAATATTATATA
CD2629	*spoIVA*	-1	3039083	43.97	TTTTAAGAATATTAACCCTTAACTCTAAATATAATTTAATA
CD2864		-1	3350321	9.81	TTTTATTCATATATATAAAATAACCATCATACATTTTCTTG
CD2865		-1	3350950	3.51	TCTAAGTTATATGAAAAAGTTGTGATGCATACAATTAATA
CD3235	*ssb*	-1	3788231	3.85	GATTTGTAATAAAATACATCCCTTATAAATATATTTATTA
CD3251		-1	3806139	3.41	TATAAAGCATAATACAAGCTAATGAAATAATATATTATTGTA
CD3457		-1	4051218	33.32	TTTAGAGTCTAAGATAAGTTTATAAATCATATATTTAGAA
CD3464		-1	4057317	11.98	ATGATATAATAAAAGCTTTATACAATGAATAAACTGAAGTA
CD3494		-1	4084393	6.39	ATTTTAGAATAAAATTAATAGTCCTCTAGTATATTAATAGTA
CD3542	*spmA*	-1	4137832	19.99	TTTCAAGTATAAAGTATTACCTAACTATCATAAAATTATTTA
CD0129		-1	173974	4	AGTTAGGTCTTTTTAATGCTTTTTATTCATATATTTTTTTG
CD0311		-1	373834	2.73	GAATGCACATATTATAAACTTTTTTAATATATATATTATTA
CD1234		1	1437244	2.32	AAATAAGTATAAGACAAGTTTATGAGTTCATATTTTATAACA
CD1319		1	1529627	10.44	TTTAGTGCATAAACAGAGCTTATTTTTAATAGTATTAAATA
CD2000	*isp*	-1	2311655	7.84	TATGACTCCTAAAAATAAAAAATATATCATAAATATAAATA
CD2443		-1	2819700	1.52	CAATATGATTACCTTTTTCTTGTTTAAATAATATATTAGATATTA
CD2445		-1	2821093	5.22	TTTTGGGAATAACGAGTCATATTTGAGCATATCTATAATCA
CD2641		-1	3049708	27.8	TATAGGTCATTTTTTTTTACTTCGATACATATTTATTTTTA
CD2800		-1	3272107	3.07	TTAGTAATTTAAAAGCCTTTTCTCGAATATATATTAATTTG
CD2816		1	3286453	4	TTATCTTTATAAATTATAGTTTTAGATTTATAATTTATAAA
CD3248		-1	3804051	10	TTACAGTACTTTTTTGAGCTGAAAAAAATAAATTTTAAA
CD3258		-1	3816903	1.85	AATAATAAATAAAAAATAGTATCTTTGTAAATATTAAACATA
CD3298		-1	3861389	17.82	AAGCTGTAATAAAATTCTTATAATATGAATATCTTAATTAA
CD3483		-1	4070891	11.68	TTTAGGTCATGTTATCATATTCTATTGTATATAATTGTTA
CD3521		-1	4115392	3.76	TATTGATAATATTTAAATATTACACTACAATATATCTTG
CD3522		-1	4116812	8.44	AGAAGGTACTTTTTTTAATTTTGTTAACATATATTGTTTA
CD3567	*sipL*	-1	4169365	1.75	TAGGTGTAATTTTTTTTTTGCTCCTTAAATATAAATATAGTG
CD3638		-1	4248132	7.13	TTTTAAGTATATCTTTACATTAGATTTTATAAATTTATTAATA
**spacing 15 bp**					
CD3455		-1	4049892	46.84	TAAATGTAATAAAAATAACAGCTAGTCATATAATAATAATA
CD2833		-1	3311499	8.19	GAAAAGTAATATACAAGCTATCATAAGCATATAATTCCTTA
CD0760		1	931632	4	GAAAAAGCATACATCTTCACAAACTGAATATTTATTTAATA
CD1063		1	1255576	8.53	ATGTAGTAATAAAGTATTATTTGATGAATAAACTTTATTG
CD1230	*sigK*	-1	1444808	4	TATATGTTTTAAATTCTAAAAATATGCATATTATTTTATTA
CD1511	*cotB*	-1	1748867	4.2	TAATATACATAAATTTAAATATTACGAATAAAGTATTATA

found in silico for sigE

Promoters recognized by SigK-RNA polymerase holoenzyme

gene		sense	position +1	score	dRNA-seq
CD0120	*glmS*	1	163369	1.93	CTTATATGACCTAACTCTTAAAATATATAAATAATATTTTTAG
CD0551	*sleC*	-1	658278	3.55	ATTATTTAACAAATTTCAAGCTATATTCATATCTTGAAATA
CD0896		1	1079040	1.77	AATCATTAGCAAATATTACCCCTTAAACATAGTATATTTTA
CD0902		1	1086288	19.98	TAGAATGAACAAGTACAACCAATTTTGAATATAAATATAGTA
CD1063.1		1	1256034	1.03	TAGAAGTAACTTATCTGTCTATTACTACATATATTTAATTA
CD1063.2		1	1256423	1	ATACATTAGCAAAATGGAAACCAAATGCATAGTATATGTTA
CD1067		1	1260209	3.78	AGCAAGTAACAATTAAGATTATAAAAGCATAGTATTAATTA
CD1133		1	1332562	6.13	AATGACTCACATTTCTTGAGCTTTTTGAATATACTATAAATA
CD1433	*cotE*	-1	1664675	12.48	AAATAGTAACACTAATATGTATTAATTAATAGAATAAAATA
CD1613	*cotA*	-1	1868003	1.13	CTTTTGTAACATTTTTTTACATTATAAATATAATATTACTA
CD1891		-1	2196837	3.7	TTTTTGTAACAACTACAATAAATTTTTAATACTATATACTA

CD1898		-1	2201962	4.94	AAAAAGAAACAAAATTCTAGGTTATGCATATATTAAATAG
CD2144		1	2480394	3.26	TATCTGTAACAATTTATCCATTTTTACAATACTATAACTAATA
CD2409		-1	2781176	6.06	TAAGTGAAACGTATTTAATTATATTGCATATATTATTGTA
CD2664	*murE*	-1	3078113	4.3	AACCATTCACATAGTAAAAAATTCTTAATATAATATTATA
CD2968	*dpaA*	-1	3450268	7.42	TTTAGTGAACCAATGTATTGATAAAAACATATTGTATATAGTA
CD3569		-1	4170728	4	ATTAGGAAACAAAGTTTTATATCGAACATAGTATAAACTA
CD3580		-1	4180825	4	TACCATTAACATTATTACTGTTTTAAGCATATTATATTGTA
CD3613		1	4219362	5.26	TAGATGTAACAAAATCGCATCCATTTAAATATTGTATTTTA
CD0564		1	671798	5.24	GTATGATGACTAATAATAAGAGTCTGTTTCATATACTTTAAT
CD1230	*sigK*	-1	1444765	4.63	TAACAGTCAACTTGGATAAGTCTCCTTCATATAATTTAAAG
CD1518	*feoA*	-1	1760860	4	TAAAAGTAACAAGGTAATAATATAATGCATAGTTTAAAATA
CD1927	*ytlC*	-1	2225857	5.12	AAAATGGTACAAATAGGTCATATTGTTAATAACATAATATA
CD2184		-1	2528930	5.2	CACGGAGAACTTTTACCTTCTTCAATTAATATATTATTATTA
CD2459		-1	2837573	8.34	AAAAATTTGCATAGTGTAACATTTTAATATACTATATATATA
found in silico for sigK					
**candidates for SigK-dependent promoters**					
CD0332					tttac**AC**cagctaagccagtcaagg**AATAATTT**aaagtg
CD3230					ttatc**AC**ataagaaattgtcgatt**CATATAAT**tatatag
CD3349					aggtc**AC**aatcatactaaatttga**CATATTAT**atataga
CD1065					atatc**AC**tcacaattcaccacttc**AATATTAT**ttctata
CD2399					tagta**AC**gatttttaaatgattg**AATATTAT**acactaga
CD0596					taaag**AC**ctattaaaactaccagt**TATAATAT**agaacca

The transcriptional start sites and the -10 and -35 boxes are indicated in red and blue, respectively. The position of 5’ start was identified by RNA-seq analysis with indicated score corresponding to the read length (51 bases) coverage ratio for TAP+ and TAP- sample. For σE and σK, the genes underlined are those validated by in silico analysis (see Materials and Methods) and listed in Table S8. TSS can be visualized at https://mmonot.eu/COV2HTML/visualisation.php?str_id=-14.
