# Supplementary material for: Genome-Wide Analysis of Cell Type-Specific Gene Transcription during Spore Formation in Clostridium difficile
Source: PLoS Genet. 2013 Oct 3;9(10):e1003756. doi: 10.1371/journal.pgen.1003756 (PMC3789822; doi:10.1371/journal.pgen.1003756)
Supplement: Table S8 — In silico validation of promoters located upstream of genes identified in transcriptome as regulated by σF, σE, σG or σK. We analyzed the promoters identified by TSS mapping by an iterative in silico strategy (see Materials and Methods). The training sets of genes used to first construct the recognition profiles for σF, σE, σG and σK were highlighted in yellow. For each promoter the score was obtained as defined in materials and methods. Using the same profiles, we searched the intergenic regions (positions (−100 +10) relative to start codons) of six closely related Clostridium species, C. saccharolyticum, C. thermocellum, C. tetani, C. cellulolyticum, C. ljungdahlii, C. kluyveri. We also analyzed the B. subtilis promoters using the DBTBS database [96]. The absence of an orthologous gene is indicated by 0. When an orthologous gene is present the score and the locus-tag are indicated. (PDF) [file pgen.1003756.s012.pdf]

Table S8. ***In silico*** **validation of promoters located upstream of genes identified in transcriptome as regulated by σ^F, σ^E, σ^G or σ^K**

SigF-dependent promoter

locus_tag	promoter_score	NC_014376	C.saccharolytic	NC_009012	C.thermocellur	NC_004557	C.tetani	NC_011898	C.cellulolyticun	NC_014328	C.ljungdahlii	NC_011837	C.kluyveri	NC_000964	B.subtilis	dbtbs
CD630_01250	4.94	Closa_2891	4.80	Cthe_2617	5.08	CTC00316	3.68	Ccel_0282	5.13	CLJU_c02480	4.60	CKR_3248	4.87	0	0	0
CD630_03470	5.18	0	0	0	0	0	0	0	0	0	0	0	0	0	0	0
CD630_05800	4.41	Closa_1722	3.43	0	0	0	0	0	0	0	0	0	0	BSU02470	2.85	0
CD630_07610	5.07	Closa_3948	2.60	0	0	CTC01999	3.15	Ccel_2488	3.81	CLJU_c35420	4.10	CKR_2939	4.10	BSU04580	3.08	0
CD630_12290	4.51	Closa_2233	2.31	Cthe_1011	4.74	0	0	Ccel_1486	3.96	0	0	CKR_3147	3.31	0	0	0
CD630_13230	4.41	Closa_2132	3.62	Cthe_1097	4.57	CTC01283	?	Ccel_1703	4.71	CLJU_c13240	3.91	CKR_1336	3.19	BSU16790	4.39	0
CD630_24700	4.36	Closa_0874	3.76	Cthe_1038	4.20	CTC02040	4.17	Ccel_1978	4.06	CLJU_c07910	5.21	CKR_0808	4.47	BSU25540	3.64	BSU25540
CD630_26420	5.42	Closa_2347	3.67	Cthe_0448	4.56	CTC01128	3.65	Ccel_2061	4.56	CLJU_c12330	5.23	CKR_1238	5.23	BSU15330	4.13	BSU15330
CD630_28560	4.65	0	0	0	0	0	0	0	0	0	0	0	0	0	0	0
CD630_35640	4.86	Closa_0527	4.72	Cthe_1920	3.43	CTC00288	5.37	Ccel_0292	4.00	CLJU_c02150	5.39	CKR_3285	5.20	BSU36970	4.72	BSU36970
training set																

SigE-dependent promoter

locus_tag	promoter_score	NC_014376	C.saccharolytic	NC_009012	C.thermocellur	NC_004557	C.tetani	NC_011898	C.cellulolyticun	NC_014328	C.ljungdahlii	NC_011837	C.kluyveri	NC_000964	B.subtilis	dbtbs
CD630_01060	4.62	Closa_0335	3.49	Cthe_1804	4.05	CTC02570	3.68	Ccel_2890	3.55	CLJU_c40690	3.67	CKR_0251	4.55	BSU01530	3.36	BSU01530
CD630_01240	5.00	Closa_2893	2.53	Cthe_2616	3.69	CTC00315	4.44	Ccel_0281	3.73	CLJU_02470	4.17	CKR_3249	4.44	BSU36750	4.67	BSU36750
CD630_01260	4.45	Closa_0373	3.69	Cthe_2618	2.62	0	0	0	0	CLJU_02490	4.06	CKR_3247	3.62	BSU36420	3.20	0
CD630_03140	5.10	0	0	0	0	0	0	Ccel_0665	4.01	CLJU_01910	4.17	CKR_3313	**4.82**	BSU18190	4.35	0
CD630_11670	4.38	0	0	Cthe_2286	3.95	0	0	0	0	0	0	0	0	0	0	0
CD630_11920	4.49	Closa_3252	3.32	Cthe_0845	3.79	CTC01589	4.04	Ccel_1916	3.31	CLJU_11030	3.67	CKR_1115	4.44	BSU24430	4.47	BSU24430
CD630_11980	4.49	Closa_3246	3.17	Cthe_0839	3.34	CTC01584	3.62	Ccel_1910	4.07	CLJU_11090	3.28	CKR_1121	3.28	BSU24370	2.84	BSU24370
CD630_14160	4.62	Closa_2820	4.12	0	0	0	0	0	0	0	0	0	0	0	0	0
CD630_17260	4.82	0	0	0	0	0	0	0	0	CLJU_04710	4.44	0	0	0	0	0
CD630_17460	4.34	0	0	0	0	CTC02306	3.95	0	0	0	0	0	0	0	0	0
CD630_18840	5.10	0	0	0	0	0	0	0	0	0	0	0	0	0	0	0
CD630_19400	4.59	0	0	0	0	0	0	0	0	0	0	0	0	0	0	0
CD630_22470	4.24	Closa_1425	4.34	0	0	CTC02345	4.33	0	0	0	0	0	0	0	0	0
CD630_24291	4.44	0	0	0	0	0	0	0	0	0	0	0	0	0	0	0
CD630_26290	4.82	Closa_2078	3.32	Cthe_1021	3.69	CTC01140	4.17	0	0	CLJU_12490	4.44	CKR_1258	4.17	BSU22800	4.40	BSU22800
CD630_28330	5.10	0	0	0	0	0	0	0	0	0	0	0	0	0	0	0
CD630_28640	4.61	Closa_4245	4.50	Cthe_3133	4.05	CTC01150	4.06	Ccel_2497	3.95	CLJU_30810	3.40	CKR_0686	3.58	BSU10900	3.18	0
CD630_28650	4.88	Closa_1072	3.34	0	0	0	0	0	0	CLJU_19700	4.82	0	0	0	0	0
CD630_32350	4.55	Closa_1571	**4.45**	Cthe_0559	3.80	CTC02296	**4.72**	Ccel_1522	**4.44**	CLJU_34620	3.95	CKR_2845	3.36	0	0	0
CD630_32510	4.55	0	0	0	0	0	0	Ccel_1651	3.19	0	0	0	0	0	0	0
CD630_34550	4.34	Closa_0798	3.34	Cthe_1857	3.63	CTC02507	4.72	Ccel_2904	3.67	CLJU_03090	4.33	CKR_3153	3.69	BSU35240	4.25	0
CD630_34570	4.32	0	0	0	0	0	0	0	0	0	0	0	0	0	0	0
CD630_34640	4.72	0	0	0	0	CTC02514	4.06	0	0	CLJU_03020	4.22	CKR_3161	4.22	0	0	0
CD630_34940	3.31	0	0	Cthe_2659	5.10	0	0	Ccel_3083	4.13	CLJU_c41650	4.83	CKR_0134	4.83	BSU00600	4.37	BSU00600
CD630_35420	5.10	Closa_0042	4.06	Cthe_2099	4.62	CTC00238	4.45	Ccel_0165	4.45	CLJU_01700	4.07	CKR_3332	4.45	BSU23180	3.44	0
training set																

SigG-dependent promoter

locus_tag	promoter_weight	NC_014376	C.saccharolytic	NC_009012	C.thermocellur	NC_004557	C.tetani	NC_011898	C.cellulolyticun	NC_014328	C.ljungdahlii	NC_011837	C.kluyveri	NC_000964	B.subtilis	dbtbs
CD630_07730	4.12	Closa_1565	3.20	Cthe_0122	3.53	CTC02245	3.57	Ccel_2282	**4.27**	CLJU_c20910	3.25	CKR_1481	3.25	BSU23420	2.45	0
CD630_23750	4.74	Closa_2298	**4.59**	0	0	0	0	0	0	0	0	0	0	0	0	0
CD630_14300	4.62	0	0	Cthe_1385	**4.21**	0	0	Ccel_1457	3.02	0	0	0	0	BSU35300	3.85	0
CD630_26880	4.25	0	0	0	0	0	0	0	0	CLJU_c21860	3.54	CKR_1432	3.77	BSU09750	2.84	0
CD630_32490	4.00	Closa_1963	3.53	0	0	0	0	Ccel_1561	**4.19**	CLJU_c29720	2.94	CKR_3038	3.39	0	0	0
CD630_06840	4.62	0	0	0	0	0	0	0	0	0	0	0	0	0	0	0
CD630_12130	4.21	Closa_3227	3.70	Cthe_0813	3.11	CTC01570	**4.05**	Ccel_1895	3.47	CLJU_c11210	**4.05**	CKR_1133	**4.05**	BSU24230	3.94	0
CD630_12900	4.15	0	0	0	0	0	0	0	0	0	0	0	0	0	0	0
CD630_14630	4.14	0	0	0	0	0	0	0	0	0	0	0	0	0	0	0
CD630_14860	4.07	0	0	0	0	0	0	0	0	0	0	0	0	0	0	0
CD630_15430	4.54	0	0	0	0	0	0	Ccel_0642	**4.34**	CLJU_c25150	**4.58**	0	0	BSU33540	4.24	0

CD630_15670	4.54	0	0	0	0	0	0	0	0	CLJU_c18620	**4.10**	CKR_0429	**4.09**	BSU04430	3.75	0
CD630_15680	4.21	0	0	Cthe_0291	**4.62**	CTC00965	**4.28**	Ccel_0215	**4.26**	CLJU_c18640	**4.10**	CKR_1034	3.11	BSU40820	3.39	0
CD630_18800	4.36	0	0	0	0	0	0	0	0	0	0	0	0	0	0	0
CD630_21120	4.10	0	0	0	0	0	0	0	0	0	0	0	0	0	0	0
CD630_22451	4.02	0	0	0	0	0	0	Ccel_1165	3.36	0	0	0	0	0	0	0
CD630_24650	4.11	Closa_1536	3.48	0	0	CTC00932	**4.01**	0	0	CLJU_c24320	**4.34**	CKR_3010	3.55	BSU12860	3.60	0
CD630_26360	4.14	0	0	0	0	0	0	0	0	0	0	0	0	0	0	0
CD630_27620	4.04	Closa_0920	3.21	0	0	CTC01360	3.90	0	0	CLJU_c35470	**4.26**	CKR_2944	3.81	0	0	0
CD630_28080	4.28	0	0	0	0	0	0	0	0	0	0	0	0	0	0	0
CD630_28410	4.19	0	0	0	0	0	0	0	0	0	0	0	0	BSU29550	2.79	0
CD630_28450	4.62	Closa_2922	**4.15**	0	0	0	0	Ccel_0893	**4.27**	0	0	0	0	0	0	0
CD630_32201	4.58	Closa_2419	3.37	Cthe_1856	**4.67**	0	0	Ccel_2903	**4.74**	CLJU_c34580	**4.58**	CKR_2841	**4.58**	0	0	0
CD630_34890	4.22	0	0	0	0	0	0	0	0	0	0	0	0	0	0	0
CD630_34990	4.58	Closa_4072	**4.70**	Cthe_2655	**4.62**	CTC00196	**4.32**	Ccel_3088	**4.62**	CLJU_c41700	**4.32**	CKR_0129	**4.32**	BSU00560	3.24	0
CD630_35511	4.10	Closa_4144	**4.74**	0	0	0	0	Ccel_3342	**4.54**	CLJU_c06720	**4.26**	CKR_1348	3.39	BSU31380	4.30	0
training set																

SigK-dependent promoter

locus_tag	promoter_weight	NC_014376	C.saccharolytic	NC_009012	C.thermocellur	NC_004557	C.tetani	NC_011898	C.cellulolyticun	NC_014328	C.ljungdahlii	NC_011837	C.kluyveri	NC_000964	B.subtilis	dbtbs
CD630_01200	4.75	Closa_2018	3.08	Cthe_1162	2.76	CTC02543	3.20	Ccel_1205	3.14	CLJU_c40420	3.65	CKR_0268	3.77	BSU01780	2.93	0
CD630_05510	4.80	Closa_1279	4.17	0	0	CTC02343	4.28	0	0	0	0	0	0	0	0	0
CD630_08960	4.52	0	0	0	0	0	0	0	0	0	0	0	0	0	0	0
CD630_09020	4.79	0	0	0	0	0	0	0	0	0	0	0	0	0	0	0
CD630_10631	4.93	0	0	0	0	0	0	0	0	0	0	0	0	0	0	0
CD630_10632	4.52	0	0	0	0	0	0	0	0	0	0	0	0	0	0	0
CD630_10670	5.04	0	0	0	0	0	0	0	0	0	0	0	0	0	0	0
CD630_11330	4.74	0	0	0	0	0	0	0	0	0	0	0	0	0	0	0
CD630_14330	5.25	Closa_3862	**5.28**	Cthe_0270	3.16	CTC00894	4.39	Ccel_1550	3.81	0	0	CKR_0314	3.81	BSU14220	3.55	0
CD630_16130	5.45	0	0	0	0	0	0	0	0	0	0	0	0	0	0	0
CD630_18910	4.72	Closa_1551	4.22	Cthe_2575	3.39	CTC00407	3.14	Ccel_0878	3.43	CLJU_c34710	3.50	CKR_2853	2.98	BSU30595	3.91	BSU30595
CD630_18980	5.15	0	0	0	0	CTC01537	3.74	0	0	0	0	0	0	0	0	0
CD630_21440	4.72	Closa_2167	3.82	0	0	0	0	Ccel_3212	4.28	CLJU_c05850	**4.45**	0	0	BSU29070	3.16	0
CD630_24090	4.51	0	0	0	0	0	0	0	0	0	0	0	0	0	0	0
CD630_26640	5.05	Closa_2129	3.24	Cthe_0978	4.17	CTC01632	2.94	Ccel_0479	3.60	CLJU_c10570	3.07	CKR_1082	4.13	BSU15180	3.55	0
CD630_29680	4.64	Closa_0221	3.29	Cthe_0420	3.48	0	0	Ccel_1705	3.69	0	0	0	0	BSU16730	3.63	BSU16730
CD630_35690	4.97	0	0	Cthe_2400	4.48	CTC00279	4.16	Ccel_0237	3.00	CLJU_c02060	4.17	CKR_3293	4.17	BSU00430	2.66	BSU00430
CD630_35800	5.41	0	0	0	0	0	0	0	0	0	0	0	0	0	0	0
CD630_36130	5.14	0	0	0	0	0	0	0	0	0	0	0	0	0	0	0
**training set**																

We analyzed the promoters identified by TSS mapping by an iterative *in silico* strategy as described in materials and methods. The training sets of genes used to first construct the recognition profiles for σ^F, σ^E, σ^G and σ^K were highlighted in yellow. For each promoter the score was obtained as defined in materials and methods. Using the same profiles, we searched the intergenic regions (positions (–100 +10) relative to start codons) of six closely related *Clostridium* species, *C. saccharolyticum*, *C. thermocellum*, *C. tetani*, *C. cellulolyticum*, *C. ljungdahlii*, *C. kluyveri*. We also analyzed the *B. subtilis* promoters using the DBTBS database [73]. The absence of an orthologous gene is indicated by 0. When an orthologous gene is present the score and the locus-tag are indicated.
