# Supplementary material for: Genome-Wide Analysis of Cell Type-Specific Gene Transcription during Spore Formation in Clostridium difficile
Source: PLoS Genet. 2013 Oct 3;9(10):e1003756. doi: 10.1371/journal.pgen.1003756 (PMC3789822; doi:10.1371/journal.pgen.1003756)
Supplement: Table S9 — Complementation of the mutants inactivated for the sigma factors of sporulation. qRT-PCR experiments were performed on two different RNA preparations for each mutant and each complemented strain. Cells were harvested after 14 h of growth for the strain 630Δerm, the sigE and sigF mutants and the sigF mutant containing pMTL84121-sigF and the sigE mutant containing pMTL84121-sigE, after 20 h of growth for the strain 630Δerm, the sigG mutant and the sigG mutant containing pMTL84121-sigG and after 24 h of growth for the strain 630Δerm, the sigK mutant and the sigK mutant containing pMTL84121-sigKskin+. The results presented correspond to the mean of at least two independent experiments. (PDF) [file pgen.1003756.s013.pdf]

Table S9. Complementation of the mutants inactivated for the Sigma factors of sporulation

Complementation of the *sigF* mutant

		sigF/630Δerm	complemented *sigF*/630Δerm
CD2469	*spoIIP*	0.001	12
CD3564	*spoIIR*	0.03	9
CD3499	*spoVT*	0.004	19

Complementation of the *sigE* mutant

		sigE/630Δerm	complemented *sigE*/630Δerm
CD1198	*spoIIIAG*	0.002	2
CD0126	*spoIIID*	0.001	0.7
CD2629	*spoIVA*	0.004	2.5
CD1230	*sigK*	0.002	0.4

Complementation of the *sigG* mutant

		sigG/630Δerm	complemented *sigG*/630Δerm
CD2688	*sspA*	0.02	0.7
CD3249	*sspB*	0.07	1.2
CD3499	*spoVT*	0.08	0.5
CD3551.1		0.006	1.3

Complementation of the *sigK* mutant

		sigK/630Δerm	complemented *sigK*/630Δerm
CD2401	*cotD*	0.005	0.15
CD0551	*sleC*	0.011	0.25
CD1433	*cotE*	0.01	0.15
CD1613	*cotA*	0.0005	0.05

qRT-PCR experiments were performed on two different RNA preparations for each mutant and each complemented strain. Cells were harvested after 14h of growth for the strain 630Δerm, the sigE and sigF mutants and the sigF mutant containing pMTL84121-sigF and the sigE mutant containing pMTL84121-sigE after 20h of growth for the strain 630Δerm, the sigG mutant and the sigG mutant containing pMTL84121-sigG and after 24 h of growth for the strain 630Δerm, the sigK mutant and the sigK mutant containing pMTL84121-sigK. The results presented corresponded to the mean of at least two independent experiments.
