# Supplementary material for: Genome-Wide Analysis of Cell Type-Specific Gene Transcription during Spore Formation in Clostridium difficile
Source: PLoS Genet. 2013 Oct 3;9(10):e1003756. doi: 10.1371/journal.pgen.1003756 (PMC3789822; doi:10.1371/journal.pgen.1003756)
Supplement: Table S10 — List of genes controlled by SpoIIID in transcriptome. RNA was extracted from strain 630Δerm strain and the spoIIID mutant after 15 h of growth in SM medium. A gene is considered differentially expressed between the 630Δerm strain and the spoIIID mutant when the P value is <0.05 using the statistical analysis described in Materials and Methods. (PDF) [file pgen.1003756.s014.pdf]

Table S10. List of genes controlled by SpoIIID in transcriptome

Gene		Function	Expression ratio in transcriptome *spoIIID*/630Δerm
**sporulation**			
CD1214	*spo0A*	Stage 0 sporulation protein A	**0.47**
CD2644	*spoIIGA*	Sigma-E factor processing peptidase	**2.36**
CD2643	*sigE*	RNA polymerase sigma-E factor	**1.85**
CD2642	*sigG*	RNA polymerase sigma-G factor	**2.72**
CD3490	*spoIIE*	Phosphoprotein phosphatase	**3.14**
CD3563	*sleB*	Spore-cortex-lytic protein	**1.94**
CD0125	*spoIIQ*	Stage II sporulation protein Q	**3.02**
CD1192	*spoIIIAA*	Stage III sporulation protein AA	**3.85**
CD1193	*spoIIIAB*	Stage III sporulation protein AB	**3.81**
CD1194	*spoIIIAC*	Stage III sporulation protein AC	**4.91**
CD1195	*spoIIIAD*	Stage III sporulation protein AD	**3.44**
CD1196	*spoIIIAE*	Stage III sporulation protein AE	**2.42**
CD1197	*spoiIIIAF*	Stage III sporulation protein AF	**3.63**
CD1198	*spoIIIAG*	Stage III sporulation protein AG	**7.75**
CD1199	*spoIIIAH*	Stage III sporulation protein AH	**3.31**
CD2629	*spoIVA*	Stage IV sporulation protein A	**3.54**
CD3567	*sipL*	SpoIVA-Interacting protein, coat localization	**3.06**
CD3541	*spmB*	Spore maturation protein B	**2.04**
CD1511	*cotB*	Spore outer coat layer protein CotB	**3.54**
CD2688	*sspA*	Small, acid-soluble spore protein alpha	**13.45**
CD3249	*sspB*	Small, acid-soluble spore protein alpha	**11.62**
CD3499	*spoVT*	Stage V sporulation protein T	**2.26**
CD3349	*bclA3*	Exosporium glycoprotein BclA3	**0.19**
**stress**			
CD1631	*sodA*	Spore coat protein, superoxide dismutase	**2.87**
CD2865		Putative bacterioferritin	**2.20**
**enveloppes**			
CD0147		Putative transporter	**20.56**
CD1416		Putative membrane protein	**2.24**
CD1928		Putative membrane protein	**3.05**
CD2445		Transmembrane signaling protein, TspO	**2.37**
CD2737		Putative nitrilase/cyanide hydratase	**2.25**
CD2738		Putative cytosine permease	**2.14**
CD2833		Putative calcium-transporting ATPase	**2.25**
CD2800		Putative membrane protein	**2.60**
CD3073		Putative membrane protein	**2.19**
CD2664	*murE*	UDP-N-acetylmuramyl-tripeptide synthetase	**1.93**
CD0090	*prlA*	Preprotein translocase SecY subunit	**0.44**
**metabolism**			
CD0740		Putative PLP-dependent aminotransferase	**2.17**
CD0777		Putative membrane protein	**1.96**
CD0778		Conserved hypothetical protein	**2.15**
CD0779		Putative amidohydrolase, M20D peptidase family	**2.13**
CD0780		Conserved hypothetical protein, DUF1177 family	**2.46**
CD0994		Putative serine-pyruvate aminotransferase	**1.91**
CD0995	*serA*	Putative D-3-phosphoglycerate dehydrogenase	**2.95**
CD0996		Conserved hypothetical protein	**2.22**
CD1319		Putative polysaccharide deacetylase	**3.24**
CD1697	*ribH*	6,7-dimethyl-8-ribityllumazine synthase	**15.04**
CD1698	*ribBA*	Riboflavin biosynthesis protein ribBA	**5.68**
CD1699	*ribE*	Riboflavin synthase alpha subunit	**3.84**
CD1700	*ribD*	Riboflavin biosynthesis protein ribD	**3.88**
CD1749		Putative 2-hydroxyacyl-CoA dehydratase	**1.94**
CD1750		Putative CoA enzyme activase	**2.20**

CD1767	*gapB*	Glyceraldehyde-3-phosphate dehydrogenase	**2.70**
CD2387		α-hydroxy acid dehydrogenase, FMN-dependent	**2.02**
CD2819		Putative amino acid racemase	**2.25**
CD2864		Putative hydrolase	**4.00**
CD3236		Putative membrane protein	**1.89**
CD3238		Putative component of proline reductase prdE-like	**1.96**
CD3241	*prdB*	Proline reductase (selenocysteine)	**2.07**
CD3243		Conserved hypothetical protein	**2.19**
CD3244	*prdA*	D-proline reductase proprotein prdA	**2.09**
CD3248		Polysaccharide deacetylase	**2.27**
CD3257		Putative polysaccharide deacetylase	**2.13**
CD3258		Iron hydrogenase	**2.26**
CD3489		Putative oligoendopeptidase F, peptidase M3B famil	**2.29**
CD2323		Putative sugar-phosphate dehydrogenase	**0.53**
CD2324		Putative sugar-phosphate dehydrogenase	**0.44**
CD2325	*gatC*	PTS system, galactitol-specific IIC component	**0.47**
CD2326		PTS system, lactose/cellobiose specific IIB compone	**0.47**
CD2327	*gatA*	PTS system, galactitol-specific IIA component	**0.48**
CD2600	*cstA*	Carbon starvation protein, CstA	**0.42**
**regulators**			
CD2214	*sinR*	Transcriptional regulator, HTH-type	**8.23**
CD2215		Transcriptional regulator, HTH-type	**8.22**
**miscellaenous**			
CD0356	*xis*	Excisionase Tn916-like, CTn1-Orf2	**2.45**
CD3235	*ssb*	Single-stranded DNA-binding protein	**3.63**
CD2517.1		Putative phage protein	**0.25**
**proteins of unknown function**			
CD0311		Conserved hypothetical protein	**4.12**
CD1063		Conserved hypothetical protein	**2.95**
CD1463		Conserved hypothetical protein	**2.41**
CD1726		Conserved hypothetical protein	**3.87**
CD1941		Conserved hypothetical protein	**2.38**
CD2046		Conserved hypothetical protein	**2.25**
CD2098		Conserved hypothetical protein	**2.09**
CD2112		Conserved hypothetical protein	**2.76**
CD2245.1		Conserved hypothetical protein	**3.35**
CD2366		Conserved hypothetical protein	**2.76**
CD2369		Conserved hypothetical protein	**2.70**
CD2375		Conserved hypothetical protein	**5.09**
CD2409		Conserved hypothetical protein	**2.28**
CD2752		Conserved hypothetical protein	**2.30**
CD2808		Conserved hypothetical protein	**3.04**
CD2809		Conserved hypothetical protein	**2.52**
CD3457		Conserved hypothetical protein	**2.62**
CD3610		Conserved hypothetical protein	**2.13**
CD3618		Conserved hypothetical protein	**2.31**
CD1067		Conserved hypothetical protein	**0.03**
CD2216		Conserved hypothetical protein	**0.39**

RNA was extracted from strain 630Δerm strain and the *spoIIID* mutant after 15 h of growth in a sporulation medium. A gene is considered differentially expressed between the 630Δerm strain and the *spoIIID* mutant when the P value is <0.05 using the statistical analysis described in Materials and Methods
