# Supplementary material for: Genome-Wide Analysis of Cell Type-Specific Gene Transcription during Spore Formation in Clostridium difficile
Source: PLoS Genet. 2013 Oct 3;9(10):e1003756. doi: 10.1371/journal.pgen.1003756 (PMC3789822; doi:10.1371/journal.pgen.1003756)
Supplement: Table S11 — Control of expression of sporulation genes by SpoIIID, SpoVT or SpoIIR. Cells of the 630Δerm strain and of the spoIIID mutant were harvested after 15 h or 24 h of growth in SM medium. Cells of the 630Δerm strain and of the spoVT mutant were harvested after 15 h or 20 h of growth in SM medium. Cells of the 630Δerm strain and of the spoIIR mutant were harvested after 14 h of growth in SM medium. qRT-PCR experiments were performed on two different RNA preparations. The results presented corresponded to the mean of at least two independent experiments. NR = not regulated. (PDF) [file pgen.1003756.s015.pdf]

Table S11. Control of expression of sporulation genes by SpoIIID, SpoVT or SpoIIR

**Positive control by SpoIIID**			
gene		***spoIIID*/630Δ*erm***	***spoIIID*/630Δ*erm***
		**15 h of growth**	**24 h of growth**
CD1230	*sigK*	0.04	0.0004
CD1231		NR	NR
CD1613	*cotA*	NR	0.0003
CD0598	*cotCB*		3 x 10-4
CD1433	*cotE*		0.0006
CD2401	*cotD*		8 x 10-4
CD0551	*sleC*		0.0004
CD0332	*bclA1*		0.01
CD3230	*bclA2*		0.03
CD3349	*bclA3*	0.07	0.0005
CD3580			0.03
CD1067		0.0003	0.0003
CD1133			0.03
**Control by SpoVT**		***spoVT*/630Δerm**	
		**15 h of growth**	
CD2470	*gpr*	9	
CD3564	*spoIIR*	4	
		**20 h of growth**	
CD2688	*sspA*	0.014	
CD3249	*sspB*	0.08	
**Control by SpoIIR**		***spoIIR*/630Δerm**	
		**14 h of growth**	
CD1192	*spoIIIAA*	0.1	
CD2629	*spoIVA*	0.12	
CD0126	*spoIIID*	0.06	
CD1230	*sigK*	0.04	

Cells of the 630Δerm strain and of the *spoIIID* mutant were harvested after 15h or 24h of growth in SM medium. Cells of the 630Δerm strain and of the *spoVT* mutant were harvested after 15 h or 20 h of growth in SM medium. Cells of the 630Δerm strain and of the *spoIIR* mutant were harvested after 14 h of growth in SM medium. qRT-PCR experiments were performed on two different RNA preparations. The results presented corresponded to the mean of at least two independent experiments.
