# Supplementary material for: Genome-Wide Analysis of Cell Type-Specific Gene Transcription during Spore Formation in Clostridium difficile
Source: PLoS Genet. 2013 Oct 3;9(10):e1003756. doi: 10.1371/journal.pgen.1003756 (PMC3789822; doi:10.1371/journal.pgen.1003756)
Supplement: Table S12 — List of oligonucleotides. (PDF) [file pgen.1003756.s016.pdf]

Table S12. List of oligonucleotides

Description	SEQUENCE (5' TO 3')	Primers
5' CD0125-spoIIQ	GCAGTAGGTGGAGTTTGGTTT	LS156b
3' CD0125	TGAATCCGTTGCAGTAGGG	LS157
5' CD0126-spoIIID	GGGCCATAGTGGTAGCAAAA	LS102
3' CD0126	TGGCAAGGGATGGATTTATT	LS103
5' CD0332-bclA1	GCGGAGGAGTACTTTCTGGA	LS206
3' CD0332	GCTGATTGCCCATTTCGTAT	LS207
5' CD0551-sleC	CCAATGTCCTGACCAAATGA	LS147
3' CD0551	CTGCCCAGAAGAACCAACTT	LS148
5' CD0598-cotCB	TTAAGGCTGCTGGACTTGGT	LS158
3' CD0598	AGTTACCGAATCGCCAAAGA	LS159
5' CD0684	AATCCAGCTCCTGTCTTTGC	LS218
3' CD0684	CGTCTAATCCAGCAACGTCTC	LS219
5' CD3499-spoVT	TTGGAAGGGTAGTTATCCCAAA	IMV550
3' CD3499	GCATATTCTTGCGAAAATTCA	IMV551
5' CD1067	AGCATCACCAAATCCAATCC	LS151
3' CD1067	AACTTGGCTTTCCACTTCCA	LS152
5'CD1133	TTGATATGGCCCACAAAGAA	LS166
3'CD1133	CACTTTCTTGATATGCACACTTTTT	LS167
5' CD1192-spoIIIAA	CACCACCTCAATGTGGAAAA	LS100
3' CD1192	GCTCCTGCTATCTCATTACGC	LS101
5' CD1230-sigK	GAAAAACCCTTAACCCCTGA	LS155
3' CD1230	TCATCCTGATCTTCCGTTGA	LS156
5' CD1430	TTCAAAAAGCCATCCATCAA	LS214
3' CD1430	TCGCTAAATTTACCCATTGGA	LS215
5' CD1433-cotE	CAGGCCCAAATGGTAGAAAA	LS143
3' CD1433	GAAGGCATTCCAGCATTCTC	LS144
5' CD2470-gpr	TGCATTGGCTTCAAGAAAAA	LS114
3' CD2470	GCAATAACATCCACGCCTAAA	LS115
5' CD1486	TAATCGCTGAAGGGGAAAGA	LS220
3' CD1486	CCAACATCTTCAGTTCCATGA	LS221
5' CD1511-cotB	TTGCTATGGAAGCTGACCACT	LS141
3' CD1511	TTGTTCTGCAACTGATGTGAGA	LS142
5' CD1613-cotA	GCTGCATCTACTCCATTAGCAA	LS145
3' CD1613	GCAATCATCACAATCGCAGT	LS146
5' CD1940	GGATTTTTGGGAATAATTTCAAG	LS232
3' CD1940	TTTTGTCGGATGTTTCTGGA	LS233
5' CD2000	GGGCAAGAGAGGAGTTTGAA	LS226
3' CD2000	TCCATTTCCTTGTTCCGTTC	LS227
5' CD2375	AAAGTAGATGGAATGCAAGCAA	LS212
3' CD2375	CATTTTACAGCTTCACAATGAATG	LS213
5' CD2401-cotD	AAAGCTGCAGGTCTTGGTTC	LS160
3' CD2401	AAATCGGTTACGGGATTTGA	LS161
5' CD2629-spoIVA	CCCCAAAGTGGTTCAGGTAA	LS106
3' CD2629	TGCCCTAAAGCTCCTTCAAC	LS107
5' CD2688-sspA	CCAGGAGCAAAAGAGGCTTTA	IMV552
3' CD2688	TCCAGCCATTTGTTGTTCAG	IMV553

5' CD3230-bclA2	AGCAGCAAACAATGCACAAT	LS208
3' CD3230	TCCCTCCAACTGCAAGATTT	LS209
5' CD3249-sspB	CAGTAGTTCCAGAAGCAAAAGC	IMV554
3' CD3249	TTCCAGCCATTTGTCTTTCA	IMV555
5' CD0773-spoVAC	AGGTGCTAGTTCTGCAACATCA	LS216
3' CD0773	ACCAGCTCCAGCTCTTTTTC	LS217
5' CD3564-spoIIR	GTTCTTCCAGCTGGTGAATATAAA	LS112
3' CD3564	CAACAAAACATAGTGGAGGGAAC	LS113
5' CD3551.1	AACTGGGGTGCAATTGGTAT	LS230
3' CD3551.1	TCCAGCTAAACCTACAATGAAGT	LS231
5' CD3580	CGTTGACGAAATAGCACCTG	LS164
3' CD3580	TGGGGAAATACAATAGAAGCGTA	LS165
5' CD1213-spoIVB	GGTCATGCCATAAAAGATTCG	LS200
3' CD1213	CCCATTAAATTGCTGTCATTAAAA	LS201
5' CD2636	CAAACAATACCCATCAAGACCA	LS245
3' CD2636	GTCAAGGCTGCAAACTCCTT	LS246
5' CD0792	TTTGGGGTTTTAGGAGCTGT	LS243
3' CD0792	GGCTTTTTATCGGATTTTGC	LS244
5' CD1290	TGGATGATGTTTCAAGACAAAA	LS239
3' CD1290	AAACACCTTTATTAGACGCATTTTC	LS240
5' CD0311	TTCCCCAAGATGAATTGAGAA	LS257
3' CD0311	TGTTAGGCAAGTTGTTTGGAGA	LS258
5' CD1616	TGGCAAAAGAGTTGGGTATG	IMV617
3' CD1616	CTTCAATTGGCATTGGCTTT	IMV618
5' CD1746	AGGGCTTATGTGTGGAAATG	LS247
3' CD1746	TAATCCAACCCCAACTGCTC	LS248
5' CD3521	GGTGGTTCTCCAGGTGAAAT	LS259
3' CD3521	CCAGCTTCAGGTTCAAGACC	LS260
5' CD0596	TGGGATATTTAAAAGAGGATAAACAA	LS261
3' CD0596	TGCTGTTTTTGGATTGTATGTG	LS262
5' CD0580-gapN	GTATGGGCTGAAATGCCTGT	LS241
3' CD0580	CTTCTGCCCTTGCAGATTTT	LS242
5' CD2856	AAACAAGGGCATTTTCAAGAAG	LS255
3' CD2856	TGCAAATATATTATTGGGGTCAA	LS256
5' CD0783-spoIVB'	GCTTTTAGCTCAAACTGGTGGT	LS249
3' CD0783	CCTACAGCTGGGTCGTTTTC	LS250
5' CD1229	GCAGCAATAATCTCGGAAGC	LS253
3' CD1229	CCTGGTGGATATGTCGCTTT	LS254
IBS-*spoIIR*-39a	AAAAAAGCTTATAATTATCCTTAGATATCAGACTTGTGC GCCCAGATAGGGTG	CT025
EBS1d-*spoIIR*-39a	CAGATTGTACAAATGTGGTGATAACAGATAAGTCAGACT TAATAACTTACCTTTCTTTGT	CT026
EBS2-*spoIIR*-39a	TGAACGCAAGTTTCTAATTTCGATTATATCTCGATAGAG GAAAGTGTCT	CT027
IBS-*spoIIID*-39s	AAAAAAGCTTATAATTATCCTTAATAGTCGTAGCAGTGC GCCCAGATAGGGTG	LS171
EBS1d-*spoIIID*-	CAGATTGTACAAATGTGGTGATAACAGATAAGTCGTAGC AAATAACTTACCTTTCTTTGT	LS172

EBS2-*spoIIID*-39s	TGAACGCAAGTTTCTAATTTCGGTTACTATCCGATAGAG GAAAGTGTCT	LS173
5’ CD0126-*spoIIID*	ATCTCATATAGAGGAAAGG	LS184
IBS-*spoVT*-157a	AAAAAAGCTTATAATTATCCTTACATTTCATTCACGTGC GCCCAGATAGGGTG	LS174
EBS1d-*spoVT*-157a	CAGATTGTACAAATGTGGTGATAACAGATAAGTCATTCA CCTTAACTTACCTTTCTTTGT	LS175
EBS2-*spoVT*-157a	TGAACGCAAGTTTCTAATTTCGGTTAAATGTCGATAGAG GAAAGTGTCT	LS176
3’ CD0126-*spoIIID*	CATTTTTAACTTCTTTGGC	LS185
5’ CD3499-*spoVT*	TTCACAGCAAAAGATGGAG	LS186
3’ CD3499-*spoVT*	ACACTCCATATCCTAGTAC	LS187
3’BamHI-spoIIR	CGGGATCCTTAATATTTTCTGTTGGTTC	IMV641
5’XhoI-spoIIR	CCGCTCGAGATAAGGCTATGATTAGT	IMV642
5’XhoI-*spoVT*	CCGCTCGAGGTAGAAAAAGATGAAAAAG	IMV644
3’BamHI-*spoVT*	CGGGATCCTACGATTACACCTGCTA	IMV648
5’XhoI-*spoIIID*	CCGCTCGAGCAATGAGTCTAATTAAGT	IMV647
3’BamHI-*spoIIID*	CGGGATCCAAATCTCGCTTTAATCAA	LS283
5’ CD3564-spoIIR	AAATTAAGATTAGATTGGGACTA	IMV649
Sequencing pMTL007	TTAAGGAGGTGTATTTCATATGACCATGATTACG	pMTL007 Fw
Sequencing pMTL007	AGGGTATCCCCAGTTAGTGTTAAGTCTTGG	pMTL007 Rev
5’-promoter spoIIR-EcoRI Fw	AAGCACTTTGAATTCCGATGTAGATGCGTT	PspoIIR-SNAP EcoRIFw
3’-promoter spoIIR-XhoI Rev	ATTGAAAACTCGAGCTCAAGATAAATTTATATC	PspoIIR-SNAP XhoIRev
5’-sigF-NcoI	GAGCTCCATGGGAGGTTTTATTAATGGAAGTAACTG	sigF-pet28a Fw
3’-sigF-XhoI	TGCCTCGAGCGATATATATTCTTTTAACTTAG	sigF-pet28a Rev
5’-sigE-NcoI	GAGCTCCATGGGCTTTATTACTATGTTAGGTATTA	sigE-pet28a Fw
3’-sigE-XhoI	TGCCTCGAGTACAAATTTTTTCATTTCTTTTTGC	sigE-pet28a Rev
Intron probe	ATCTGTAGGAGAACCTATGGGAAC	OBD522
Intron probe	CACGTAATAAATATCTGGACGTAAAA	OBD523

[A] RESTRICTION SITES UNDERLINED
